# Supplementary figures and images for: Increased mitochondrial protein import and cardiolipin remodelling upon early mtUPR
Source: PLoS Genet. 2021 Jul 2;17(7):e1009664. doi: 10.1371/journal.pgen.1009664 (PMC8282050; doi:10.1371/journal.pgen.1009664)

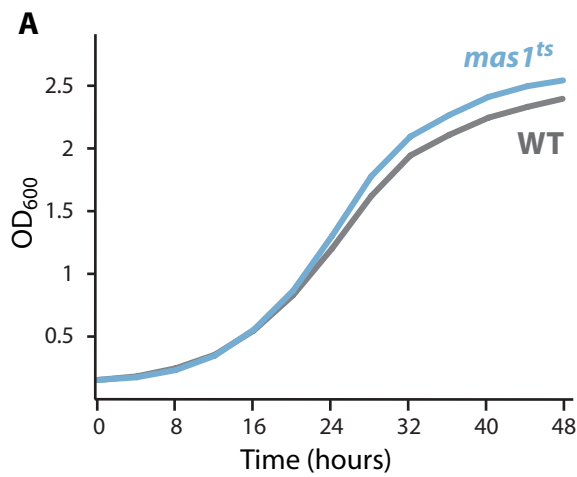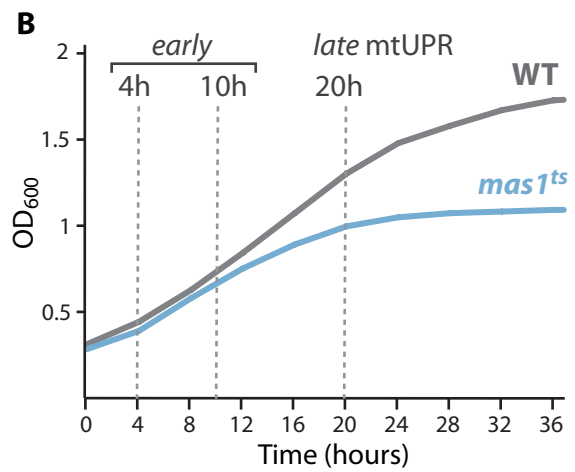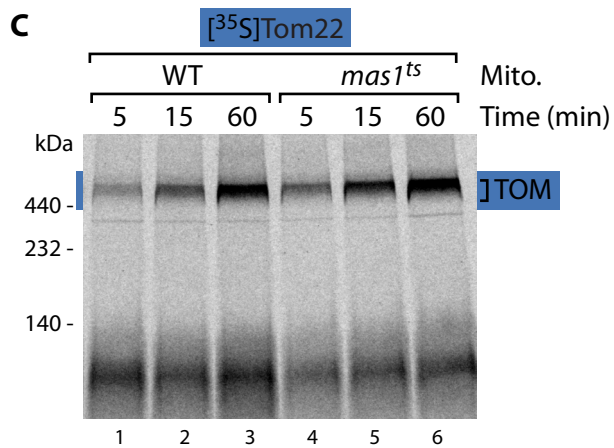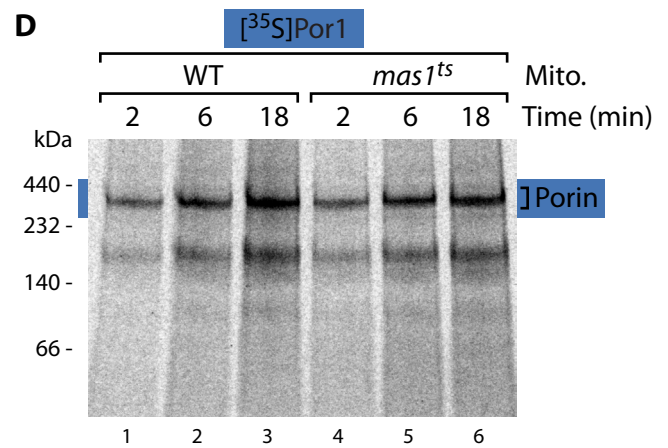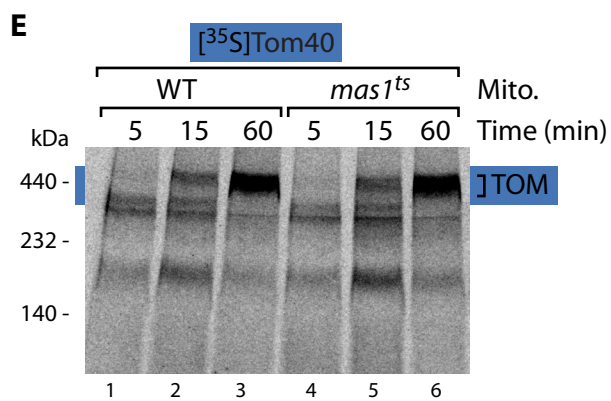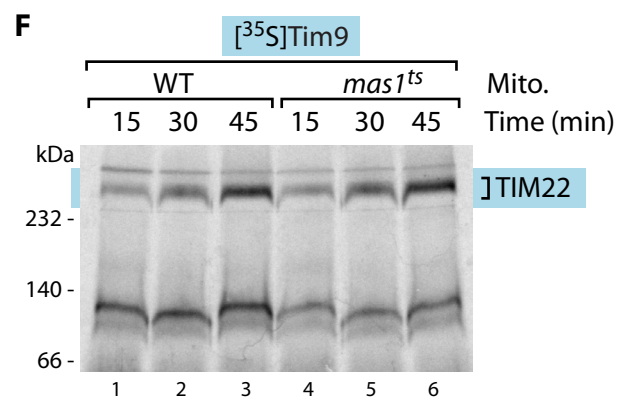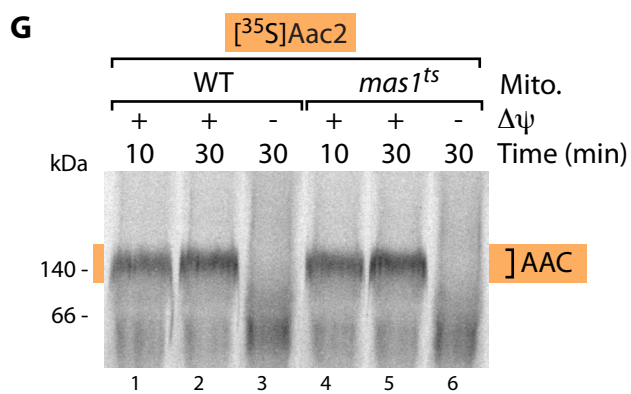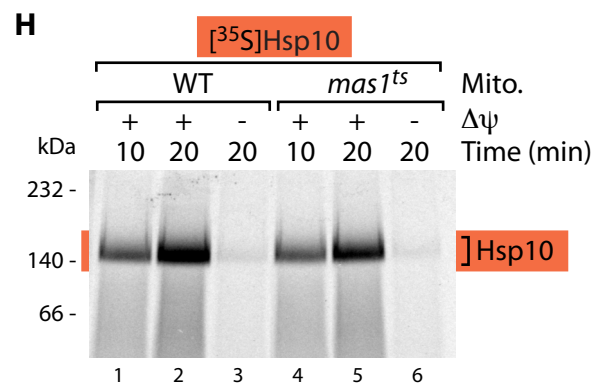

Supplement: S1 Fig — (A) Growth curves of mas1ts and wild-type (WT) yeast cells on respiratory growth medium (YPglycerol) at the permissive temperature 25°C. (B) Growth curves of indicated yeast cells on respiratory growth medium (YPglycerol) at non-permissive temperature 37°C. Dashed lines indicate time points used for import analyses (early mtUPR, 4 and 10 hours, late mtUPR 20 hours growth at elevated temperature). (C)-(H) Import kinetics of indicated radiolabeled precursor proteins into mitochondria isolated from WT or mas1ts cells after growth at 23°C. Where indicated, the membrane potential (Δψ) was dissipated prior to the import reaction. Samples were solubilized in the mild detergent digitonin and analyzed by BN-PAGE and autoradiography. (PDF) [file pgen.1009664.s001.pdf]

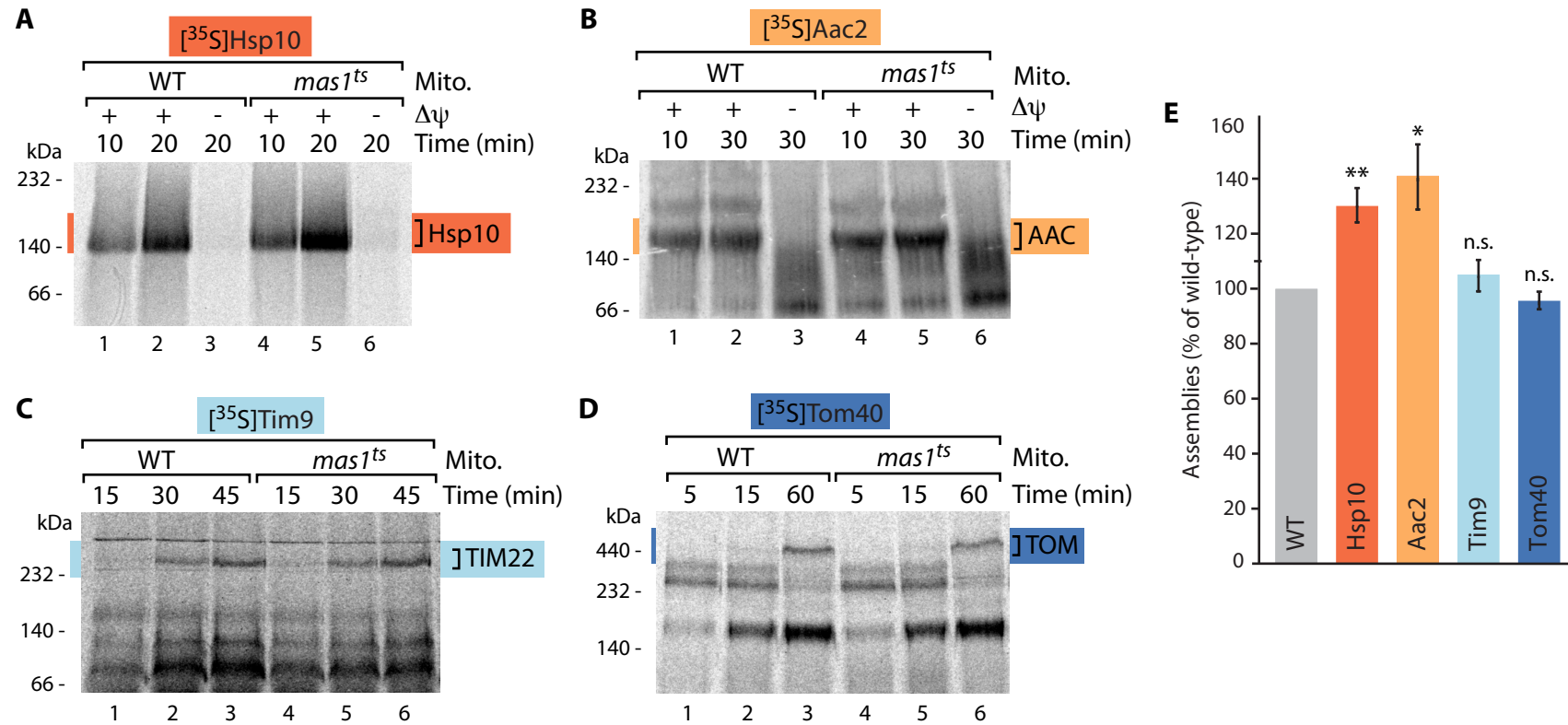

Supplement: S2 Fig — (A)-(D) Analysis of protein import kinetics of indicated radiolabeled precursor proteins into mitochondria isolated from wild-type (WT) or mas1ts cells after induction of mtUPR for 4 hours. Hsp10 is a model substrate for the presequence import pathway and Aac2 for the carrier import pathway into the inner membrane. Where indicated, the membrane potential (Δψ) was dissipated prior to the import reaction. Samples were solubilized with the mild detergent digitonin and analyzed by BN-PAGE and autoradiography. (E) Quantification of longest import time-point displayed in (A)-(D) normalized to WT value. n = 3, data represent means ± SEM. Student’s t-test was used for pairwise comparison. n.s., not significant; *p < 0.05; **p < 0.01. (PDF) [file pgen.1009664.s002.pdf]

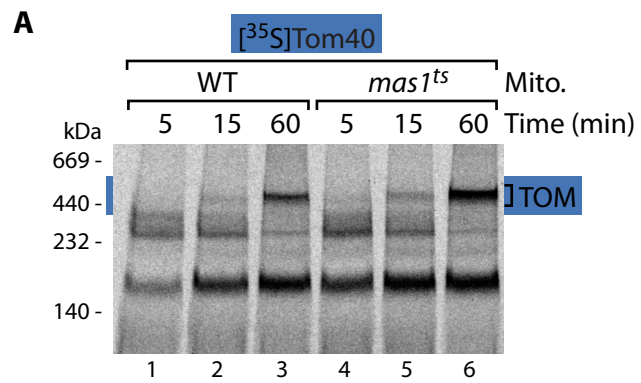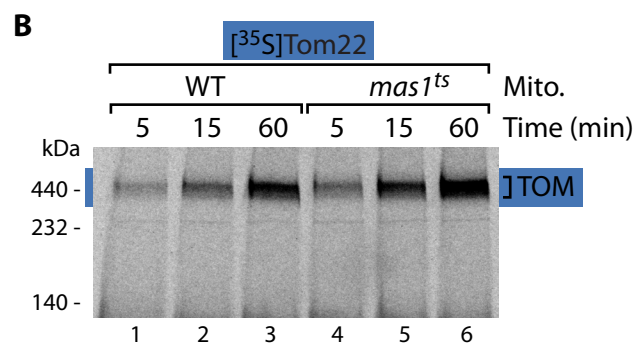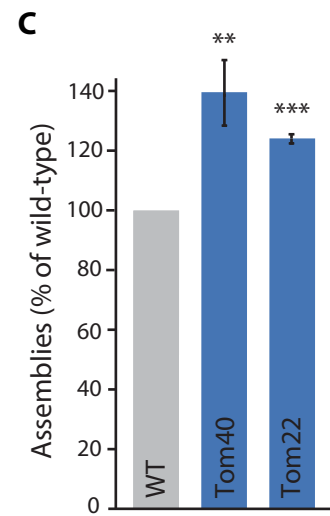

Supplement: S3 Fig — (A) and (B) Blue-native PAGE autoradiography of assembly of Tom40 (A) and Tom22 (B) after import into wild-type (WT) and mas1ts mitochondria isolated after cell growth at non-permissive temperature for 10 hours. (C) Quantification of imports shown in (A) and (B). The longest time point is compared between WT and mas1ts. n = 3, data represent means ± SEM. Student’s t-test was used for comparison. **p < 0.01; ***p < 0.001. (PDF) [file pgen.1009664.s003.pdf]

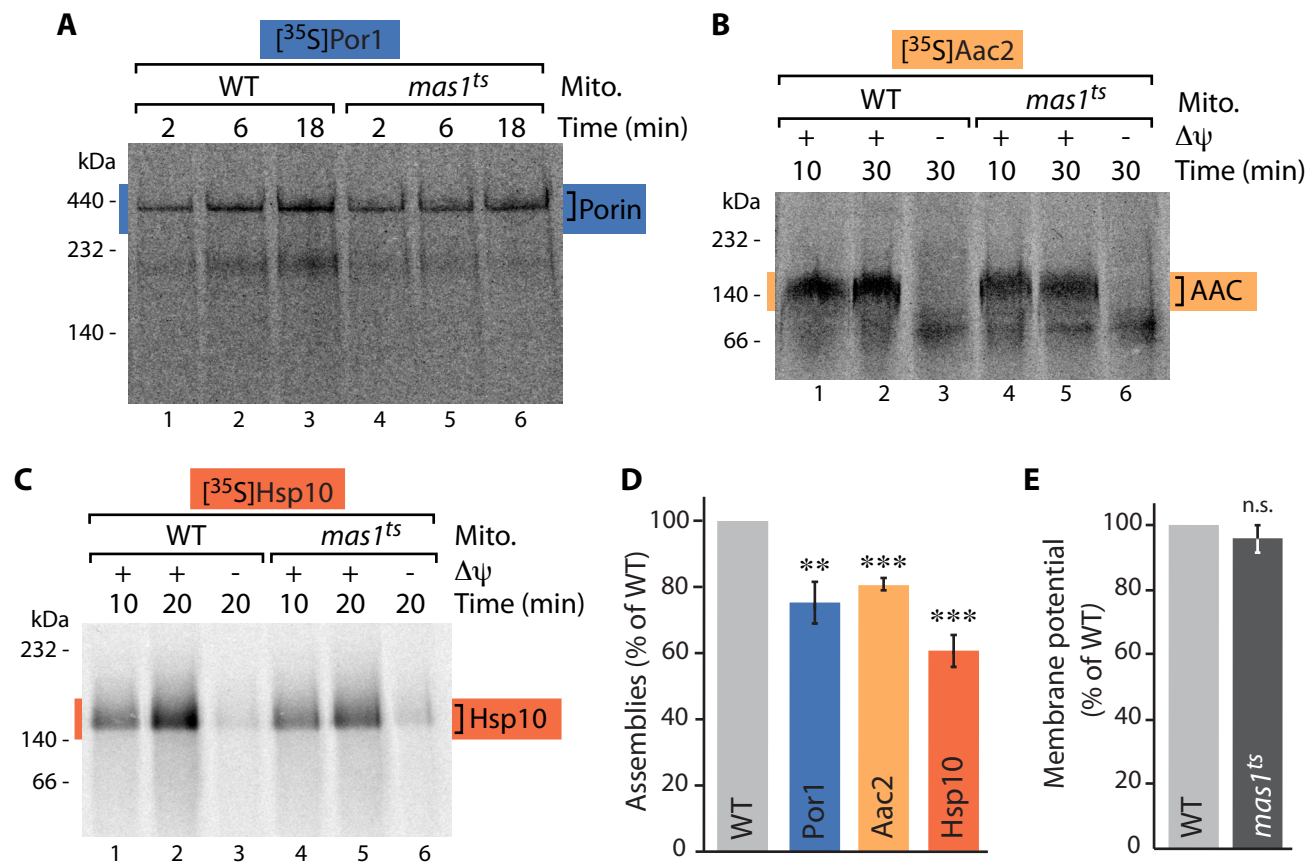

Supplement: S4 Fig — (A)-(C) Kinetic analysis of assembly of indicated radiolabeled precursor proteins into wild-type (WT) and mas1ts mitochondria isolated from cells after growth at 37°C for 20 hours (late mtUPR). Where indicated the membrane potential (Δψ) was dissipated prior to the import reaction. Assembled complexes were analyzed by Blue Native PAGE and autoradiography. (D) Quantification of imports shown in (A)-(C). The longest time point is compared between WT and mas1ts. n = 3, data represent means ± SEM. Student’s t-test was used for comparison. **p < 0.01; ***p < 0.001. (E) Measurement of the membrane potential (Δψ) in WT and mas1ts mitochondria 20 hours after induction of mtUPR. n = 3, data represent mean ± SEM. Student’s t-test was used for comparison. n.s., not significant. (PDF) [file pgen.1009664.s004.pdf]

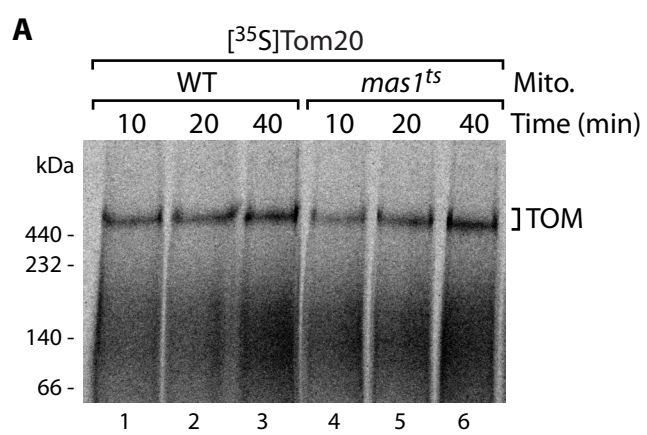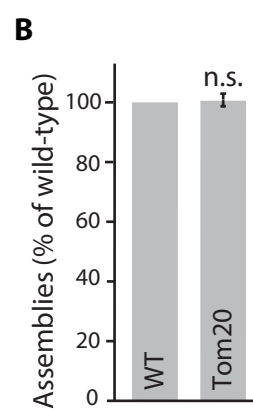

Supplement: S5 Fig — (A) Kinetic analysis of Tom20 protein import, which does not depend on the TOM and SAM complexes for its assembly into the outer membrane. Samples were analyzed by BN-PAGE and autoradiography. (B) Quantification of longest time point of import and assembly of Tom20 shown in (A). n = 3, data represent mean ± SEM. Student’s t-test was used for comparison. n.s., not significant. (PDF) [file pgen.1009664.s005.pdf]

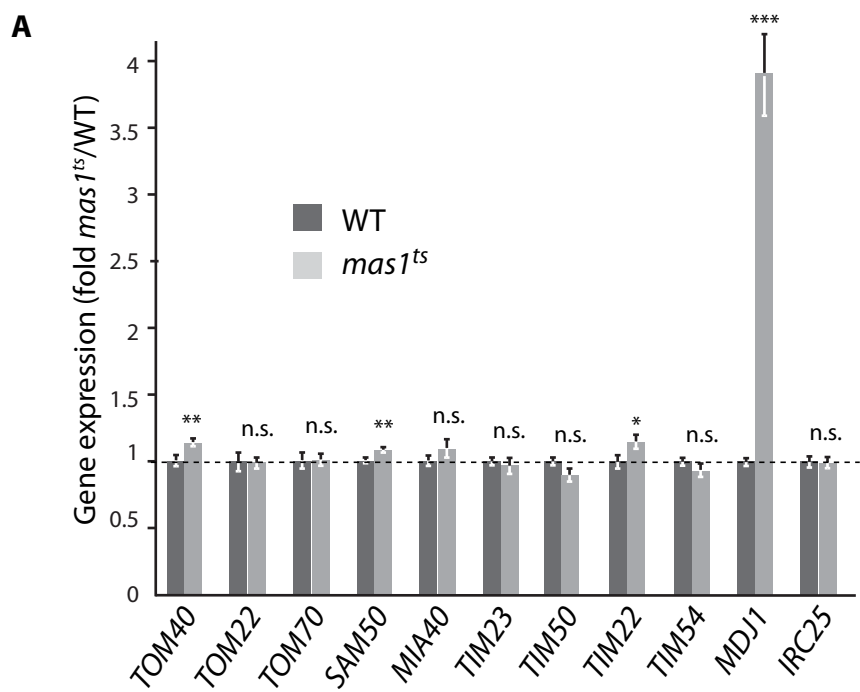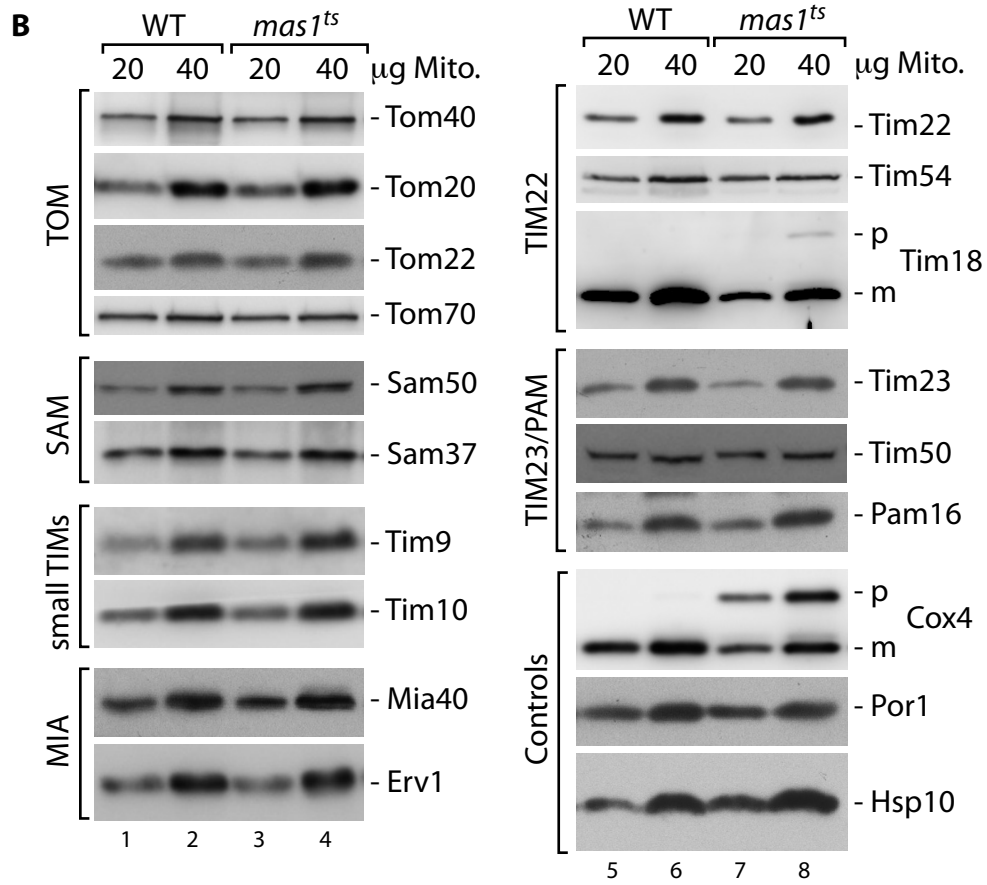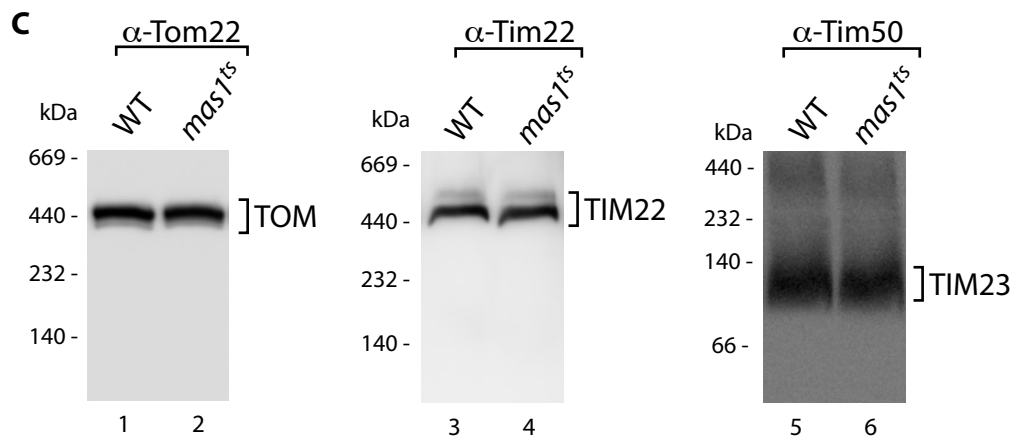

Supplement: S6 Fig — (A) Gene expression analysis of representative genes of the mitochondrial import complexes by RT-qPCR after cell growth for 10 hours at 37°C. MDJ1, encoding for the mitochondrial co-chaperone Mdj1 serves as a positive control for mtUPR induction; IRC25, control. Quantification for n = 6, data represent mean ± SEM. Student’s t-test was used for comparison of WT and mas1ts. n.s., not significant; *p < 0.05; **p < 0.01; ***p < 0.001. (B) Immunoblot analysis of WT and mas1ts mitochondria isolated from cells shifted to non-permissive temperature for 10 hours. For Tim18 and Cox4 the accumulation of their precursor forms due to MPP inhibition is visible. p, precursor; m, mature protein. (C) Analysis of native protein complexes in WT and mas1ts mitochondria by Blue-native PAGE and immunodecoration. Mitochondria were isolated after growth for 10 hours at non-permissive temperature and solubilized with 1% digitonin. (PDF) [file pgen.1009664.s006.pdf]

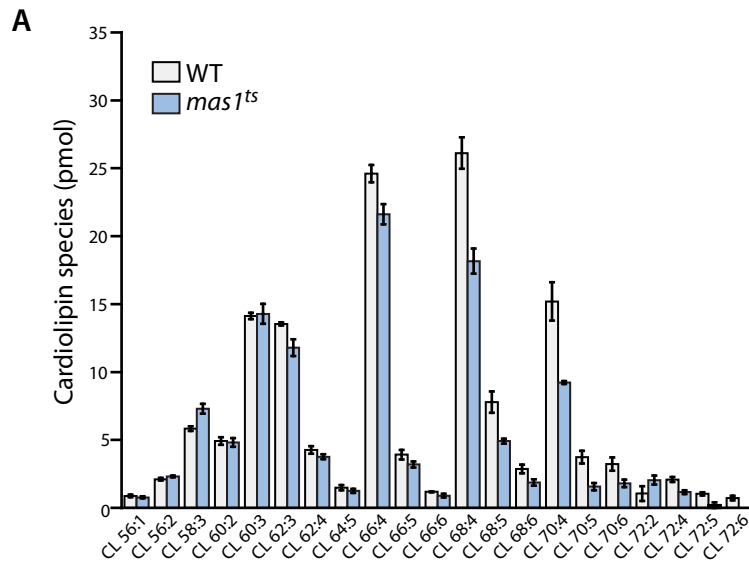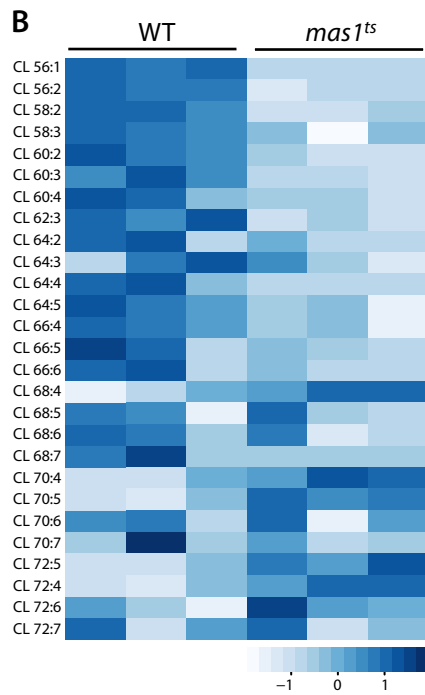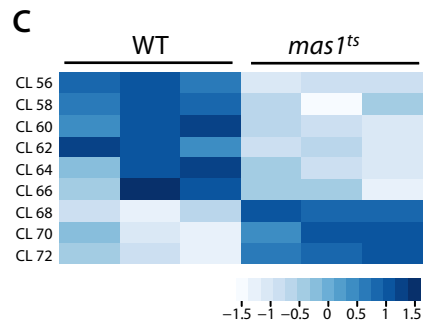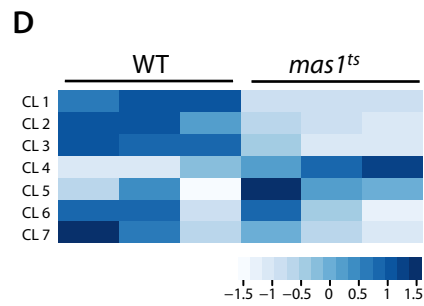

Supplement: S7 Fig — (A) Quantification of indicated cardiolipin (CL) subspecies in wild-type (WT) and mas1ts mitochondria isolated after cell growth at permissive temperature (23°C). n = 3, data represent means ± SEM. (B)-(D) Heatmaps of distribution of different CL subspecies standardized to total CL content per sample in WT and mas1ts mitochondria isolated from cells shifted for 10 hours to non-permissive growth temperature. (C) Analysis of acyl chain length and (D) of number of double bonds. Heatmaps were generated using standardized values in mol% and thus total CL content in each individual sample was set to 100% to represent relative CL species distribution within each sample. Relative changes, scaled and centered for each CL species, are depicted. Shown are three biological replicates for each strain. (PDF) [file pgen.1009664.s007.pdf]

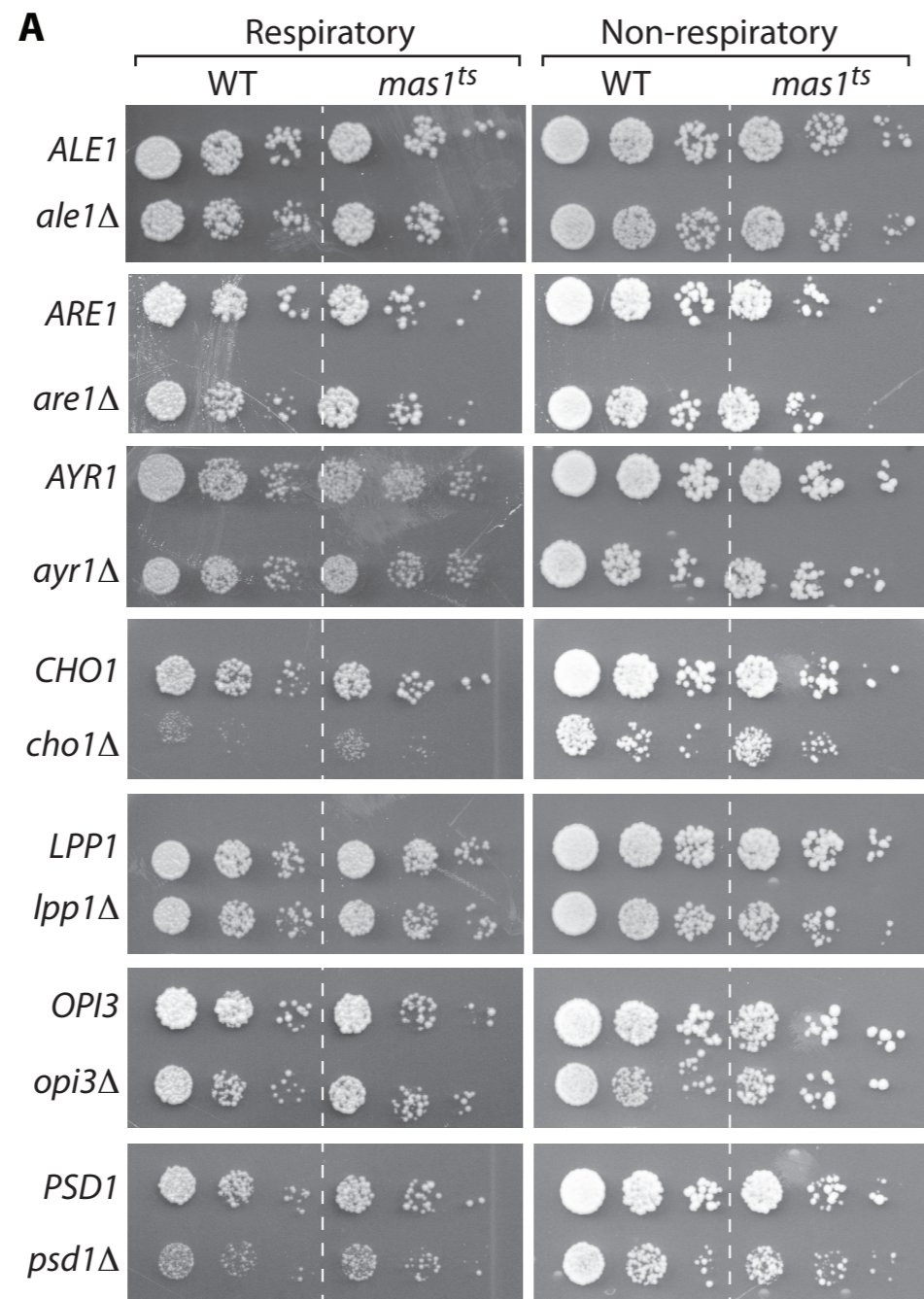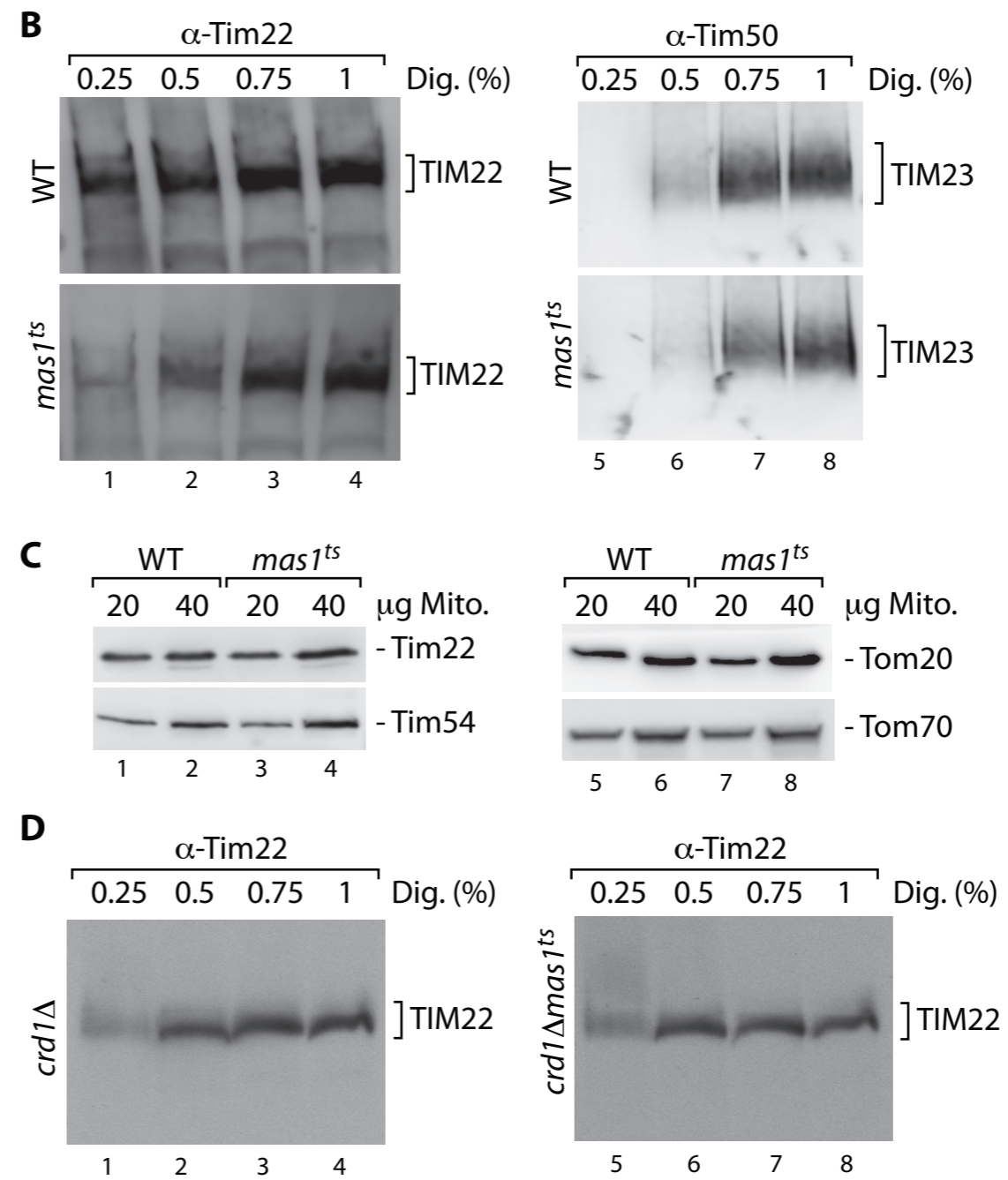

Supplement: S8 Fig — (A) Growth assay to test for synthetic effects of indicated deletions in wild-type (WT) and mas1ts cells. Serial dilutions were tested on respiratory (YPglycerol) and non-respiratory (YPglucose) plates and incubated at 35°C (mild mtUPR induction). (B) Analysis of translocases in WT and mas1ts mitochondria isolated from cells grown for 10 hours at non-permissive temperature. Samples were solubilized with indicated concentrations of digitonin and analyzed by Blue-native PAGE and immunodecoration. TIM22 and TIM23, translocases of the inner mitochondrial membrane. (C) Immunoblot analysis of WT and mas1ts mitochondria isolated from cells shifted to non-permissive temperature for 4 hours. (D) Analysis as in (B) using mitochondria isolated from crd1Δ and crd1Δ mas1ts cells grown under non-permissive temperature for 10 hours. (PDF) [file pgen.1009664.s008.pdf]

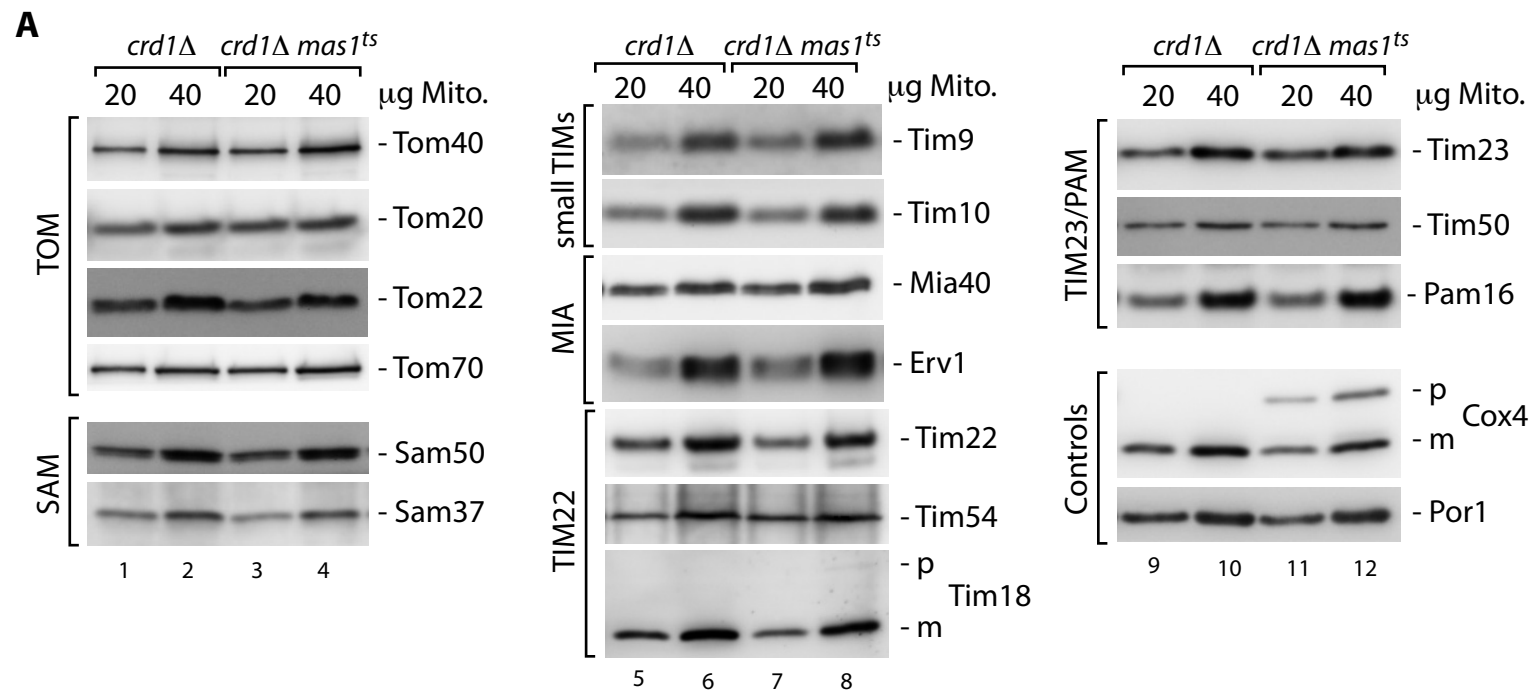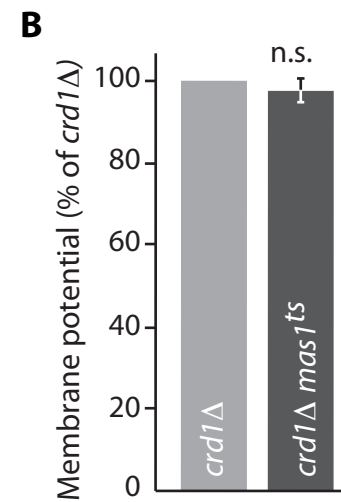

Supplement: S9 Fig — (A) Protein steady state analysis of mitochondria isolated from crd1Δ and crd1Δmas1ts mitochondria after induction of early mtUPR. Samples were analyzed by SDS-PAGE and immunodecoration. p, precursor; m, mature protein. (B) Measurement of the membrane potential (Δψ) in crd1Δ and crd1Δ mas1ts mitochondria after induction of mtUPR. n = 3, data represent mean ± SEM. Student’s t-test was used for comparison. n.s., not significant. (PDF) [file pgen.1009664.s009.pdf]
