# Supplementary material for: Increased mitochondrial protein import and cardiolipin remodelling upon early mtUPR
Source: PLoS Genet. 2021 Jul 2;17(7):e1009664. doi: 10.1371/journal.pgen.1009664 (PMC8282050; doi:10.1371/journal.pgen.1009664)
Supplement: S1 Table — (PDF) [file pgen.1009664.s010.pdf]

S1 Table. List of yeast strains used in this study.

Name	Genotype	Reference
YPH499/Wild-type	YPH499 (WT) MATa, ade2-101, his3-Δ200, leu2-Δ1, ura3-52, trp1 Δ63, lys2-801	47
Wild-type (for *mas1*^{ts} strain)	YPH499 *mas1*::HIS3MX6 pFL39-*MAS1*	22
mas1^{ts}	YPH499 *mas1*::HIS3MX6 pFL39-*mas1*^{R144C}	22
crd1Δ	YPH499 *mas1*::HIS3MX6 pFL39-*MAS1 crd1*::natNT2	This paper
crd1Δ mas1^{ts}	YPH499 *mas1*::HIS3MX6 pFL39-*mas1*^{R144C} *crd1*::natNT2	This paper
taz1Δ	YPH499 *mas1*::HIS3MX6 pFL39-*MAS1 taz1*::natNT2	This paper
taz1Δ mas1^{ts}	YPH499 *mas1*::HIS3MX6 pFL39-*mas1*^{R144C} *taz1*::natNT2	This paper
ale1Δ	YPH499 *mas1*::HIS3MX6 pFL39-*MAS1 ale1*::natNT2	This paper
ale1Δ mas1^{ts}	YPH499 *mas1*::HIS3MX6 pFL39-*mas1*^{R144C} *ale1*::natNT2	This paper
are1Δ	YPH499 *mas1*::HIS3MX6 pFL39-*MAS1 are1*::natNT2	This paper
are1Δ mas1^{ts}	YPH499 *mas1*::HIS3MX6 pFL39-*mas1*^{R144C} *are1*::natNT2	This paper
ayr1Δ	YPH499 *mas1*::HIS3MX6 pFL39-*MAS1 ayr1*::natNT2	This paper
ayr1Δ mas1^{ts}	YPH499 *mas1*::HIS3MX6 pFL39-*mas1*^{R144C} *ayr1*::natNT2	This paper

cho1Δ	YPH499 *mas1*::HIS3MX6 pFL39-*MAS1* *cho1*::natNT2	This paper
cho1Δ mas1^{ts}	YPH499 *mas1*::HIS3MX6 pFL39-*mas1^{R144C}* *cho1*::natNT2	This paper
lpp1Δ	YPH499 *mas1*::HIS3MX6 pFL39-*MAS1* *lpp1*::natNT2	This paper
lpp1Δ mas1^{ts}	YPH499 *mas1*::HIS3MX6 pFL39-*mas1^{R144C}* *lpp1*::natNT2	This paper
opi3Δ	YPH499 *mas1*::HIS3MX6 pFL39-*MAS1* *opi3*::natNT2	This paper
opi3Δ mas1^{ts}	YPH499 *mas1*::HIS3MX6 pFL39-*mas1^{R144C}* *opi3*::natNT2	This paper
psd1Δ	YPH499 *mas1*::HIS3MX6 pFL39-*MAS1* *psd1*::natNT2	This paper
psd1Δ mas1^{ts}	YPH499 *mas1*::HIS3MX6 pFL39-*mas1^{R144C}* *psd1*::natNT2	This paper
