# Supplementary material for: Increased mitochondrial protein import and cardiolipin remodelling upon early mtUPR
Source: PLoS Genet. 2021 Jul 2;17(7):e1009664. doi: 10.1371/journal.pgen.1009664 (PMC8282050; doi:10.1371/journal.pgen.1009664)
Supplement: S2 Table — All antisera were raised in rabbit. (PDF) [file pgen.1009664.s011.pdf]

S2 Table. Primary antibodies used in this study. All antisera were raised in rabbit.

Antigen	Reference	Dilution
Cox4	GR578-4	1:2000
Hsp10	GR4925-3	1:250
Por1	GR3622-2	1:1000
Tom20	GR3225-7	1:5000
Tom40	169-7	1:500
Tom70	GR657-3	1:1000
Tom22	GR3227-3	1:5000
Tim9	GR2013-7	1:250
Tim10	GR2041-7	1:250
Tim18	GR5115-1	1:250
Tim22	GR5113-5	1:250
Tim23	133-14	1:4000
Tim50	GR5185-7	1:500; antiserum raised against the purified mature protein
Tim54	GR5183-7	1:250
Pam16	GR749-5	1:500
Sam37	GR5188-5	1:250; antiserum raised against the purified full-length protein
Sam50	312-15	1:500
Erv1	GR864-2	1:250
Mia40	B315-1	1:1500
Ups1	GR2745-4	1:200
Pgs1	GR1921-5	1:200
