# Supplementary material for: Increased mitochondrial protein import and cardiolipin remodelling upon early mtUPR
Source: PLoS Genet. 2021 Jul 2;17(7):e1009664. doi: 10.1371/journal.pgen.1009664 (PMC8282050; doi:10.1371/journal.pgen.1009664)
Supplement: Attachment — Submitted filename: 110953_1_rebuttal_1917270_qjsbrg.pdf [file pgen.1009664.s012.pdf]

Reviewer #1 (Evidence, reproducibility and clarity (Required)):

Cellular response to defective mitochondrial functions like mtUPR attracts much attention and becomes a hot topic in the cell biology field. In this work, Poveda-Huertes et al. reported interesting results that defective presequence processing due to temperature-sensitive mutation of MPP (the mas1-ts mutation) is associated with enhanced in vitro import of both presequence-containing and presequence-lacking precursor proteins into isolated mitochondria, and with a marginal increase in the cardiolipin (CL) level and decrease in the level of its precursor lipid CDP-DAG in mitochondria.

> We fully agree with the reviewer that mtUPR is a hot topic in the cell biology field and are happy that the reviewer finds our results interesting.

A major problem of this work is, in contrast to the defective import machineries or decreased membrane potential of mitochondria, that the effects of defective presequence cleavage are not clear. In wild-type mitochondria, cleaved presequence peptides are depleted by peptide degradation in the matrix. However, in mitochondria with defective presequence cleavage, presequence-containing precursor proteins will be accumulated in the matrix, which may cause toxicity to the cell, but its mechanism is not clear; perhaps, accumulation of presequence-containing precursor proteins in the matrix may perturb the membrane structures of the inner membrane, trap matrix chaperones like mtHsp70 and mtHsp60, and so on. Thus, it is not appropriate to call such an unclear outcome of the defective presequence cleavage "mtUPR".

> We have analyzed these points raised by the reviewer in our previous publication, in which we in detail describe the toxic effect of defective presequence processing on the cell and characterize the mtUPR triggered by this defect (Poveda-Huertes et al., Mol. Cell 2020). The *mas1^{ts}* mutation used in the present manuscript and our previous study is based on a patient mutation we identified and characterized in our publication Vögtle et al., American Journal of Human Genetics (2018). We show that defects in MPP processing result in accumulation of non-processed precursor proteins in the mitochondrial matrix. These precursors rapidly form aggregates that cannot be degraded by the mitochondrial proteolytic system. Furthermore, we showed that this precursor aggregation in the matrix results in a fast transcriptional response (already after 2 hours) that triggers an increase in mitochondrial proteases and chaperones, which is a classic characteristic of mitochondrial unfolded protein responses (mtUPR) across species (see also comment to reviewer 3 below). Based on these results we termed the response an *early* mtUPR (Poveda-Huertes et al., Mol. Cell 2020). We have added some of the data from this previous publication below and hope that this clarifies the points of the reviewer (see also comment of reviewer 2 on our previously published results, which provided the basis of this manuscript). Taken together, the outcome of the mutation is well characterized and an excellent model to investigate the different steps and characteristics of mtUPR.

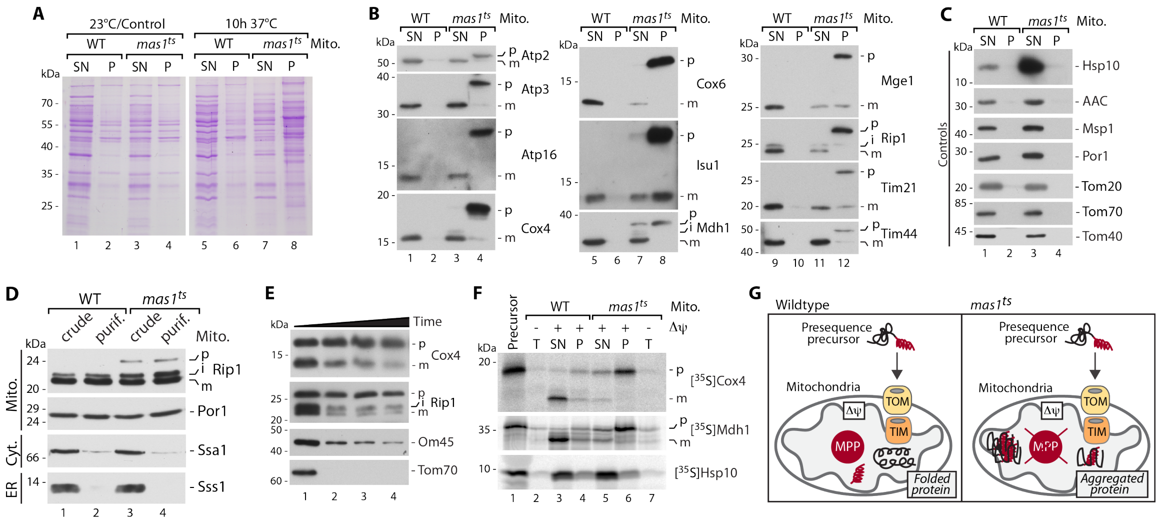

Compendium of Figure 1 and S1 of Poveda-Huertes et al., Mol. Cell 2020

(A) Coomassie-stained gels from SDS-PAGE analyses of wildtype (WT) and *mas1*^{ts} mitochondria isolated from cells grown under respiratory conditions and separated into soluble (SN, supernatant) and aggregated (P, pellet) protein fractions.

(B) Mitochondria isolated from wild-type (WT) and *mas1*^{ts} cells grown for 10 hours at 37°C were separated into soluble (SN, supernatant) and aggregated (P, pellet) protein fractions and analyzed by SDS-PAGE and immunodecoration. All non-processed precursor proteins were detected in the aggregated fraction, while processed, mature proteins were recovered as soluble proteins.

(C) Analysis as in (B) of non-processed proteins. The strong increase in the chaperone Hsp10 in the *mas1*^{ts} samples is a first indication of a mitochondrial unfolded protein response.

(D) Comparison of non-processed precursor proteins accumulating in crude and highly purified mitochondria reveal that aggregates are within the organelle. Ssa1, cytosolic marker; Sss1, ER marker.

(E) Degradation assay reveals that precursors cannot be degraded as efficiently as mature proteins.

(F) Newly imported precursor proteins that cannot undergo processing rapidly aggregate directly upon import.

(G) Cartoon of import into WT and *mas1*^{ts} mitochondria with subsequent aggregation of non-processed precursor proteins.

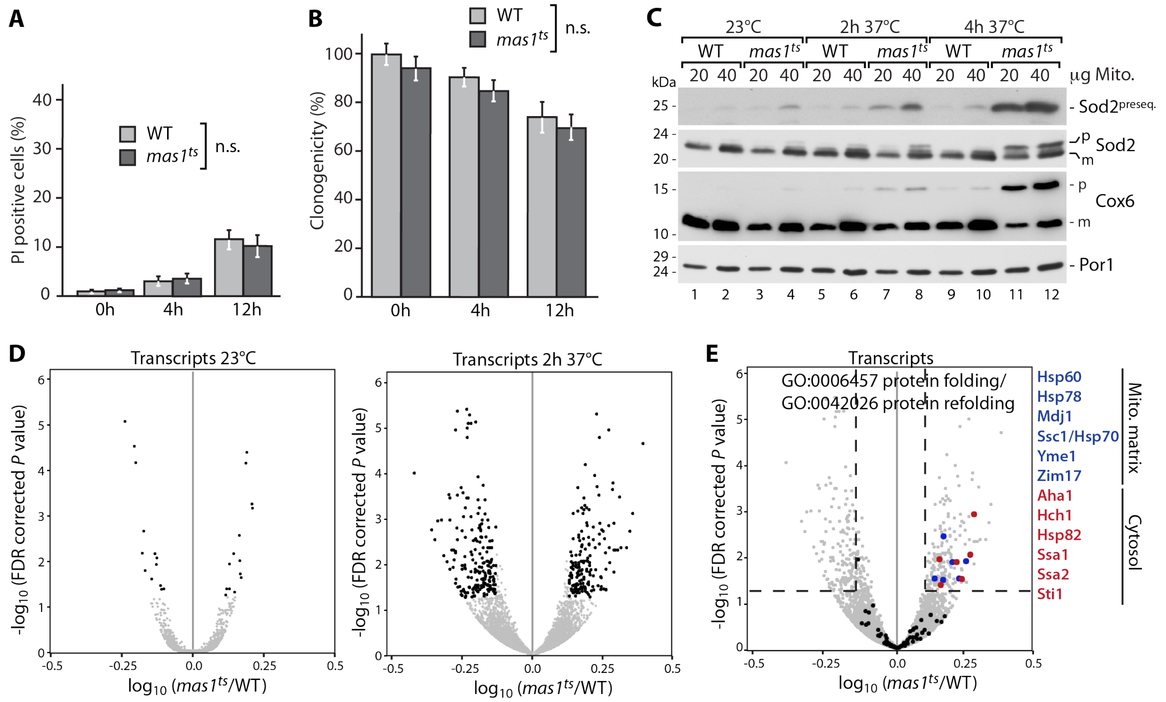

Figure 2 of Poveda-Huertes et al., Mol. Cell 2020

(A) Cell death determined by flow cytometric quantification of propidium iodide (PI) staining indicative of loss of plasma membrane integrity of wildtype (WT) and *mas1*^{ts} cells after shift to 37°C for indicated time. n = 12, data represent means +/- SEM; n.s., not significant. *mas1*^{ts} cells do not show increased cell death upon MPP inactivation.

(B) Determination of clonogenicity via survival plating of WT and *mas1*^{ts} cells after shift to 37°C for the indicated time. n = 6, data represent means +/- SEM.

(C) Immunoblot analysis of WT and *mas1*^{ts} mitochondria isolated from strains shifted for indicated times to non-permissive temperature. Sod2$^{preseq.}$, antibody generated against presequence peptide of Sod2. Accumulation of precursor proteins can be detected already after two hours of MPP inactivation.

(D and E) Distribution of transcripts quantified by RNA-seq in WT and *mas1*^{ts} cells. Displayed are Benjamini-Hochberg adjusted P-values. GO terms provided by Saccharomyces genome database. FDR, false discovery rate. *mas1*^{ts} cells trigger a rapid transcriptional response after MPP inactivation and up-regulate mitochondrial chaperones.

Besides, at this moment, there is no clear causative relationship between the enhanced in vitro import of several precursor proteins and changes in some lipid levels including the CL level. Therefore, this work is too premature to draw any conclusion or model to speculate the role of CL in overcoming the toxicity of the mas1-ts mutation or defective presequence processing.

> There seems to be a misunderstanding: We do not claim or propose a model that changes in cardiolipin are overcoming the toxicity of the *mas1*^{ts} mutation. We propose a

model, in which as part of the mtUPR the cells adapt the mitochondrial lipid composition by increasing their signature lipid cardiolipin to stabilize the mitochondrial import translocases to enhance protein biogenesis. This model is supported by our data showing that import into all four mitochondrial subcompartments increases upon mtUPR (Figure 1) and that CL levels increase under these conditions (Figure 2). Only upon deletion of the cardiolipin synthase enzyme Crd1, this increased import rates are abrogated (Figure 3), thereby connecting changes in CL with the import effect upon mtUPR. We provide now further support for our model by deletion of *TAZ1* (Taz1 is required for CL remodelling; Schlame, BBA 2013), which also impacts on growth in *mas1*^{ts} like the deletion of *CRD1* demonstrating that cardiolipin remodelling is required upon mtUPR (see Rebuttal Figures R10 and R11, reviewer 3). Furthermore, we deleted several other lipid biosynthesis enzymes and deletions in no other lipid biosynthesis pathways had an effect on the *mas1*^{ts} cells (Rebuttal Figure R12, reviewer 3), clearly showing a specific role for cardiolipin upon mtUPR.
We also have now further data supporting the link of lipid changes and mtUPR: We assessed the extraction of the inner membrane translocases from the lipid bilayer using different concentrations of the mild detergent Digitonin. Both inner membrane translocases TIM22 and TIM23 show a different profile in WT and *mas1*^{ts}, in which higher amounts of digitonin are required for extraction of the translocases from their lipid environment (Rebuttal Figure R1). This indicates that the lipid composition surrounding the translocases changes upon mtUPR, fully supporting our model that changes in CL stabilize the import machineries to support import upon mtUPR.

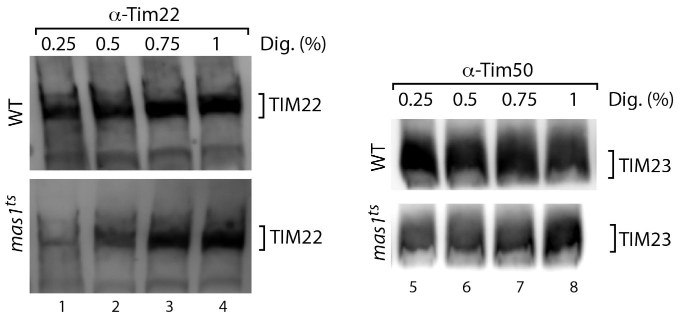

Rebuttal Figure R1. Assessment of translocase extraction from the mitochondrial lipid bilayer. Isolated mitochondria from wild-type (WT) and *mas1*^{ts} cells grown for 10 hours at 37°C were solubilized with different digitonin concentrations. While the inner membrane translocases were already efficiently extracted with low digitonin concentrations in wild-type mitochondria, in the *mas1*^{ts} mutant higher amounts of detergent concentrations were necessary. This indicates that the lipid environment embedding the translocases in the IM is modulated upon mtUPR.

The followings are additional concerns for this manuscript. 1)In vitro protein import into isolated mitochondria is a powerful tool to analyze the mitochondrial functions. However, the properties of mitochondrial protein import are not always the same between in vitro and in vivo. Therefore the authors should test if similar enhanced

import is observed for mitochondrial proteins in vivo by assessing the levels of transiently expressed mitochondrial proteins etc.

> The use of intact isolated mitochondria in combination with radiolabeled precursor proteins is the standard in the research field of mitochondrial protein biogenesis. Our current knowledge how mitochondria import proteins is based on these in organello assays. The advantage of the in organello system is that the import competence of the translocation machineries can be directly assessed. As we focus here on the role of the protein import machinery upon mtUPR we think that in organello experiments give us the most direct and uncompromised read-out. Only by this assay the import process can be largely uncoupled from potentially interfering other cellular events in extra-mitochondrial compartments. Furthermore, we have indication that protein import is also fully active in vivo upon mtUPR as the protein steady state levels, of e.g. the mitochondrial chaperone Hsp10 increase upon mtUPR (see Figure S3B, below). We have shown previously that this increased protein abundance of Hsp10 is not caused by a change in Hsp10 turn-over (Poveda-Huertes et al., Mol. Cell 2020, see Figure below), pointing into the direction that an increased import rate of Hsp10 is causative for the increased protein level.

We have now also used radiolabeled precursors translated from yeast cytosolic ribosomes (as requested by reviewer 3) and also in this homologous system we see increased import upon mtUPR fully supporting our model that mitochondrial protein import is not compromised but on the contrary boosted upon early mtUPR (see Rebuttal Figure R13, Reviewer 3).

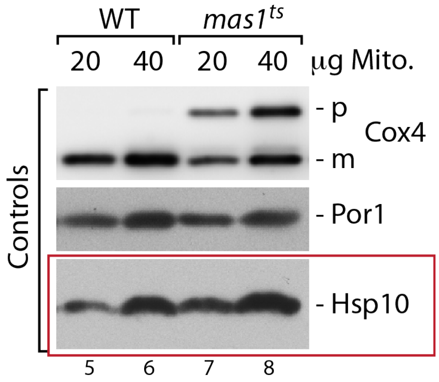

Figure S3B. The mitochondrial chaperone Hsp10 is strongly increasing upon mtUPR. Newly synthesized Hsp10 is efficiently imported from the cytosol into the mitochondrial matrix under stress conditions demonstrating that protein import is not compromised upon mtUPR in vivo.

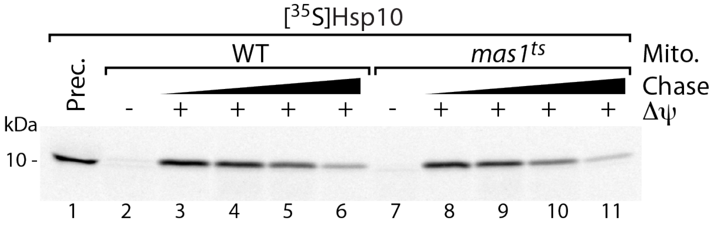

Figure from Poveda-Huertes et al., Mol. Cell 2020 Degradation assay showing that turn-over of mitochondrial Hsp10 is not changed upon mtUPR.

2)It is known that a decrease in the CL level impairs mitochondrial protein import, but this does not mean that increase in the CL level increases the efficiency of mitochondria protein import. If this is the case, the CL level in wild-type mitochondria is not optimized for the most efficient import of mitochondrial proteins, which is a very provoking hypothesis that needs to be experimentally tested.

> Dynamic changes in the lipid composition are a mean for the cell to adapt to changing demands, e.g. upon heat shock or metabolic changes. Also, under stress conditions it is conceivable that changes in lipids are a mean to protect the cell from deleterious effects. However, the reverse conclusion that import is not optimized under standard conditions is not accurate. The cell adjusts its needs under each condition and this is always a trade-off between different and sometimes opposing factors. Here, we show that upon mtUPR cells re-adjust their CL levels, which is crucial for the cell to support import of newly translated mitochondrial chaperones and proteases. This increased import is made possible by the stabilization of the import translocases, which we now show by titration of digitonin (see Rebuttal Figure R1).
The cell is probably maintaining crucial functions at an optimum for each particular scenario in the complicated interplay of all global cellular processes. As a consequence, these different functions cannot be kept at their maximum capacity at all times. If all biological processes would always be kept at their maximum capacity, the cell would not have the means and possibilities to regulate and adapt upon changing cellular demands. One example is the mitochondrial ribosome: Mutations in the accuracy center by insertion of a point mutation in the Mrps12 subunit results in hyperaccurate translation. However, this is not beneficial, but on the contrary results in severe respiratory growth defects due to a decreased translation rate. Similarly, introduction of hypoaccurate mutations also showed a growth defect, this time caused by the resulting mistranslation of proteins (Suhm et al., Cell Metabolism 2018). Also the main import gate into mitochondria TOM was shown to be regulated by phosphorylation in specific phases of the cell cycle adapting import of specific precursor proteins to cellular demands (Harbauer et al., Science 2014). These examples show that the activity of biological processes is a trade-off between a plethora of factors and functions. That the mitochondrial import machinery has the possibility to adapt upon changes in cellular states, e.g. to stabilize the import machineries upon mitochondrial stress upon which import is crucial to rescue mitochondrial defects, seems therefore very reasonable.

3)It is important to reveal the mechanism of the increased CL level. For this purpose, the authors should test the levels of Crd1, Gep4, Pgs1, and Tam41. Indeed, the reason for the CL level change may be complicated since the level of PE decreased and the level of PS increased with time in mas1-ts mutant mitochondria (Fig. 2A).

> The changes in PE and PS indicated by the reviewer are occurring with the same dynamic also in the wild-type control and are therefore not specific to mtUPR induction.

We have also analyzed several additional deletions of proteins involved in lipid metabolism and only detected growth defects upon deletion of *CRD1* and *TAZ1* in *mas1*^{ts}, which are both key enzymes in cardiolipin biosynthesis (see Rebuttal Figures R10, R11 and R12, reviewer 3). Furthermore, only the changes in CL levels show a clearly different dynamic when comparing the *mas1*^{ts} with the wild-type control. To investigate the mechanism behind these adaptation in CL levels, we have analyzed the steady state levels of proteins involved in cardiolipin biosynthesis and for which specific antisera were available as suggested by the reviewer. We have identified an increase in Ups1 that plays a crucial role in CL metabolism by transporting the precursor phosphatidic acid from the outer to the inner mitochondrial membrane, where CL lipid synthesis takes place (Li et al., BBA 2007). We also detected an increase in the protein Pgs1 (phosphatidylglycerolphosphate synthase) that catalyzes the synthesis of phosphatidylglycerolphosphate from CDP-diacylglycerol (CDP-DAG), which is actually the first and rate-limiting step of CL biosynthesis (Rebuttal Figure R2). The increase in Ups1 and Pgs1 likely are the mechanistic foundation for the changes in CL upon mtUPR, fully supporting our model.

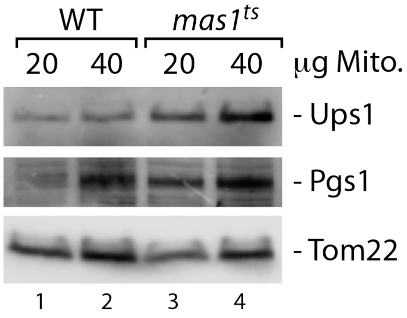

Rebuttal Figure R2. Steady state protein levels of isolated mitochondria from wild-type (WT) and *mas1*^{ts} cells grown for 10h at 37°C. Protein levels of Ups1 and Pgs1 increase upon mtUPR, while the control Tom22 does not.

Reviewer #1 (Significance (Required)):

This work is potentially interesting in terms of the cellular response to the defective mitochondiral proteostasis or mtUPR. However, the work is too preliminary stage and requires more experiments to draw an entire picture of the observed results.

> We think that our additional experiments together with the foundation of our mtUPR model, which was published in Molecular Cell and is acknowledged by the second reviewer, is moving our work far beyond a preliminary stage. We also have provided further data fully supporting our model that changes in CL are resulting in a boost of protein import upon mtUPR, which is of interest as acknowledged by reviewer 1.

Reviewer #2 (Evidence, reproducibility and clarity (Required)):

The authors in this study focussed on the analysis of earlry stages of mtUPR, using a genetic mutation of the Mas1 gene that creates a temperature-sensitive phenotype. They argue that early stages of mtUPR do not involve any substantial changes in inner membrane potential and characterized by an increase in all major protein import pathways. Decreased protein import is observed on the other hand only for later stages of mtUPR indicative of sustained stress. The concept they propose is very intriguing and the potential interest of this work is significant. I agree that, so far, essentially all studies of mtUPR utilized very severe stressors (CCCP treatment of mitochondria for example), which are very harsh and probably do not allow to study what happens in the early stages of mtUPR where the stress imposed is milder. Their previous work on this aspect of mtUPR clarified this idea and in fact supported very well this distinction of early vs late stages of mtUPR. The main point of the work here is that the early stages of mtUPR are linked to a remodeling of cardiolipin (which is enriched in the mitochondrial inner membrane), and that this remodeling boosts the protein import processes.

> We are very happy about this positive comment on our manuscript and the appreciation of our previous work (Poveda-Huertes et al., 2020), in which we identified the early mtUPR using the *mas1*^{ts} mutant and which is the ideal model for the analysis presented in the current manuscript.

Main points:

1.The effect of cardiolipin remodeling should be primarily affecting import pathways for which the inner membrane is involved. How can they explain the effects on the Tim9 pathway which does not require an electrochemical potential and no association with the inner membrane?

> While CL is with approximately 16% an abundant lipid component in the mitochondrial inner membrane, mass spectrometry based analyses of highly purified outer mitochondrial membranes revealed that CL is also found with 1.6% in the outer membrane (Vögtle et al., Journal of Cell Biology 2015; Gebert et al., Current Biology 2009). Notably, even very low abundant lipids can have a strong effect on protein biogenesis: We have previously demonstrated that phosphatidic acid, which is present in the outer membrane with only 0.4%, is crucial for import and assembly of the fusion protein Ugo1. Decreasing the low PA amount further directly correlates with a decrease in Ugo1 assembly (Vögtle et al., JCB 2015). Furthermore, it was reported that the translocases of the outer membrane, among them the TOM complex, critically depend on CL for their functionality (Gebert et al., Current Biology 2009). Therefore, the import of all mitochondrial precursor proteins that use the TOM complex, which encompasses virtually all precursors, can be affected by changes in CL levels, among them Tim9 as shown in our study.

2.The authors talk about remodelling of CL upon mtUPR but they don't show the CL subspecies distribution in WT and Mas1-ts mutant cells before mtUPR in figure 2D. Since the total levels in uninduced Mas1-ts cells seem to be lower than in WT, and that increasing the temperature has an effect in the WT too, they should analyze the profile in uninduced cells in order to have a better idea of what it is going on. Also, Figure 2E shows a great variability in the levels of each CL subspecies in the three biological replicates, and how they have normalized this is not clear. In Figure 2G, they show that eliminating Crd1 in the WT also has a harsh effect on the growth of the WT cells at 35ºC (not as much as in the masts background, but still very noticeable).

> We have performed the analysis of CL subspecies distribution under permissive growth conditions as requested by the reviewer (Rebuttal Figure R3). Levels of certain CL subspecies are indeed a bit lower in the *mas1*^{ts} mutant under permissive conditions. The decrease of CL might be indicative of slight differences between WT and *mas1*^{ts} already under permissive growth conditions, which is also reflected by few transcriptional changes (see Figure 2 from Poveda-Huertes et al., 2020, reviewer 1). The comparison of the CL subspecies at permissive temperature and after 4 hours of induction now clearly demonstrates the dynamic change in CL upon mtUPR.
In Fig. 2A-D, the abundance of phospholipids and cardiolipin species is shown in absolute values (pmol of the respective lipid detected in each sample). As shown in Fig. 2B, compromised Mas1 function results in an overall increase in cardiolipin content. To visualise the relative abundance of the different cardiolipin subspecies within these samples, a heat map was generated (Figure 2E). Here, values have been standardized to each sample and are thus depicted as mol%. Thus, the total cardiolipin content in each sample is set to 100% and relative distribution of all cardiolipin subspecies is shown.
It was published previously that deletion of *CRD1* results in a mild growth defect at elevated temperature (38°C) in wild-type cells (e.g. Gebert et al., Current Biology 2009). The synthetic growth defect upon deletion of *CRD1* in the *mas1*^{ts} background is more severe pointing to a genetic interaction of MPP function and cardiolipin biosynthesis. We have now in addition analyzed the effect of deletion of *TAZ1* as requested by reviewer 3, which shows the same growth defect as seen upon deletion of *CRD1* further underlining the specific effect and requirement for CL upon mtUPR (Rebuttal Figures R10 and R11, reviewer 3).

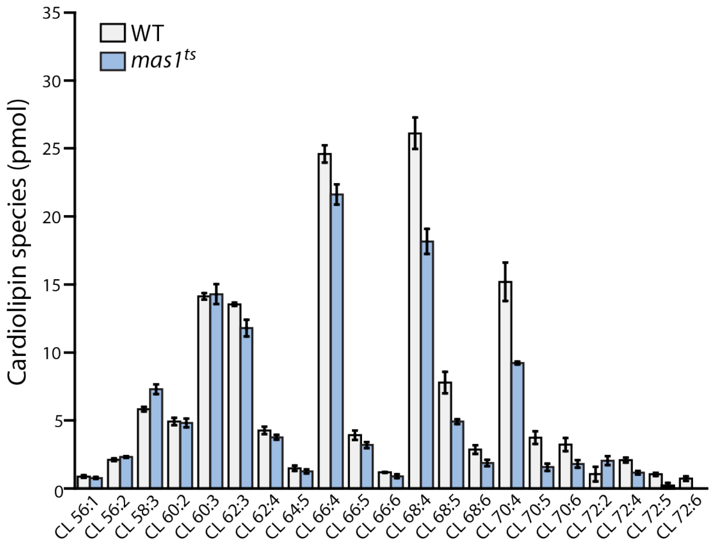

Rebuttal Figure R3. Quantification of indicated CL subspecies in WT and *mas1*^{ts} mitochondria isolated after growth at permissive temperature. n=3, data represent means ± SEM.

3.In Fig 2C and D: It is not explained why at 2 and 4 hours there is such an increase in the CDP-DAG precursor also in the WT, that does not then translate in the increase in the levels of CL. Also, in the Mas1-ts mutant the 4-fold increase in the precursor at 2 hours that disappears at 4 hours translates in a modest increase in the total amounts of CL at 4 hours shown in Figure 2B. Also, are the units right? There are around 120 pmol total CL but only 0.5 pmol CDP-DAG so if this is right, it is questionable how much this can contribute to the formation of the total pool of CL. Can the authors please clarify this point?

> CDP-DAG is a precursor for several other phospholipids besides cardiolipin including phosphatidyl choline (PC) and phosphatidyl inositol (PI). The increase in CDP-DAG in the wild-type could therefore be due to general lipid modulation upon the applied heat shock. Furthermore, CDP-DAG is a lipid precursor that is only transiently present and rapidly further modified to generate the indicated phospholipid species. It is short lived and of low abundance, and does not accumulate in mitochondria under normal conditions (Osman et al., JCB 2011), which is why it is only detected in low amounts in our lipidomics analyses.

4.In Fig3: the effect on AAC2 is not surprising as it is known that import and assembly of carriers proteins (and stability of them in membrane complexes) is affected by CL. It is not clear here whether the effect is truly on import or on assembly in the membrane. The authors can check whether proper insertion is affected if they analyze the imported AAC2 in mitoplasts with protease treatment which would normally give a smaller 2-3 kDa species if insertion is working well, but would result in further degradation if the AAC2 species is not properly inserted.

> The reviewer is absolutely right that AAC depends on the lipid composition during its biogenesis, but that also mature AAC requires several cardiolipin molecules for stable

assembly. The assay suggested by the reviewer is an excellent idea and we will perform this experiment for the revised version of the manuscript. We have so far only assessed standard import on SDS for Hsp10 and can also detect a decrease in import here fully supporting our hypothesis that changes in cardiolipin are required to maintain import upon mtUPR (Rebuttal Figure R4).

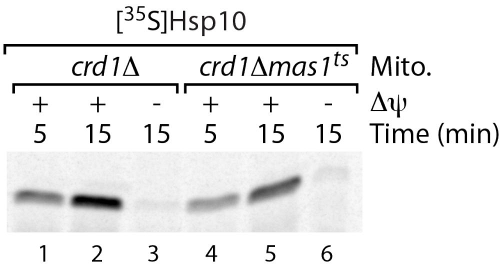

Rebuttal Figure R4. Import of radiolabeled Hsp10 precursor into mitochondria isolated from *crd1Δ* and *crd1Δmas1^ts* cells after growth for 10 hours at 37°C. All samples were treated with Proteinase K to remove non-imported precursor proteins.

Minor points:

1.The proposal that CL increase and subspecies remodeling likely stabilizes the mitochondrial import complexes thereby increasing protein import capacity into the organelle under stress conditions is an intriguing model. However, the authors show no changes in the steady-state levels of the import complexes upon stress in Figure supplement 3 or in the absence of cardiolipin remodelling in Figure supplement 5. In line with this, they say that there are no changes in the TOM complex upon stress because Tom20 import/assembly does not change (Figure supplement 1), although in this same figure the TOM complex increases if you look at the import of Tom40 and Tom22 at 60 minutes in Panels A, B and C. Can the authors explain this? There is increased import/assembly but no differences in steady-state levels of Tom40 and Tom22 (Figure supplement 3). Is it because there is also increased degradation? Have the authors looked into this?

> We will assess the degradation of TOM subunits by in vivo degradation assays for the revised version of this manuscript. We already have novel data on the extraction of translocase complexes from the lipid bilayer that indicate that the lipid surrounding of the translocases is modulated upon mtUPR (see Rebuttal Figure R1, review 1). As a further explanation for the observation that the TOM complex is not increasing we would like to point out that the radiolabeled precursors used in our analyses were chosen as model substrates for the different mitochondrial import routes. They were used as a mean to assess the activity of the import machinery in general. While from these results it is conceivable to deduce that protein import into mitochondria is generally enhanced upon mtUPR, this does not mean that more Tom subunits will be imported in vivo (only if their transcription/translation increases, more proteins could be imported. However, transcription of e.g. Tom subunits does not increase upon mtUPR (see Figure 3A)). We expect that rather the proteins, which are also increased on a transcriptional level, e.g. chaperones and proteases, will benefit from the increased

import capacity and result in increased protein levels (as shown for Hsp10 in Figure S3B). We will include this point in the revised manuscript.

2.The times after induction of mtUPR in which they have done the import assays should be explained. In material and methods it is indicated that 10 hours after stress were used as indicative for 'early' effects, and 20 hours for the 'late' effects. However, the CL remodelling was assessed after 2 and 4 hours. There is a discrepancy here which I don't understand. In fact this does not allow an accurate interpretation of the data because things could change between 4 and 10 hours. In this respect, I think an interesting point of the paper is the difference in the import between the 'early mtUPR' and the 'late mtUPR' shown in Figure supplement 4. This is an important point of the paper and I recommend this should be included as a main figure. Also, there should be quantifications and statistical analysis like in Figures 1 and 3. They should also assess what happens to cardiolipin in this late mtUPR if they want to make the direct relation between 'cardiolipin remodelling' and import efficiency in response to the different phases of mtUPR. Is it possible that at late stages of mtUPR CL persists and some of it is rearranged to the outer membrane where it could in fact act as a cell death triggering signal? Is there a 'threshold' of CL remodeling above which mitochondria are committed to mitophagy and apoptosis?

> The reviewer is right that we measured lipid changes after 2 and 4 hours of mtUPR induction and performed import experiments after 10 hours. The lipidomics analyses were performed after 2 and 4 hours to be able to detect the initial start and the dynamics of the changes in CL. CL is turned over much slower than other phospholipids so that once CL is assembled it remains inert to degradation (Xu and Schlame, 2014). Due to its long half-life changes in CL should indeed persist over a longer period of time as proposed by the reviewer. We have already obtained additional biochemical data that lipid changes impact on the import translocases after 10 hours of mtUPR: Digitonin titration to assess the extractability of the inner membrane translocases TIM22 and TIM23 revealed that while very low amounts of digitonin are sufficient to extract the complexes in controls, an increasing digitonin concentration is required upon mtUPR (see Rebuttal Figure R1 above, comment to reviewer 1). This indicates that the lipid environment around the translocases is indeed changing upon mtUPR and that this changes are maintained over a longer period of time. We will in addition assess CL levels upon prolonged mtUPR as suggested by the reviewer in the revised version of the manuscript.

We will include the analysis of import competence upon late mtUPR as a main figure as suggested by the reviewer. We have also performed quantifications and statistical analyses as requested and they are fully supporting our hypothesis that protein import into mitochondria only declines upon late stages in mtUPR (Rebuttal Figure R5).

It is an intriguing idea that the increase in CL upon prolonged mtUPR might serve as a signal for apoptosis, due to its exposure in the outer leaflet of the outer mitochondrial membrane. We have now assessed survival of *mas1*^{ts} cells and could not detect an increase in cell death upon mtUPR induction (Rebuttal Figure R7, Reviewer 3).

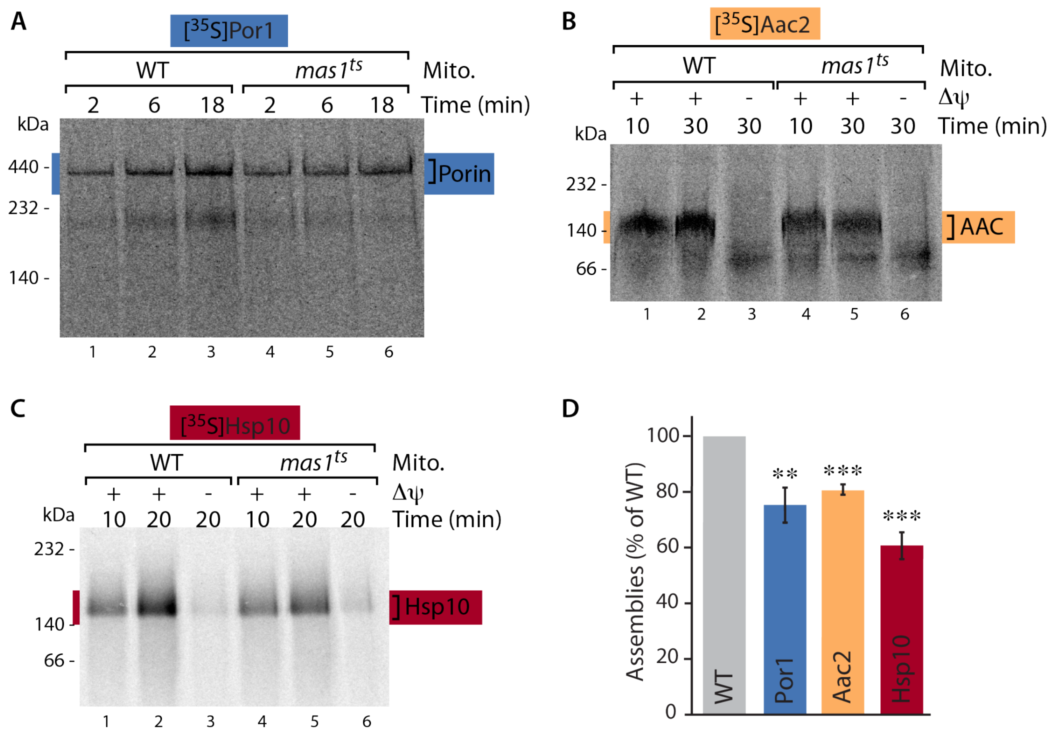

Rebuttal Figure R5. Import assays of indicated precursor proteins into mitochondria isolated from wild-type and *mas1*^{ts} cells grown for 20 hours at 37°C. Upon this late mtUPR stage import into mitochondria is significantly reduced (quantification of last import time point in D). Quantification for n=3, data represent means +/- SEM.

3.The authors use the term mitochondrial dysfunction in several parts of the text (for example p. 5 and 7), but it is not clear they mean by that in the context of their study. Given that membrane potential, protein import and mtDNA expression are unaffected, what kind of dysfunction do they refer to? Please explain this point.

> We apologize for this inaccuracy. We will replace the term mitochondrial dysfunction in the text and will describe specifically the particular dysfunction, e.g. MPP dysfunction.

Reviewer #2 (Significance (Required)):

The potential advance of the paper is the definition of early stages of mtUPR and a potential mechanism (CL remodeling) that accompanies this. Also, the increase rather than the, commonly assumed, decrease is an unexpected new result that looks at the mtUPR pathways under a completely different angle.

The audience are researchers interested in mitochondria biogenesis, intracellular sorting

and mechanisms of protein homeostasis in the cell.

My expertise is on mitochondria biogenesis, protein targeting and mitochondria biology in general.

> We thank the reviewer for this significance assessment of our study. We are especially glad about the comment that our work looks at mtUPR under a completely different angle.

Reviewer #3 (Evidence, reproducibility and clarity (Required)):

The mitochondrial unfolded protein response (mtUPR) is one of several mechanisms that have evolved to counteract mitochondrial dysfunction and maintain the health of the organism. This adaptive transcriptional response was studied mainly in C. elegans and based on findings in this system this process was suggested to be regulated by the mitochondrial import efficiency of the transcription factor ATFS-1. Upon mitochondrial dysfunction, import of ATFS-1 into mitochondria is reduced, allowing it to be trafficked to the nucleus where it promotes the expression of genes encoding mainly chaperones and proteases that promote survival and recovery of the organelle.
The impairment of genes involved in diverse aspects of mitochondrial function, addition of reagents like paraquat or doxycycline, and the accumulation of misfolded protein in the matrix of the organelle were reported to induce mtUPR. It was suggested that these impairments activate UPRmt because the import efficiency of the mitochondrial network is reduced.

In the present contribution the authors propose an alternative model where protein import is not inhibited, but rather actually enhanced upon mtUPR. This enhancement is linked to up-regulation in the synthesis and modelling of cardiolipin.

However, as I wrote in details below, I think that the experimental set-up does not allow the authors to obtain results that can support these claims.

> The experimental set-up is based on our previous publication on mtUPR (Poveda-Huertes et al., Mol. Cell 2020), in which we have analyzed all points raised by the reviewer (see first major issue below) in detail. Taking this validation of our model into account (which is also highlighted by reviewer 2), we are convinced, that we can use our experimental set-up to perform experiments and obtain results on mtUPR and that these results fully support our claims.

Major issues:

1.The authors claim that the transcriptional reprogramming, which is associated with the mtUPR starts already after 2 hrs at 37{degree sign}C. Nevertheless, they define as an

"early stage" mitochondria that were isolated 10 hrs after the shift to the non-permissive temperature. Thus, using mitochondria that were isolated after 10 hrs cannot be considered as an "early" stage but in contrast might already harbor elevated levels of proteases and chaperones.
To obtain real information on the status of the isolated mitochondria, the authors should follow systematically (for example, after 1, 2, 5, 10, 15, and 20 hrs) the steady-levels of mitochondrial chaperones (like Hsp70, Hsp60/10) and proteases (Yme1, Yta10/12, Pim1, and others). A hint for elevated levels of chaperones are the high levels of the co-chaperone Mdj1 (Fig. Suppl. 3A), which the authors failed to comment.
The results of Fig. 2C demonstrate that already two hrs time difference (between 2 and 4 hrs) can cause major deviations in the outcome.

> We have analyzed the transcriptional changes upon mtUPR in the *mas1*^{ts} cells in our previous publication (Poveda-Huertes et al., Mol Cell 2020). We showed that the transcriptional response is rapid and that transcripts of mitochondrial chaperones and proteases increase. We also show that these results in an increase in mitochondrial protein steady state levels of e.g. the heat shock protein Hsp10 (which is also used as a positive control in the present manuscript, see Figure S3B). Similarly, the increased transcripts of *MDJ1* served as a positive control in the qPCR analyses of Figure S3A (we apologize for not making this more clear in the current manuscript, we will add this in the figure legend of the revised version). Therefore, we are employing a well characterized model for early mtUPR. The reversible and mild induction of mtUPR in the *mas1*^{ts} cells results in measurable kinetic changes that do allow for the first time the dissection of early and lates stages. The *mas1*^{ts} model furthermore enables us to investigate the big conundrum in mtUPR research how increased transcription/translation of mitochondrial chaperones and proteases can result in increased steady state levels of these proteins within the organelle if mitochondrial protein import is blocked (which is the current mtUPR model). Our model allows dissection of the temporal stages in mtUPR and reveals that upon early time points in which transcription of mitochondrial chaperones and proteases is strongly increased, also the import capacity of the organelle is modulated by changes in the signature lipid CL. This allows import of the newly synthesized chaperones and proteases. We have added some of the key findings of our previous publication below.

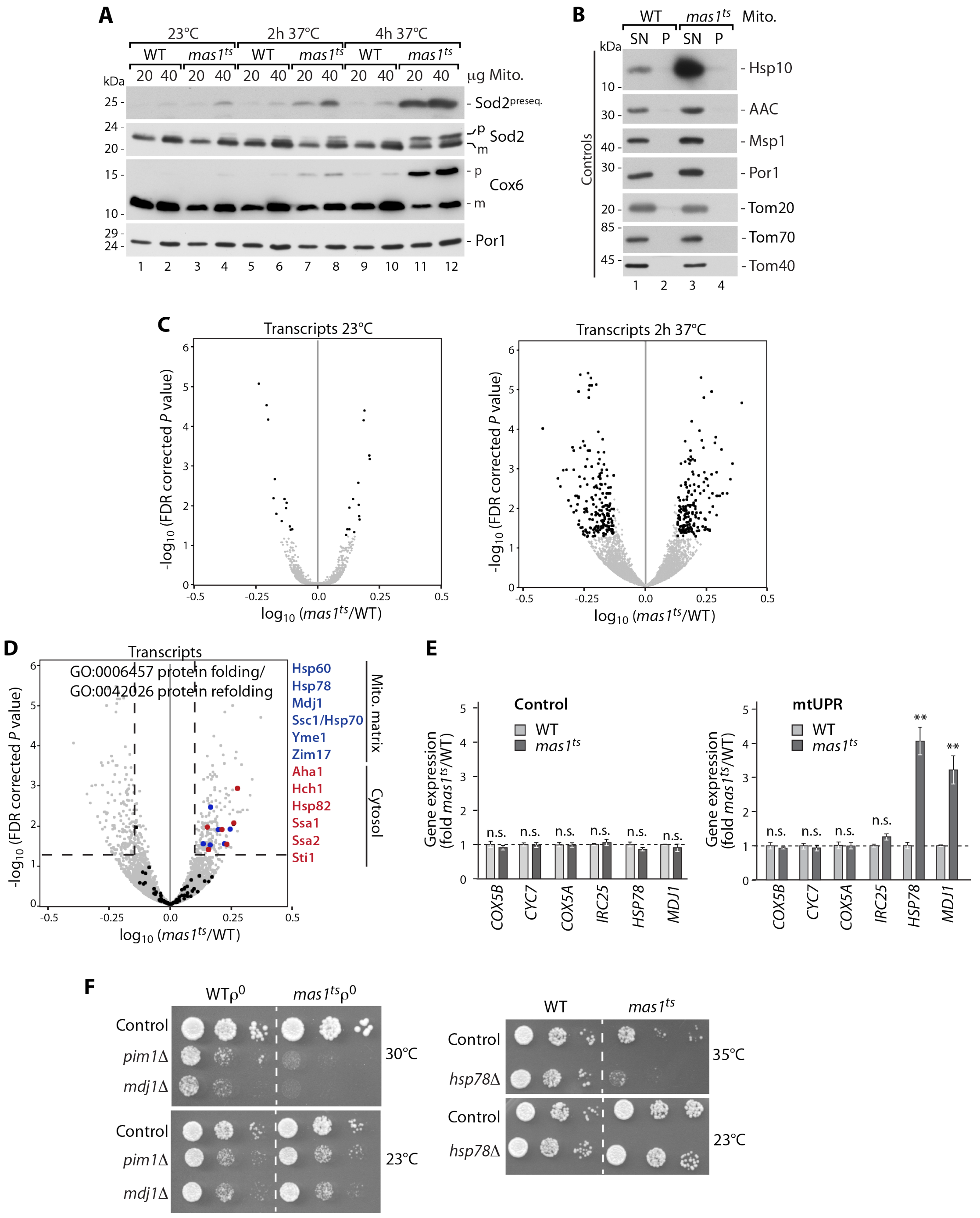
A
23°C
2h 37°C
4h 37°C
WT
mas1ts
μg Mito.
kDa
Sod2preseq.
Sod2
Cox6
Por1
B
SN
P
Mito.
Controls
Hsp10
AAC
Msp1
Tom20
Tom70
Tom40
C
Transcripts 23°C
Transcripts 2h 37°C
-log10 (FDR corrected P value)
log10 (mas1ts/WT)
D
Transcripts
GO:0006457 protein folding/
GO:0042026 protein refolding
Hsp60
Hsp78
Mdj1
Ssc1/Hsp70
Yme1
Zim17
Aha1
Hch1
Hsp82
Ssa1
Ssa2
Sti1
Mito. matrix
Cytosol
E
Control
mtUPR
Gene expression (fold mas1ts/WT)
COX5B
CYC7
COX5A
IRC25
HSP78
MDJ1
n.s.
**
F
WTρ0
mas1tsρ0
pim1Δ
mdj1Δ
hsp78Δ
30°C
23°C
35°C

Compendium of Figure 2 and S2 from Poveda-Huertes et al., Mol. Cell 2020.

(A) Immunoblot analysis of WT and *mas1*^{ts} mitochondria isolated from strains shifted for indicated times to non-permissive temperature. Sod2$^{preseq.}$, antibody generated against presequence peptide of Sod2. Accumulation of precursor proteins can be detected already after two hours of MPP inactivation.

(B) Mitochondria isolated from wild-type (WT) and *mas1*^{ts} cells grown for 10 hours at 37°C were separated into soluble (SN, supernatant) and aggregated (P, pellet) protein fractions and analyzed by SDS-PAGE and immunodecoration. The strong increase in the chaperone Hsp10 in the *mas1*^{ts} samples is an indication of a mitochondrial unfolded protein response.

(C and D) Distribution of transcripts quantified by RNA-seq in WT and *mas1*^{ts} cells. Displayed are Benjamini-Hochberg adjusted P-values. GO terms provided by Saccharomyces genome database. FDR, false discovery rate. *mas1*^{ts} cells trigger a rapid transcriptional response after MPP inactivation and up-regulate mitochondrial chaperones.

(E) Gene expression analysis of indicated genes by RTqPCR after growth at 23°C (control) or after 2 hours at 37°C (mtUPR). *HSP78* and *MDJ1* are encoding mitochondrial chaperones. Quantification for n=3, data represent means +/- SEM.

(F) Growth tests to assess synthetic effects of indicated mutant strains on *mas1*^{ts} and wild-type cells. In case of *pim1Δ* and *mdj1Δ* rho^0 background was used due to the loss of mtDNA by the deletion.

2.The authors observed import reduction after shifting the cells for 20 hrs at the non-permissive temperature. What is the general status of cells after such long time at 37{degree sign}C? Is their mitochondrial morphology normal? How about the membrane potential of such isolated organelles? Do they duplicate in the time between 10 to 20 hrs? Without this information it is not clear if mas1ts cells after 20 hrs represent a mtUPR "late stage" or simply cells in bad shape.

> We have assessed key parameters of cellular and mitochondrial fitness after growth of *mas1*^{ts} cells at non-permissive temperature for 20 hours as requested by the reviewer. Growth curves of wild-type and *mas1*^{ts} cells at 37°C show that the *mas1*^{ts} mutant still divides under our stress conditions (Rebuttal Figure R6, left panel). We have compared our mtUPR model with some of the previously used triggers of mtUPR (Rebuttal Figure R6, right panel), in which induction of mtUPR immediately eliminates all cellular growth. We also assessed cellular death in *mas1*^{ts} cells over 24 hours and could not detect increased cell death upon mtUPR compared to wildtype (Rebuttal Figure R7).
Similarly, the membrane potential is largely unaffected after 20 hours, while addition of menadione to induce oxidative stress (a classic mtUPR trigger often used in other studies, reviewed in Vögtle, FEBS J 2020) completely depletes this key parameter of mitochondrial fitness (Rebuttal Figure R8).
Taken together, these data clearly show that the *mas1*^{ts} cells are not "in bad shape" and that the induction of mtUPR using our reversible *mas1*^{ts} model is providing an excellent model to analyze different stages of mtUPR.

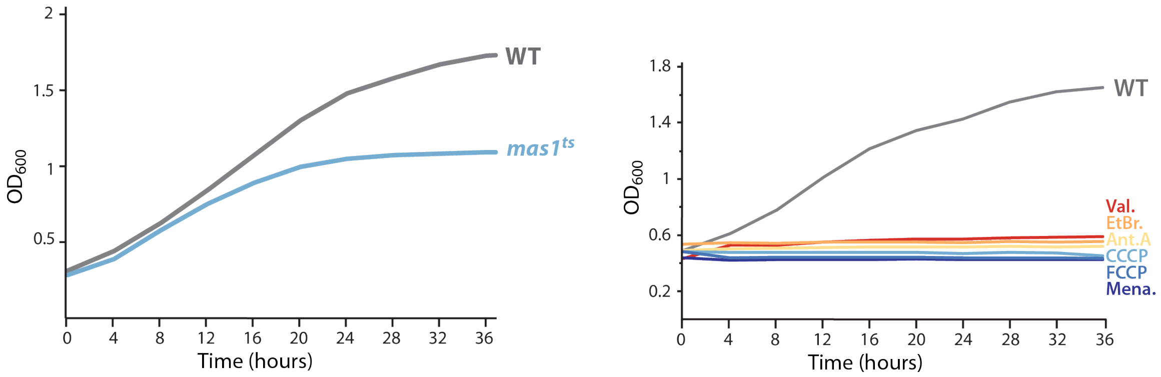

Rebuttal Figure R6. Growth curve of wild-type (WT) and *mas1*[ts] cells on non-fermentable carbon source at non-permissive temperature (37°C) revealing that the *mas1*[ts] mutant is dividing after 20 hours of induction (left panel). Growth curves in the right panel display previously used triggers for induction of mtUPR in the literature that all immediately inhibit cell division (Val., valinomycine 2 μM; EtBr., Ethidium bromide treatment 60 μg/mL; Ant. A, Antimycin A 1 μM; Mena, menadione; CCCP, Carbonyl cyanide m-chlorophenyl hydrazone 10 μM; FCCP, Carbonyl Cyanide. P-(Trifluoromethoxy) Phenylhydrazone 10 μM).

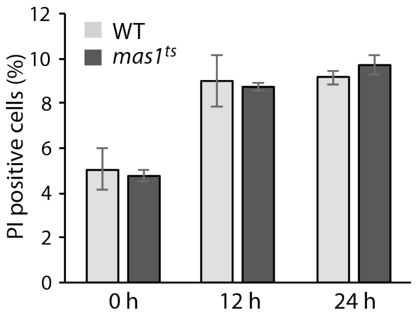

Rebuttal Figure R7. Analysis for PI positive cells (indicative of cell death) in wild-type and *mas1*[ts] cells demonstrates that the *mas1*[ts] cells do not show increased cell death after 24 hours of growth at non-permissive temperature.

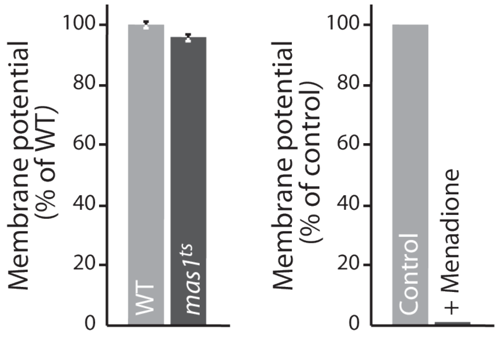

Rebuttal Figure R8. Membrane potential measurement using mitochondria isolated after growth at 37°C for 20 hours (left panel). The membrane potential in the *mas1*[ts] mutant is comparable to wild-type. In contrast, mitochondria isolated after treatment with 0.3 mM menadione do not have a measurable membrane potential anymore.

3.The changes in the phospholipids were determined after 2 or 4 hrs at 37{degree sign}C. Hence, it is hard to correlate these findings with the results regarding protein import which were determined after 10 or 20 hrs. Currently, a causative link between

phospholipids change (measured after 4 hrs) and enhanced import (monitored at 10 hrs) is not possible. To establish a connection between altered lipid composition and changes in import efficiencies, the authors should analyze both aspects (lipidomics and protein import) after the same time periods at the non-permissive temperature.

> We show in Figure 3 that deletion of the cardiolipin synthase Crd1 impacts strongly on import competence in the *mas1^{ts}* cells thereby linking the enhanced protein import upon mtUPR to CL modulation. CL is long-lived and we have now also additional experiments showing that lipid changes persist after 10 hours as the inner membrane translocases TIM22 and TIM23 are showing different lipid extraction profiles upon mtUPR compared to control (Rebuttal Figure R1, reviewer 1). Furthermore, we show in addition that also deletion of *TAZ1* impacts on growth of *mas1^{ts}* cells fully supporting our hypothesis that CL remodelling plays a role upon mtUPR (Rebuttal Figures R10 and R11).

4.Fig. 2F & G: The mas1ts cells are sick to start with. Thus, it is not surprising that the deletion of CRD1 on this background resulted in even further phenotypes. However, how specific are these observations? The authors should delete other genes that are relevant to lipid metabolism, for example PSD1 and UPS1/2, and test whether they observe similar or other results. Along the same line, Taz1 is important for the modelling of CL species. To study the relevance of CL modelling, the authors should create a mas1ts strain deleted for TAZ1.

> We performed growth curves of wild-type and *mas1^{ts}* cells at 25°C that reveal no difference in growth between the two strains. Thus the *mas1^{ts}* are as healthy as wild-type cells and only display a growth defect upon induction of MPP dysfunction by an increased growth temperature. Furthermore, we have now deleted several other genes involved in lipid biosynthesis as requested by the reviewer (Rebuttal Figure R12) and only mutations in CL biosynthesis (*CRD1*, *TAZ1*) result in a stronger growth defect of the *mas1^{ts}* mutant clearly revealing that the effect is specific to CL (Rebuttal Figures R10 and R11).

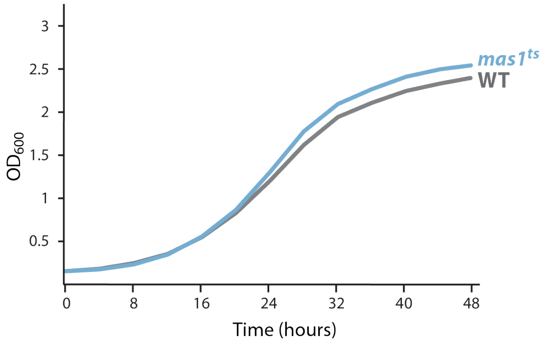

Rebuttal Figure R9. Growth curve of wild-type (WT) and *mas1^{ts}* cells on non-fermentable carbon source at permissive temperature (25°C) revealing that the *mas1^{ts}* mutant is growing comparable to wildtype cells.

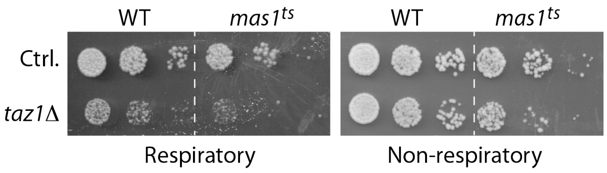

Rebuttal Figure R10. Growth test of wild-type and *mas1*^{ts} cells with additional deletion of *TAZ1* at 35°C. Cardiolipin remodelling is required for growth of *mas1*^{ts} cells upon induction of mtUPR.

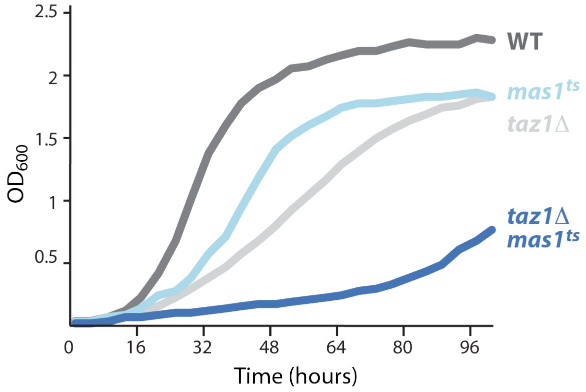

Rebuttal Figure R11. Growth curves of indicated strains at non-permissive temperature (35°C) confirming that cardiolipin remodelling by Taz1 is required for growth of *mas1*^{ts} cells.

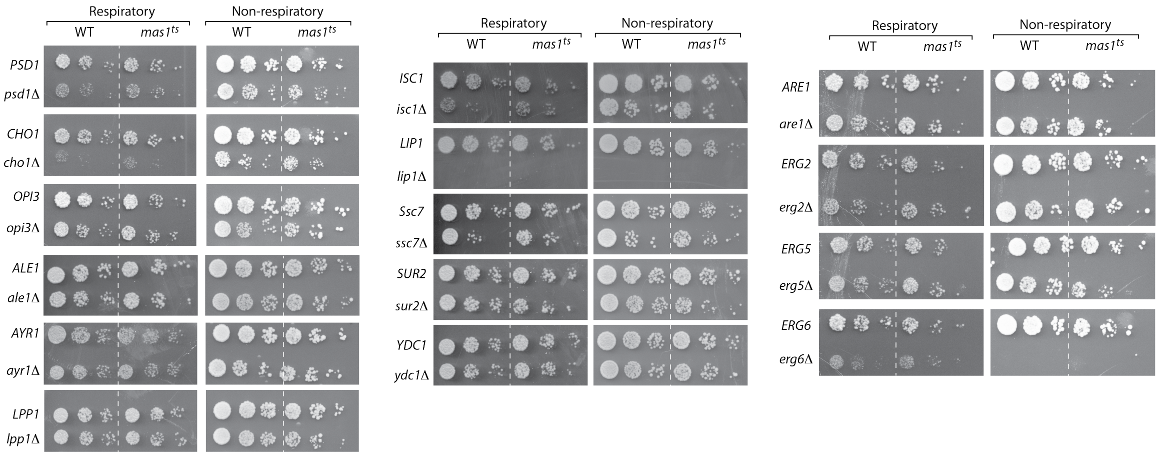

Rebuttal Figure R12. Analysis of growths of indicated yeast strains. Deletions in several lipid synthesis pathways do not impact on growth of *mas1*^{ts} cells.

5.An important part of the mtUPR are cytosolic events. In their in organello import set-up, the authors use rabbit retic. lysate as a cell-free synthesis system. This situation eliminates any effect of yeast cytosolic factors, which might play an important role in authentic protein import. Thus, I wonder whether a homogenous system (isolated organelles and cytosolic fraction from the same organism) would result in similar results.

> We will repeat key experiments with radiolabeled precursors synthesized using yeast

translation extracts as requested by the reviewer. We have already set-up and established a protocol for radiolabelling of precursors using yeast cytosolic ribosomes and performed already an initial analysis of Por1 import, which shows the same result as before when using the rabbit reticulocyte lysate (Rebuttal Figure R13).

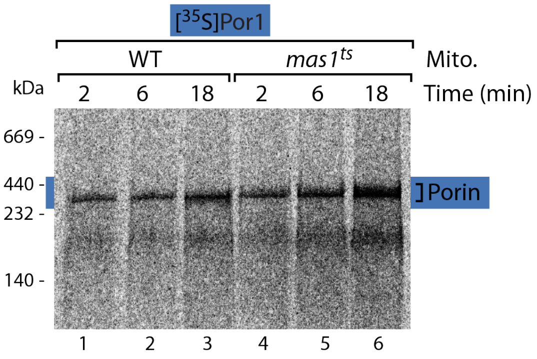

Rebuttal Figure R13. Import of radiolabeled Por1 precursors synthesized in yeast cytosolic extract. Import into *mas1*^{ts} mitochondria is enhanced upon mtUPR.

Reviewer #3 (Significance (Required)):

This contribution suggest an alternative model for the sequence of events in progress of mtUPR. Such a new model might be of general interest to many readers. However, the current data does not support this claim.

> We are happy that the reviewer is of the opinion that our new model on the progression of mtUPR is of general interest to many readers. We are convinced that our novel experiments together with the characterization of our model in our previous publication fully support our claims.

References

Gebert N, Joshi AS, Kutik S, et al. Mitochondrial cardiolipin involved in outer-membrane protein biogenesis: implications for Barth syndrome. *Curr Biol.* 2009;19(24):2133-2139.

Harbauer AB, Opalińska M, Gerbeth C, et al. Mitochondria. Cell cycle-dependent regulation of mitochondrial preprotein translocase. *Science*. 2014;346(6213):1109-1113.

Li G, Chen S, Thompson MN, Greenberg ML. New insights into the regulation of cardiolipin biosynthesis in yeast: implications for Barth syndrome. *Biochim Biophys Acta*. 2007;1771(3):432-441.

Osman C, Voelker DR, Langer T. Making heads or tails of phospholipids in mitochondria. *J Cell Biol*. 2011;192(1):7-16.

Poveda-Huertes D, Matic S, Marada A, Habernig L, Licheva M, Myketin L, Gilsbach R, Tosal-Castano S, Papinski D, Mulica P, Kretz O, Kücükköse C, Taskin AA, Hein L, Kraft C, Büttner S, Meisinger C, Vögtle FN. An Early mtUPR: Redistribution of the Nuclear Transcription Factor Rox1 to Mitochondria Protects against Intramitochondrial Proteotoxic Aggregates. Mol Cell. 2020 Jan 2;77(1):180-188.e9. doi: 10.1016/j.molcel.2019.09.026.

Schlame M. Cardiolipin remodeling and the function of tafazzin. *Biochim Biophys Acta*. 2013;1831(3):582-588.

Suhm T, Kaimal JM, Dawitz H, et al. Mitochondrial Translation Efficiency Controls Cytoplasmic Protein Homeostasis. *Cell Metab*. 2018;27(6):1309-1322.e6.

Vögtle FN, Brändl B, Larson A, et al. Mutations in PMPCB Encoding the Catalytic Subunit of the Mitochondrial Presequence Protease Cause Neurodegeneration in Early Childhood. *Am J Hum Genet*. 2018;102(4):557-573.

Vögtle FN. Open questions on the mitochondrial unfolded protein response [published online ahead of print, 2020 Sep 22]. *FEBS J*. 2020;10.1111/febs.15569.

Vögtle FN, Keller M, Taskin AA, et al. The fusogenic lipid phosphatidic acid promotes the biogenesis of mitochondrial outer membrane protein Ugo1. *J Cell Biol*. 2015;210(6):951-960.

Xu Y, Schlame M. The turnover of glycerol and acyl moieties of cardiolipin. *Chem Phys Lipids.* 2014;179:17-24.
