# Supplementary material for: Increased mitochondrial protein import and cardiolipin remodelling upon early mtUPR
Source: PLoS Genet. 2021 Jul 2;17(7):e1009664. doi: 10.1371/journal.pgen.1009664 (PMC8282050; doi:10.1371/journal.pgen.1009664)
Supplement: Attachment — Submitted filename: Response to reviewers_Poveda-Huertes and Taskin et al.pdf [file pgen.1009664.s013.pdf]

Reviewer #1 (Evidence, reproducibility and clarity (Required)):

Cellular response to defective mitochondrial functions like mtUPR attracts much attention and becomes a hot topic in the cell biology field. In this work, Poveda-Huertes et al. reported interesting results that defective presequence processing due to temperature-sensitive mutation of MPP (the mas1-ts mutation) is associated with enhanced in vitro import of both presequence-containing and presequence-lacking precursor proteins into isolated mitochondria, and with a marginal increase in the cardiolipin (CL) level and decrease in the level of its precursor lipid CDP-DAG in mitochondria.

> We fully agree with the reviewer that mtUPR is a hot topic in the cell biology field and are happy that the reviewer finds our results interesting.

A major problem of this work is, in contrast to the defective import machineries or decreased membrane potential of mitochondria, that the effects of defective presequence cleavage are not clear. In wild-type mitochondria, cleaved presequence peptides are depleted by peptide degradation in the matrix. However, in mitochondria with defective presequence cleavage, presequence-containing precursor proteins will be accumulated in the matrix, which may cause toxicity to the cell, but its mechanism is not clear; perhaps, accumulation of presequence-containing precursor proteins in the matrix may perturb the membrane structures of the inner membrane, trap matrix chaperones like mtHsp70 and mtHsp60, and so on. Thus, it is not appropriate to call such an unclear outcome of the defective presequence cleavage "mtUPR".

> We have analyzed all of these points raised by the reviewer in our previous publication, in which we in detail describe the toxic effect of defective presequence processing on the cell and characterize the mtUPR triggered by this defect (Poveda-Huertes et al., Mol. Cell 2020). Thus, the effects of the *mas1^{ts}* mutation on the cell are well-established and clearly characterized as mtUPR.
We fully understand that without knowing the *mas1^{ts}* mutant model to study mtUPR, the present study indeed may appear 'preliminary' to the reviewer. We are now introducing our mtUPR model in more detail in the introduction of the manuscript.
To shortly explain this model, the *mas1^{ts}* mutation used in the present manuscript is based on a patient mutation we identified and characterized in our publication Vögtle et al., American Journal of Human Genetics (2018). We further show in Poveda-Huertes et al., Mol. Cell 2020 that defects in MPP processing result in accumulation of non-processed precursor proteins in the mitochondrial matrix. These precursors rapidly form aggregates that cannot be degraded by the mitochondrial proteolytic system. Furthermore, we showed that this precursor aggregation in the matrix results in a fast transcriptional response (already after 2 hours) that triggers an increase in mitochondrial proteases and chaperones, which is a classic characteristic of mitochondrial unfolded protein responses (mtUPR) across species (see also comment to reviewer 3 below). We have added two figures of the data from this previous

publication below and hope that this clarifies the points of the reviewer (see also comment of reviewer 2 on our previously published results, which provided the basis of this manuscript). Taken together, the outcome of the mutation is well characterized and an excellent model to investigate the different steps and characteristics of mtUPR.

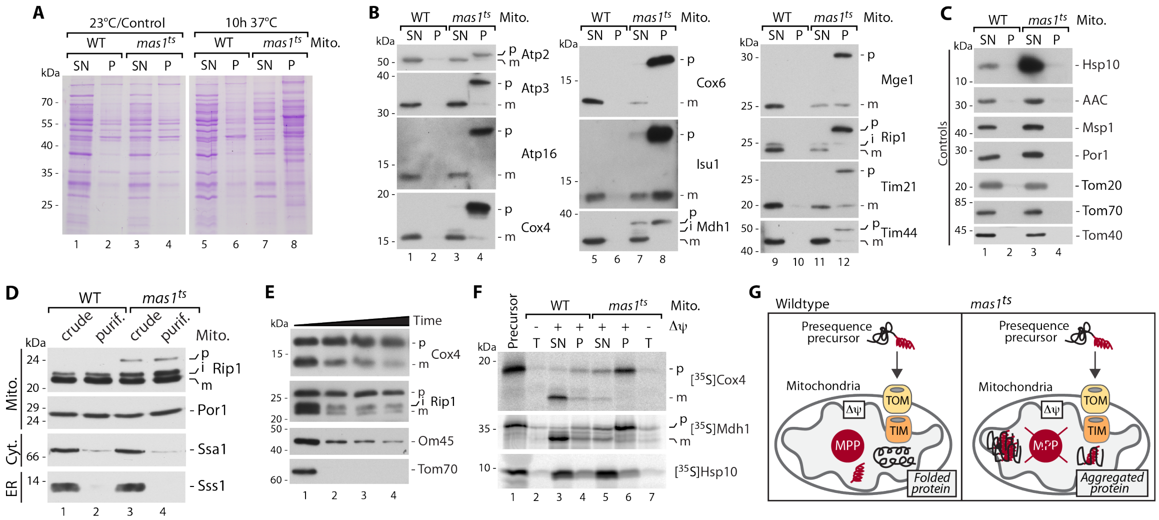

Compendium of Figure 1 and S1 of Poveda-Huertes et al., Mol. Cell 2020, which establishes the *mas1^{ts}* mutant as a mild and reversible model to study mtUPR induced by aggregating non-processed precursor proteins in the mitochondrial matrix.

(A) Coomassie-stained gels from SDS-PAGE analyses of wild-type (WT) and *mas1^{ts}* mitochondria isolated from cells grown under respiratory conditions and separated into soluble (SN, supernatant) and aggregated (P, pellet) protein fractions.

(B) Mitochondria isolated from WT and *mas1^{ts}* cells grown for 10 hours at 37°C were separated into soluble (SN, supernatant) and aggregated (P, pellet) protein fractions and analyzed by SDS-PAGE and immunodecoration. All non-processed precursor proteins were detected in the aggregated fraction, while processed, mature proteins were recovered as soluble proteins.

(C) Analysis as in (B) of non-processed proteins. The strong increase in the chaperone Hsp10 in the *mas1^{ts}* samples is a first indication of a mitochondrial unfolded protein response.

(D) Comparison of non-processed precursor proteins accumulating in crude and highly purified mitochondria reveal that aggregates are within the organelle. Ssa1, cytosolic marker; Sss1, ER marker.

(E) In organello degradation assay using *mas1^{ts}* mitochondria (isolated after growth for 10 hours at 37°C) reveals that precursors cannot be degraded as efficiently as mature proteins.

(F) Newly imported precursor proteins that cannot undergo processing rapidly aggregate directly upon import.

(G) Cartoon of import into WT and *mas1^{ts}* mitochondria with subsequent aggregation of non-processed precursor proteins.

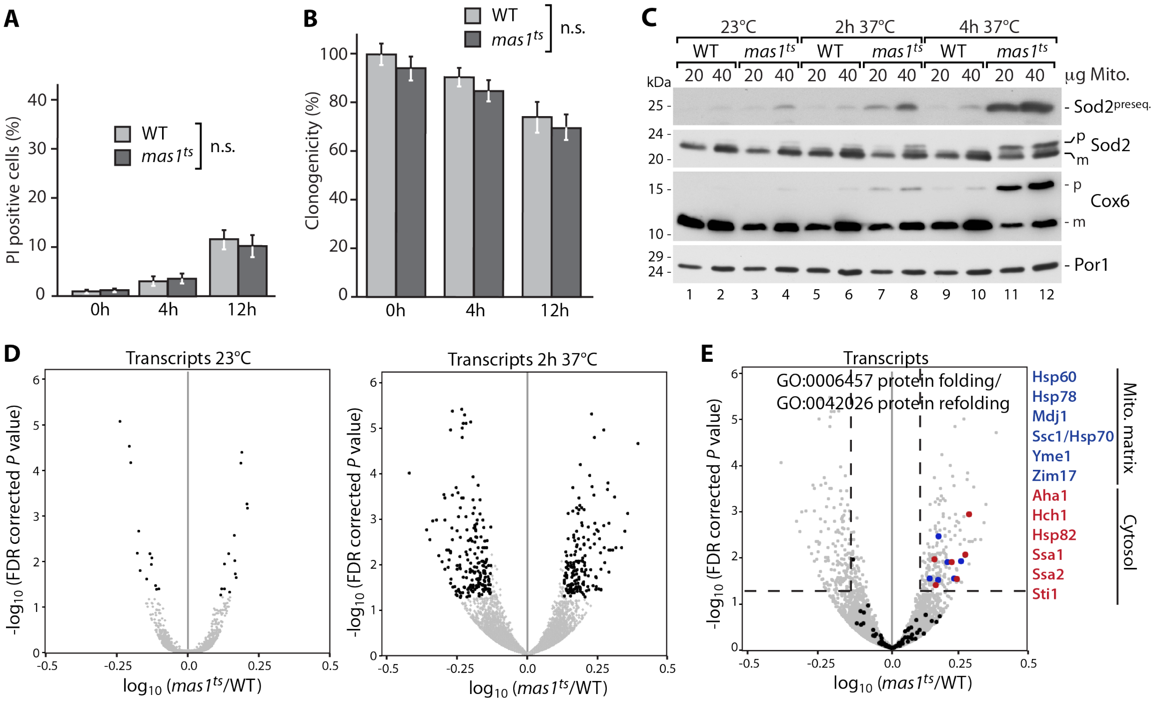

Figure 2 of Poveda-Huertes et al., Mol. Cell 2020

(A) Cell death determined by flow cytometric quantification of propidium iodide (PI) staining indicative of loss of plasma membrane integrity of wild-type (WT) and *mas1^{ts}* cells after shift to 37°C for indicated time. n = 12, data represent means +/- SEM; n.s., not significant. *mas1^{ts}* cells do not show increased cell death upon MPP inactivation.

(B) Determination of clonogenicity via survival plating of WT and *mas1^{ts}* cells after shift to 37°C for the indicated time. n = 6, data represent means +/- SEM.

(C) Immunoblot analysis of WT and *mas1^{ts}* mitochondria isolated from strains shifted for indicated times to non-permissive temperature. Sod2$^{preseq.}$, antibody generated against presequence peptide of Sod2. Accumulation of precursor proteins can be detected already after two hours of MPP inactivation.

(D and E) Distribution of transcripts quantified by RNA-seq in WT and *mas1^{ts}* cells. Displayed are Benjamini-Hochberg adjusted P-values. GO terms provided by Saccharomyces genome database. FDR, false discovery rate. *mas1^{ts}* cells trigger a rapid transcriptional response after MPP inactivation and up-regulate mitochondrial chaperones.

Besides, at this moment, there is no clear causative relationship between the enhanced in vitro import of several precursor proteins and changes in some lipid levels including the CL level. Therefore, this work is too premature to draw any conclusion or model to speculate the role of CL in overcoming the toxicity of the mas1-ts mutation or defective presequence processing.

> There seems to be a misunderstanding: We do not claim or propose a model that changes in cardiolipin are overcoming the toxicity of the *mas1^{ts}* mutation. We propose a model, in which as part of the mtUPR the cells adapt the mitochondrial lipid

composition by increasing and remodelling their signature lipid cardiolipin to stabilize the mitochondrial import translocases to enhance protein biogenesis. This model is fully supported by our data showing that import into all four mitochondrial subcompartments increases upon mtUPR (Fig 1) and that CL levels increase and are remodelled under these conditions (Fig 2). Only upon deletion of the gene coding for the cardiolipin synthase Crd1, this increased import rates are abrogated (Fig 4), thereby connecting changes in CL with the import effect upon mtUPR.

We provide now further experimental support for our model showing genetic interaction between the *mas1*^{ts}-mediated mtUPR and cardiolipin remodelling: We find that deletion of *TAZ1* (Taz1 is required for CL remodelling; Schlame, BBA 2013) also impacts on growth in *mas1*^{ts} like the deletion of *CRD1* demonstrating that cardiolipin remodelling is required upon mtUPR (see **novel Figures 3B and 3D**, reviewer 3). Furthermore, we tested several other phospholipid biosynthesis enzymes and deletions in no other lipid pathways had an effect on the *mas1*^{ts} cells (**Supporting information Fig 8A**, reviewer 3), clearly showing a specific role for cardiolipin synthesis (Crd1) and remodelling (Taz1) upon mtUPR.

We present now additional experimental data supporting the link of lipid changes and mitochondrial protein import upon mtUPR: We assessed the extraction of the inner membrane translocases from the lipid bilayer using different concentrations of the mild detergent digitonin. Both inner membrane protein translocases TIM22 and TIM23 show a different profile in WT and *mas1*^{ts} mitochondria isolated after 10 hours of mtUPR induction, as for the *mas1*^{ts} mitochondria higher amounts of digitonin are required for extraction of the translocases from their lipid environment (**Supporting information Fig S8B**, below).

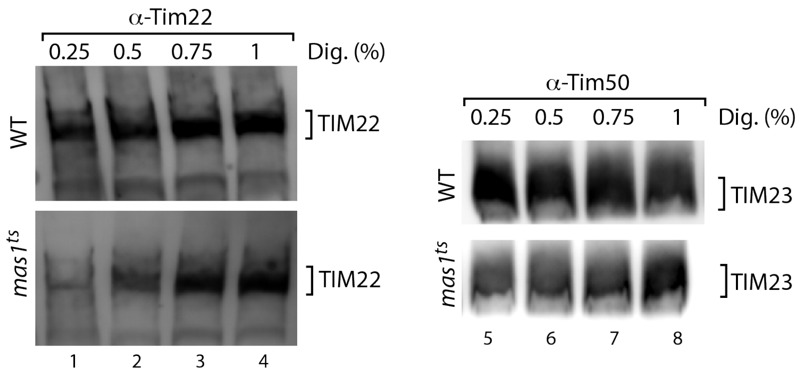

Novel Supporting Figure S8B. Assessment of translocase extraction from the mitochondrial lipid bilayer. Isolated mitochondria from wild-type (WT) and *mas1*^{ts} cells grown for 10 hours at 37°C were solubilized with different digitonin concentrations. While the inner membrane translocases were already efficiently extracted with low digitonin concentrations in wild-type mitochondria, in the *mas1*^{ts} mutant higher amounts of detergent concentrations were necessary. This indicates that the lipid environment embedding the translocases in the IM is modulated upon mtUPR.

We also performed this experiment with mitochondria isolated after 4 hours of mtUPR induction and could detect different extraction profiles for TIM22 already at this early time point (see novel **Figure 3E**, reviewer 3). Furthermore, upon deletion of *CRD1* these differences in translocase extraction were abolished indicating that differences in cardiolipin are responsible for the different extraction profiles upon mtUPR (novel **Supporting Figure S8C**, below). This indicates that the lipid composition surrounding the translocases changes early upon mtUPR, fully supporting our model that changes in CL stabilize the import machineries to support import upon mtUPR.

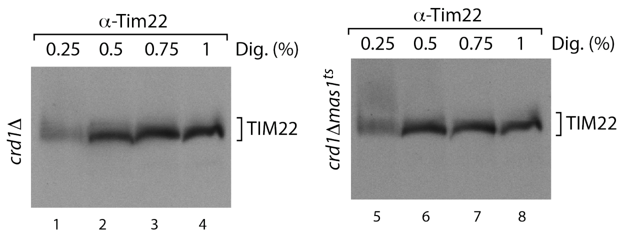

Novel Supporting Figure S8C. Extraction of TIM22 translocase from the lipid bilayer with increasing amounts of digitonin. Mitochondria were isolated from *crd1Δ* and *crd1Δmas1*^{ts} cells grown for 10 hours at 37°C.

The followings are additional concerns for this manuscript. 1)In vitro protein import into isolated mitochondria is a powerful tool to analyze the mitochondrial functions. However, the properties of mitochondrial protein import are not always the same between in vitro and in vivo. Therefore the authors should test if similar enhanced import is observed for mitochondrial proteins in vivo by assessing the levels of transiently expressed mitochondrial proteins etc.

> We have assessed protein import upon mtUPR in vivo as suggested by the reviewer. Induction of mtUPR results in transient increased gene expression of mitochondrial chaperones. We assessed protein steady state levels of the heat shock protein Hsp10 and indeed detected a strong increase upon mtUPR (see **Supporting Figure S6B**, below). This observation that protein import is increased upon mtUPR in vivo is actually the starting point of our manuscript that addresses the conundrum of increased protein import upon mtUPR when the current model suggests that protein import should be blocked.

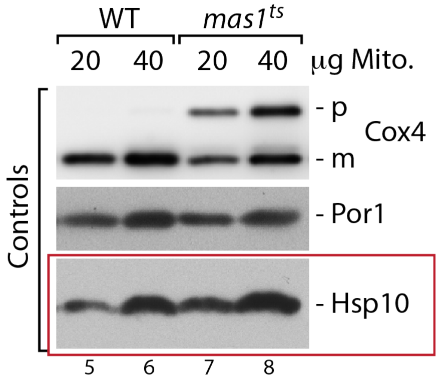

Supporting Figure S6B. The mitochondrial chaperone Hsp10 is strongly increasing upon mtUPR. Newly synthesized Hsp10 is efficiently imported from the cytosol into the mitochondrial matrix under stress conditions demonstrating that protein import is increased upon mtUPR in vivo.

If protein import would be impaired in vivo this would result in decreased protein steady state levels. We provide an example for such a scenario now in **Rebuttal Figure R1** (below), in which we analyze the protein levels of Hsp10 in *pam16-3* mutant mitochondria, that are characterized by a protein import defect due to mutations in the import motor PAM. Upon defects in protein import Hsp10 levels decrease. In contrast, protein import upon mtUPR in our model is clearly enhanced in vivo.

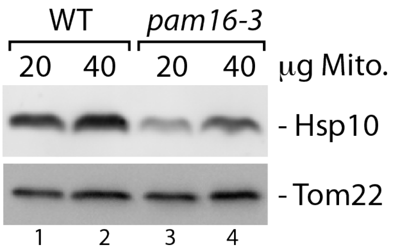

Rebuttal Figure R1. Example for decrease in steady state protein levels of Hsp10 upon defects in mitochondrial protein import (*pam16-3*, mitochondria with mutations in the protein import motor PAM). Tom22 serves as outer membrane loading control that is not affected by dysfunction of the matrix import motor PAM.

We then use in organello import assays to mechanistically dissect the four different import pathways into mitochondria upon mtUPR. Intact isolated mitochondria in combination with radiolabeled precursor proteins are used as the standard in the research field of mitochondrial protein biogenesis since several decades. All of our current knowledge on the mechanistic functioning of the import machineries is based on these in organello assays (pioneering work from the groups of Schatz, Neupert, Pfanner, Endo and others). The advantage of the in organello system is that the import competence of the translocation machineries can be directly assessed. These assays resulted in the identification of the role of CL in protein import upon mtUPR, which we further confirmed in vivo by the genetic interaction of *mas1*^{ts} with genes encoding for CL biogenesis enzymes (see **Figures 3A and 3C** and novel **Figures 3B and 3D**).

2)It is known that a decrease in the CL level impairs mitochondrial protein import, but this does not mean that increase in the CL level increases the efficiency of mitochondria protein import. If this is the case, the CL level in wild-type mitochondria is not optimized for the most efficient import of mitochondrial proteins, which is a very provoking hypothesis that needs to be experimentally tested.

> Dynamic changes in the lipid composition are a mean for the cell to adapt to changing demands, e.g. upon heat shock or metabolic changes. Also, under stress conditions it is conceivable that changes in lipids are a mean to protect the cell from deleterious effects. However, the reverse conclusion that import is not optimized under standard conditions is not accurate. The cell adjusts its needs under each condition and this is always a trade-off between different and sometimes opposing factors. Here, we show that upon mtUPR cells re-adjust their CL levels, which is crucial for the cell to support import of newly translated mitochondrial chaperones and proteases. This increased import is made possible by the stabilization of the import translocases, which we now show by titration of digitonin (see **novel Figures 3E, S8B and S8C**).

The cell is probably maintaining crucial functions at an optimum for each particular scenario in the complicated interplay of all global cellular processes. As a consequence, these different functions cannot be kept at their maximum capacity at all times. If all biological processes would always be kept at their maximum capacity, the cell would not have the means and possibilities to regulate and adapt upon changing cellular demands. One example is the mitochondrial ribosome: Mutations in the accuracy center by insertion of a point mutation in the Mrps12 subunit results in hyperaccurate translation. However, this is not beneficial, but on the contrary results in severe respiratory growth defects due to a decreased translation rate. Similarly, introduction of hypoaccurate mutations also showed a growth defect, this time caused by the resulting mistranslation of proteins (Suhm et al., Cell Metabolism 2018). Also, the main import gate into mitochondria TOM was shown to be regulated by phosphorylation in specific phases of the cell cycle adapting import of specific precursor proteins to cellular demands (Harbauer et al., Science 2014). These examples show that the activity of biological processes is a trade-off between a plethora of factors and functions. That the mitochondrial import machinery has the possibility to adapt upon changes in cellular states, e.g. to stabilize the import machineries upon mitochondrial stress upon which import is crucial to rescue mitochondrial defects, seems therefore very reasonable.

3)It is important to reveal the mechanism of the increased CL level. For this purpose, the authors should test the levels of Crd1, Gep4, Pgs1, and Tam41. Indeed, the reason for the CL level change may be complicated since the level of PE decreased and the level of PS increased with time in mas1-ts mutant mitochondria (Fig. 2A).

> The changes in PE and PS indicated by the reviewer are occurring with the same dynamic also in the wild-type control and are therefore not specific to mtUPR induction (see **Figure 2A**). We have also analyzed several additional deletions of proteins involved in lipid metabolism and only detected growth defects upon deletion of *CRD1* and *TAZ1* in *mas1*^{ts}, which are both key enzymes in cardiolipin biosynthesis (see **novel Figures 3B and 3D and S8A**, reviewer 3). Furthermore, only the changes in CL levels show a clearly different dynamic when comparing the *mas1*^{ts} with the wild-type control.
To investigate the mechanism behind the changes in CL levels, we have analyzed the steady state levels of proteins involved in cardiolipin biosynthesis for which specific antisera were available as suggested by the reviewer. We have identified an increase in Ups1 that plays a crucial role in CL metabolism by transporting the precursor phosphatidic acid from the outer to the inner mitochondrial membrane, where CL lipid synthesis takes place (Li et al., BBA 2007). We also detected an increase in the protein Pgs1 (phosphatidylglycerolphosphate synthase) that catalyzes the synthesis of phosphatidylglycerolphosphate from CDP-diacylglycerol (CDP-DAG), which is actually the first and rate-limiting step of CL biosynthesis (**novel Figures 2F and 2G**). The increase in Ups1 and Pgs1 are likely the mechanistic foundation for the changes in CL upon mtUPR, fully supporting our model.

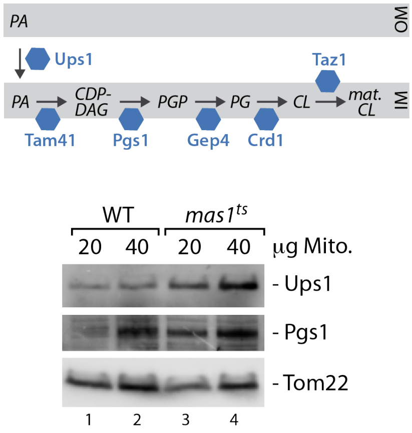

Novel Figures 2F and 2G. (Top panel) schematic cartoon of cardiolipin synthesis pathway. (Bottom panel) Steady state protein levels of isolated mitochondria from wild-type (WT) and *mas1*^{ts} cells grown for 10h at 37°C. Protein levels of Ups1 and Pgs1 increase upon mtUPR, while the control Tom22 does not.

Reviewer #1 (Significance (Required)):

This work is potentially interesting in terms of the cellular response to the defective mitochondiral proteostasis or mtUPR. However, the work is too preliminary stage and requires more experiments to draw an entire picture of the observed results.

> We have addressed all points of the reviewer with novel experiments and hopefully also explained the suitability of the underlying mtUPR model (Poveda-Huertes et al., Mol. Cell 2020) for the present study. With this model we could now for the first time systematically dissect the early and late mtUPR stages and clarify the long-standing confusion in the field about the role of protein import upon mtUPR. Our study clearly shows that protein import is (unexpectedly) increased upon early mtUPR stages and only compromised upon late mtUPR stages. **Thus, against the present dogma in the field, we conclude that impaired protein import is not cause but a consequence of mtUPR induction.**

Reviewer #2 (Evidence, reproducibility and clarity (Required)):

The authors in this study focussed on the analysis of earlry stages of mtUPR, using a genetic mutation of the Mas1 gene that creates a temperature-sensitive phenotype. They argue that early stages of mtUPR do not involve any substantial changes in inner membrane potential and characterized by an increase in all major protein import pathways. Decreased protein import is observed on the other hand only for later stages of mtUPR indicative of sustained stress. The concept they propose is very intriguing and the potential interest of this work is significant. I agree that, so far, essentially all studies of mtUPR utilized very severe stressors (CCCP treatment of mitochondria for example), which are very harsh and probably do not allow to study what happens in the early stages of mtUPR where the stress imposed is milder. Their previous work on this aspect of mtUPR clarified this idea and in fact supported very well this distinction of early vs

late stages of mtUPR. The main point of the work here is that the early stages of mtUPR are linked to a remodeling of cardiolipin (which is enriched in the mitochondrial inner membrane), and that this remodeling boosts the protein import processes.

> We are very happy and thank the reviewer for this positive comment on our manuscript and the appreciation of our previous work (Poveda-Huertes et al., 2020), in which we identified the novel mtUPR pathway using the *mas1*^{ts} mutant and which is the ideal model for the analysis presented in the current manuscript.

Main points:

1.The effect of cardiolipin remodeling should be primarily affecting import pathways for which the inner membrane is involved. How can they explain the effects on the Tim9 pathway which does not require an electrochemical potential and no association with the inner membrane?

> The reviewer is right, CL is most abundant in the inner membrane, where it is present with approximately 16%, so that protein import into the inner membrane should be primarily affected. In addition, mass spectrometry-based analyses of highly purified outer mitochondrial membranes revealed that CL is also found with 1.6% in the outer membrane (Vögtle et al., Journal of Cell Biology 2015; Gebert et al., Current Biology 2009). Even very low abundant lipids can have a strong effect on protein biogenesis: We have previously demonstrated that phosphatidic acid, which is present in the outer membrane with only 0.4%, is crucial for import and assembly of the fusion protein Ugo1. Decreasing the low PA amount further directly correlates with a decrease in Ugo1 assembly (Vögtle et al., JCB 2015). Furthermore, it was reported that the translocases of the outer membrane, among them the TOM complex, critically depend on CL for their functionality (Gebert et al., Current Biology 2009). Therefore, the import of all mitochondrial precursor proteins that use the TOM complex, which encompasses virtually all precursors, can be affected by changes in CL levels, among them Tim9 as shown in our study.

2.The authors talk about remodelling of CL upon mtUPR but they don't show the CL subspecies distribution in WT and Mas1-ts mutant cells before mtUPR in figure 2D. Since the total levels in uninduced Mas1-ts cells seem to be lower than in WT, and that increasing the temperature has an effect in the WT too, they should analyze the profile in uninduced cells in order to have a better idea of what it is going on. Also, Figure 2E shows a great variability in the levels of each CL subspecies in the three biological replicates, and how they have normalized this is not clear. In Figure 2G, they show that eliminating Crd1 in the WT also has a harsh effect on the growth of the WT cells at 35ºC (not as much as in the masts background, but still very noticeable).

> We have performed the analysis of CL subspecies distribution under permissive growth conditions as requested by the reviewer (**novel Supporting Figure S7A**, below).

Levels of certain CL subspecies are indeed a bit lower in the *mas1^{ts}* mutant under permissive conditions. The decrease of CL might be indicative of slight differences between WT and *mas1^{ts}* already under permissive growth conditions, which is also reflected by few transcriptional changes (see Figure 2 from Poveda-Huertes et al., 2020, reviewer 1). The comparison of the CL subspecies at permissive temperature and after 4 hours of induction now clearly demonstrates the dynamic change in CL upon mtUPR.

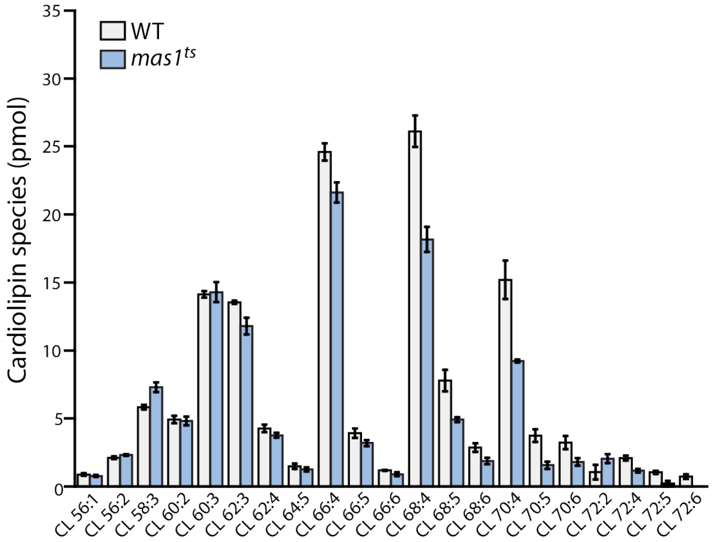

Novel Figure S7A. Quantification of indicated CL subspecies in wild-type (WT) and *mas1^{ts}* mitochondria isolated after growth at permissive temperature. n=3, data represent means ± SEM.

In Figures 2A-D and the novel Supporting Figure S7A, the abundance of phospholipids and cardiolipin species is shown in absolute values (pmol of the respective lipid detected in each sample). As shown in Fig. 2B, compromised Mas1 function results in an overall increase in cardiolipin content. To visualize the relative abundance of the different cardiolipin subspecies within these samples, a heat map was generated (Figure 2E and novel Supporting Figures S7B-D). Here, values have been standardized to each sample and are thus depicted as mol%. Thus, the total cardiolipin content in each sample is set to 100% and relative distribution of all cardiolipin subspecies is shown.

The reviewer is right that already deletion of *CRD1* has an effect on growth and it was published previously that its deletion results in a mild growth defect at elevated temperature (38°C) in wild-type cells (e.g. Gebert et al., Current Biology 2009). The synthetic growth defect upon deletion of *CRD1* in the *mas1^{ts}* background is more severe pointing to a genetic interaction of MPP function and cardiolipin biosynthesis. We have now in addition analyzed the effect of deletion of *TAZ1* as requested by reviewer 3, which shows the same growth defect as seen upon deletion of *CRD1* further underlining the specific effect and requirement for CL upon mtUPR (**novel Figures 3B and 3D**, reviewer 3).

3.In Fig 2C and D: It is not explained why at 2 and 4 hours there is such an increase in the CDP-DAG precursor also in the WT, that does not then translate in the increase in the levels of CL. Also, in the Mas1-ts mutant the 4-fold increase in the precursor at 2 hours that disappears at 4 hours translates in a modest increase in the total amounts of CL at 4 hours shown in Figure 2B. Also, are the units right? There are around 120 pmol total CL but only 0.5 pmol CDP-DAG so if this is right, it is questionable how much this can contribute to the formation of the total pool of CL. Can the authors please clarify this point?

> CDP-DAG is a precursor for several other phospholipids besides cardiolipin including phosphatidylcholine (PC) and phosphatidylinositol (PI). The increase in CDP-DAG in the wild-type might therefore be due to general lipid modulation upon the applied heat shock. Furthermore, CDP-DAG is a lipid precursor that is only transiently present and rapidly further modified to generate the indicated phospholipid species. It is short lived and of low abundance, and does not accumulate in mitochondria under normal conditions (Osman et al., JCB 2011), which is why it is only detected in low amounts in our lipidomics analyses.

4.In Fig3: the effect on AAC2 is not surprising as it is known that import and assembly of carriers proteins (and stability of them in membrane complexes) is affected by CL. It is not clear here whether the effect is truly on import or on assembly in the membrane. The authors can check whether proper insertion is affected if they analyze the imported AAC2 in mitoplasts with protease treatment which would normally give a smaller 2-3 kDa species if insertion is working well, but would result in further degradation if the AAC2 species is not properly inserted.

> The reviewer is absolutely right that AAC depends on the lipid composition during its biogenesis, but that also mature AAC requires several cardiolipin molecules for stable assembly. The assay suggested by the reviewer is an excellent idea and we have performed this experiment importing radiolabeled AAC2 into *crd1*Δ and *mas1*^{ts}*crd1*Δ mitochondria (**Rebuttal Figure R2**). Treatment of mitoplasts with Proteinase K results in the small size shift as described by the reviewer. The result shows that the kinetic is delayed in the *mas1*^{ts}*crd1*Δ mitochondria, demonstrating that import and insertion into the membrane is affected fully supporting our conclusion.

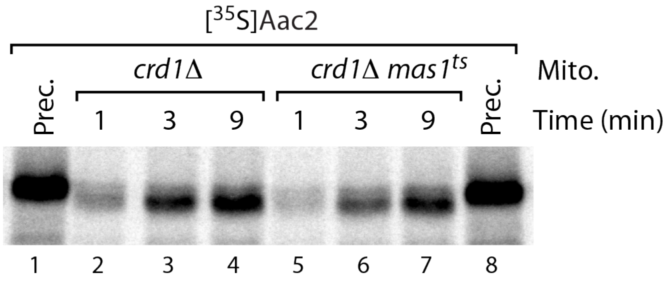

Rebuttal Figure R2. Import of radiolabeled AAC2 precursor into mitochondria. Samples were treated with Proteinase K after mitoplasting (rupture of outer membrane) resulting in the small size shift of Aac2.

Minor points:
1.The proposal that CL increase and subspecies remodeling likely stabilizes the mitochondrial import complexes thereby increasing protein import capacity into the organelle under stress conditions is an intriguing model. However, the authors show no changes in the steady-state levels of the import complexes upon stress in Figure supplement 3 or in the absence of cardiolipin remodelling in Figure supplement 5. In line with this, they say that there are no changes in the TOM complex upon stress because Tom20 import/assembly does not change (Figure supplement 1), although in this same figure the TOM complex increases if you look at the import of Tom40 and Tom22 at 60 minutes in Panels A, B and C. Can the authors explain this? There is increased import/assembly but no differences in steady-state levels of Tom40 and Tom22 (Figure supplement 3). Is it because there is also increased degradation? Have the authors looked into this?

> We have assessed the degradation of TOM subunits by in vivo degradation assays as suggested by the reviewer (**Rebuttal Figure R3**). We did not detect a difference in protein stability of TOM subunits in *mas1*^{ts} and WT cells upon mtUPR conditions.

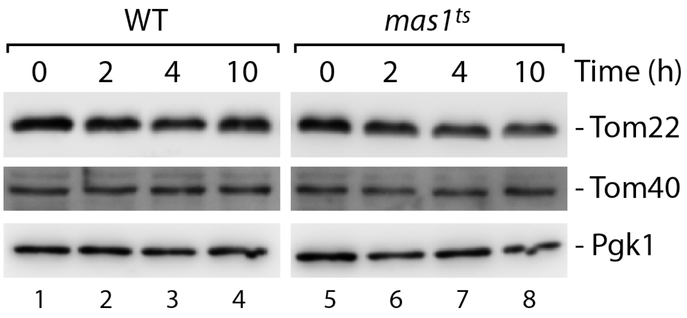

Rebuttal Figure R3. In vivo degradation monitored via SDS-PAGE and western blotting. Cells were treated with cycloheximide and incubated for the indicated time.

We also have novel data on the extraction of translocase complexes from the lipid bilayer that indicate that the lipid surrounding of the translocases is modulated upon mtUPR (see **novel Supporting Figures S8B and S8C**, reviewer 1, and **novel Figure 3E**, reviewer 3). As a further explanation for the observation that the TOM complex is not increasing we would like to point out that the radiolabeled precursors used in our analyses were chosen as model substrates for the different mitochondrial import routes. They were used as a mean to assess the activity of the import machinery in general. While from these results it is conceivable to deduce that protein import into mitochondria is generally enhanced upon mtUPR, this does not mean that more Tom subunits will be imported in vivo (only if their transcription/translation increases, more proteins could be imported. However, transcription of e.g. Tom subunits does not increase upon mtUPR (see Figure S6A)). We expect that rather those proteins that are increased on a transcriptional level, e.g. chaperones and proteases, will benefit from the increased import capacity and result in increased protein levels (as shown for Hsp10 in Figure S6B).

2.The times after induction of mtUPR in which they have done the import assays should be explained. In material and methods it is indicated that 10 hours after stress were used as indicative for 'early' effects, and 20 hours for the 'late' effects. However, the CL remodelling was assessed after 2 and 4 hours. There is a discrepancy here which I don't understand. In fact this does not allow an accurate interpretation of the data because things could change between 4 and 10 hours. In this respect, I think an interesting point of the paper is the difference in the import between the 'early mtUPR' and the 'late mtUPR' shown in Figure supplement 4. This is an important point of the paper and I recommend this should be included as a main figure. Also, there should be quantifications and statistical analysis like in Figures 1 and 3. They should also assess what happens to cardiolipin in this late mtUPR if they want to make the direct relation between 'cardiolipin remodelling' and import efficiency in response to the different phases of mtUPR. Is it possible that at late stages of mtUPR CL persists and some of it is rearranged to the outer membrane where it could in fact act as a cell death triggering signal? Is there a 'threshold' of CL remodeling above which mitochondria are committed to mitophagy and apoptosis?

> The reviewer is right that we measured lipid changes after 2 and 4 hours of mtUPR induction and performed import experiments after 10 hours. The lipidomics analyses were performed after 2 and 4 hours to be able to detect the initial start and the dynamics of the changes in CL. CL is turned over much slower than other phospholipids so that once CL is assembled it remains inert to degradation (Xu and Schlame, 2014). Due to its long half-life changes in CL should indeed persist over a longer period of time as proposed by the reviewer. We have now addressed this by detailed lipidomic analysis of CL species after prolonged heat-shock of 10 hours, e.g. the conditions upon which import is strongly increased. Cardiolipin species clearly show a different remodelling of their CL acyl chains including differences in length and unsaturation, compared to WT also after 10 hours of heat shock (**novel Supporting Figures S7B-D**). This demonstrates that changes in CL are present under the conditions in which increased protein import is observed.

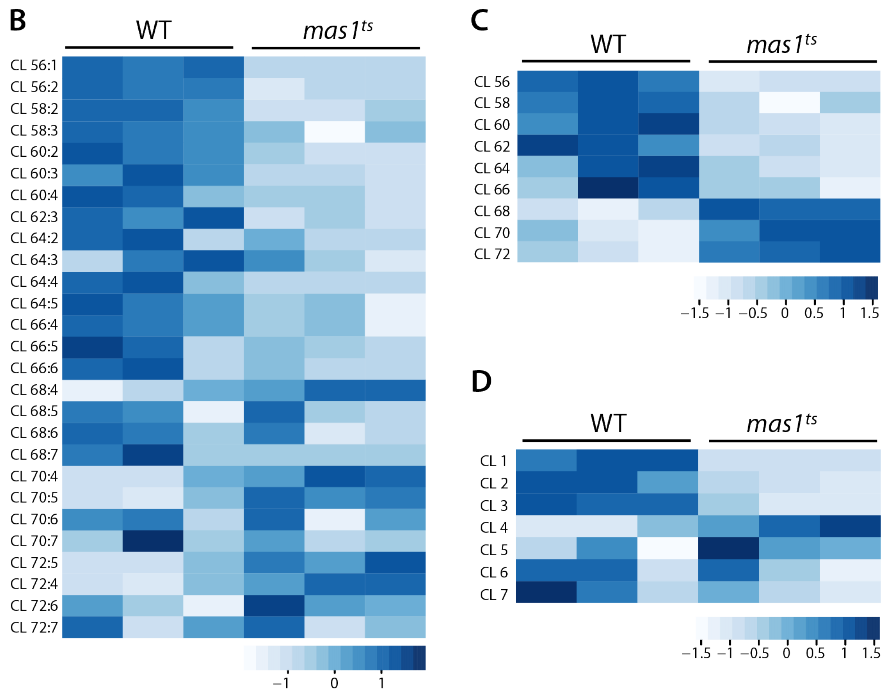

Novel Supporting Figures S7B-D. (B) Heatmap of distribution of different CL subspecies standardized to total CL content per sample in WT and *mas1^{ts}* mitochondria isolated after growth for 10 hours at 37°C. (C) Analysis of acyl chain length and (D) of number of double bonds. Shown are three biological replicates for each strain.

We have also obtained additional biochemical data that lipid changes impact on the import translocases after 10 hours of mtUPR: Digitonin titration to assess the extractability of the inner membrane translocases TIM22 and TIM23 revealed that while very low amounts of digitonin are sufficient to extract the complexes in controls, an increasing digitonin concentration is required upon mtUPR (see **novel Supporting Figure S8B** above, comment to reviewer 1). This indicates that the lipid environment around the translocases is indeed changing upon mtUPR and that these changes are maintained over a longer period of time.
Therefore, we have solved the discrepancy in the initial analysis by demonstrating changes in CL remodelling after 10 hours of MPP inactivation. Furthermore, we have also investigated protein import after 4 hours and already detected improved import upon this earlier time point (see **novel Supporting Figure S2**, reviewer 3). Also at this early time point the extraction of the inner membrane translocase TIM22 is changed upon mtUPR (see **novel Figure 3E**, reviewer 3).
Regarding the analysis of import competence upon late mtUPR we have performed quantifications and statistical analyses as requested by the reviewer and they are fully supporting our hypothesis that protein import into mitochondria only declines upon late stages in mtUPR (see **novel Supporting Figures S4A-D**, below).

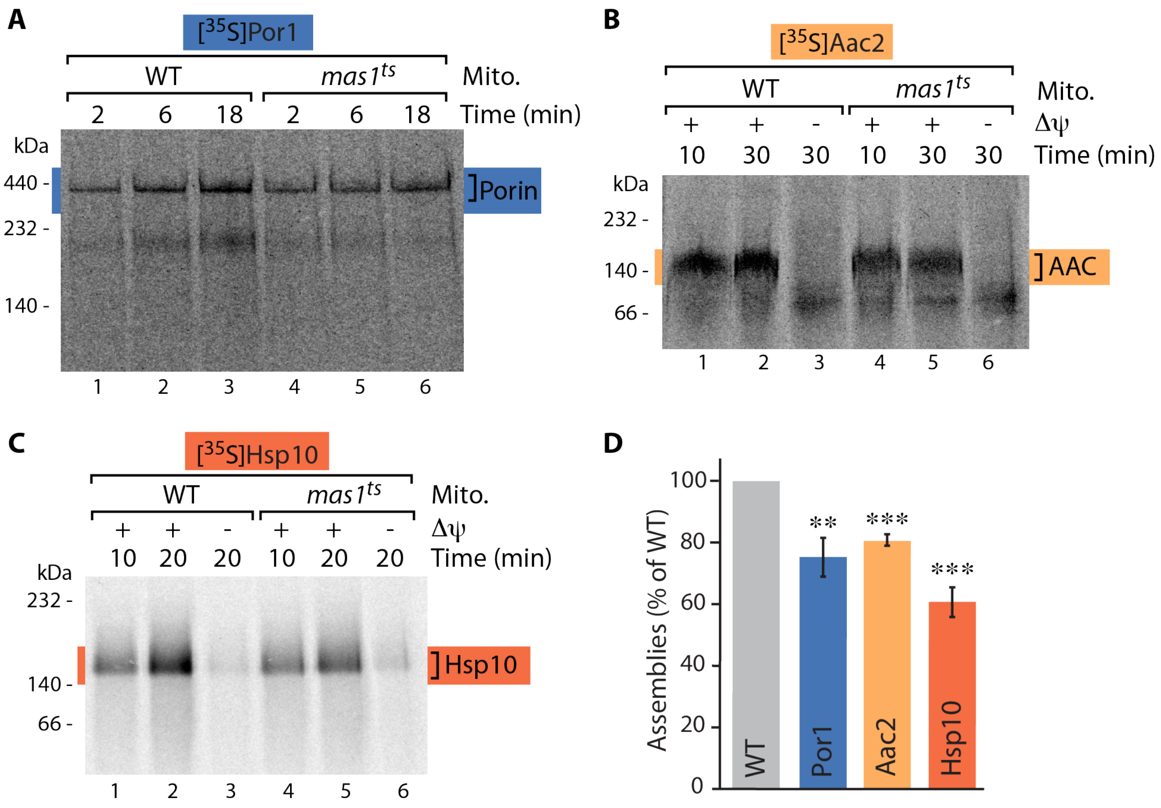

Novel Supporting Figures S4A-D. Import assays of indicated precursor proteins into mitochondria isolated from wild-type and *mas1^ts^* cells grown for 20 hours at 37°C. Upon this late mtUPR stage import into mitochondria is significantly reduced (quantification of last import time point in D). Quantification for n=3, data represent means +/- SEM.

It is an intriguing idea that the increase in CL upon prolonged mtUPR might serve as a signal for apoptosis, due to its exposure in the outer leaflet of the outer mitochondrial membrane. We have therefore assessed survival of *mas1^ts^* cells and could not detect an increase in cell death upon mtUPR induction (**Rebuttal Figure R5**, Reviewer 3).

3.The authors use the term mitochondrial dysfunction in several parts of the text (for example p. 5 and 7), but it is not clear they mean by that in the context of their study. Given that membrane potential, protein import and mtDNA expression are unaffected, what kind of dysfunction do they refer to? Please explain this point.

> We apologize for this inaccuracy. We will replace the term mitochondrial dysfunction in the text and will describe specifically the particular dysfunction, e.g. defects in MPP processing.

Reviewer #2 (Significance (Required)):

The potential advance of the paper is the definition of early stages of mtUPR and a potential mechanism (CL remodeling) that accompanies this. Also, the increase rather than the, commonly assumed, decrease is an unexpected new result that looks at the mtUPR pathways under a completely different angle.

The audience are researchers interested in mitochondria biogenesis, intracellular sorting and mechanisms of protein homeostasis in the cell.

My expertise is on mitochondria biogenesis, protein targeting and mitochondria biology in general.

> We thank the reviewer for this significance assessment of our study. We are especially happy about the comment that our work looks at mtUPR under a completely different angle.

Reviewer #3 (Evidence, reproducibility and clarity (Required)):

The mitochondrial unfolded protein response (mtUPR) is one of several mechanisms that have evolved to counteract mitochondrial dysfunction and maintain the health of the organism. This adaptive transcriptional response was studied mainly in C. elegans and based on findings in this system this process was suggested to be regulated by the mitochondrial import efficiency of the transcription factor ATFS-1. Upon mitochondrial dysfunction, import of ATFS-1 into mitochondria is reduced, allowing it to be trafficked to the nucleus where it promotes the expression of genes encoding mainly chaperones and proteases that promote survival and recovery of the organelle.
The impairment of genes involved in diverse aspects of mitochondrial function, addition of reagents like paraquat or doxycycline, and the accumulation of misfolded protein in the matrix of the organelle were reported to induce mtUPR. It was suggested that these impairments activate UPRmt because the import efficiency of the mitochondrial network is reduced.

In the present contribution the authors propose an alternative model where protein import is not inhibited, but rather actually enhanced upon mtUPR. This enhancement is linked to up-regulation in the synthesis and modelling of cardiolipin.

However, as I wrote in details below, I think that the experimental set-up does not allow the authors to obtain results that can support these claims.

> The experimental set-up is based on our previous publication on mtUPR (Poveda-Huertes et al., Mol. Cell 2020), in which we have analyzed all points raised by the reviewer in the first major issue below in great detail. We fully understand that without

knowledge of the *mas1*^{ts} model to study mtUPR, the present study indeed may appear 'preliminary' to the reviewer. Taking this validation of our model into account (which is also highlighted by reviewer 2), we are convinced, that we can use our experimental set-up to perform experiments and obtain results on mtUPR and that these results fully support our claims.

Major issues:

1.The authors claim that the transcriptional reprogramming, which is associated with the mtUPR starts already after 2 hrs at 37{degree sign}C. Nevertheless, they define as an "early stage" mitochondria that were isolated 10 hrs after the shift to the non-permissive temperature. Thus, using mitochondria that were isolated after 10 hrs cannot be considered as an "early" stage but in contrast might already harbor elevated levels of proteases and chaperones.
To obtain real information on the status of the isolated mitochondria, the authors should follow systematically (for example, after 1, 2, 5, 10, 15, and 20 hrs) the steady-levels of mitochondrial chaperones (like Hsp70, Hsp60/10) and proteases (Yme1, Yta10/12, Pim1, and others). A hint for elevated levels of chaperones are the high levels of the co-chaperone Mdj1 (Fig. Suppl. 3A), which the authors failed to comment.
The results of Fig. 2C demonstrate that already two hrs time difference (between 2 and 4 hrs) can cause major deviations in the outcome.

> All of these questions raised by the reviewer were addressed and answered in our previous publication (Poveda-Huertes et al., Mol Cell 2020).
We have analyzed the transcriptional changes upon mtUPR in the *mas1*^{ts} cells and have shown that the transcriptional response is rapid and that transcripts of mitochondrial chaperones and proteases increase. We also show that the transcriptional changes result in an increase in mitochondrial protein steady state levels of e.g. the heat shock protein Hsp10 (which is also used as a positive control in the present manuscript, see Figure S6B). Similarly, the increased transcripts of *MDJ1* served as a positive control in the qPCR analyses of Figure S6A (we apologize for not making this more clear in the current manuscript, we have added this in the figure legend of the revised version). Therefore, we are employing a well-characterized model for mtUPR.
The reversible and mild induction of mtUPR in the *mas1*^{ts} cells results in measurable kinetic changes that for the first time allow the dissection of early and lates stages.

In the present study we made use of the *mas1*^{ts} model to investigate the big conundrum in mtUPR research how increased transcription/translation of mitochondrial chaperones and proteases can result in increased steady state levels of these proteins within the organelle if mitochondrial protein import is blocked (which is the current mtUPR model). Our model allows dissection of the temporal stages in mtUPR and reveals that upon early time points in which transcription of mitochondrial chaperones and proteases is strongly increased, also the import capacity of the organelle is increased by changes in the signature lipid CL. This allows import of the newly synthesized chaperones and

proteases. We have added some of the key findings of our previous publication below.

We hope we could explain the reviewer the basis of our model system and its perfect suitability to systematically study the protein import pathways upon mild (*early* stage) and strong (*late* stage) induction of mtUPR.

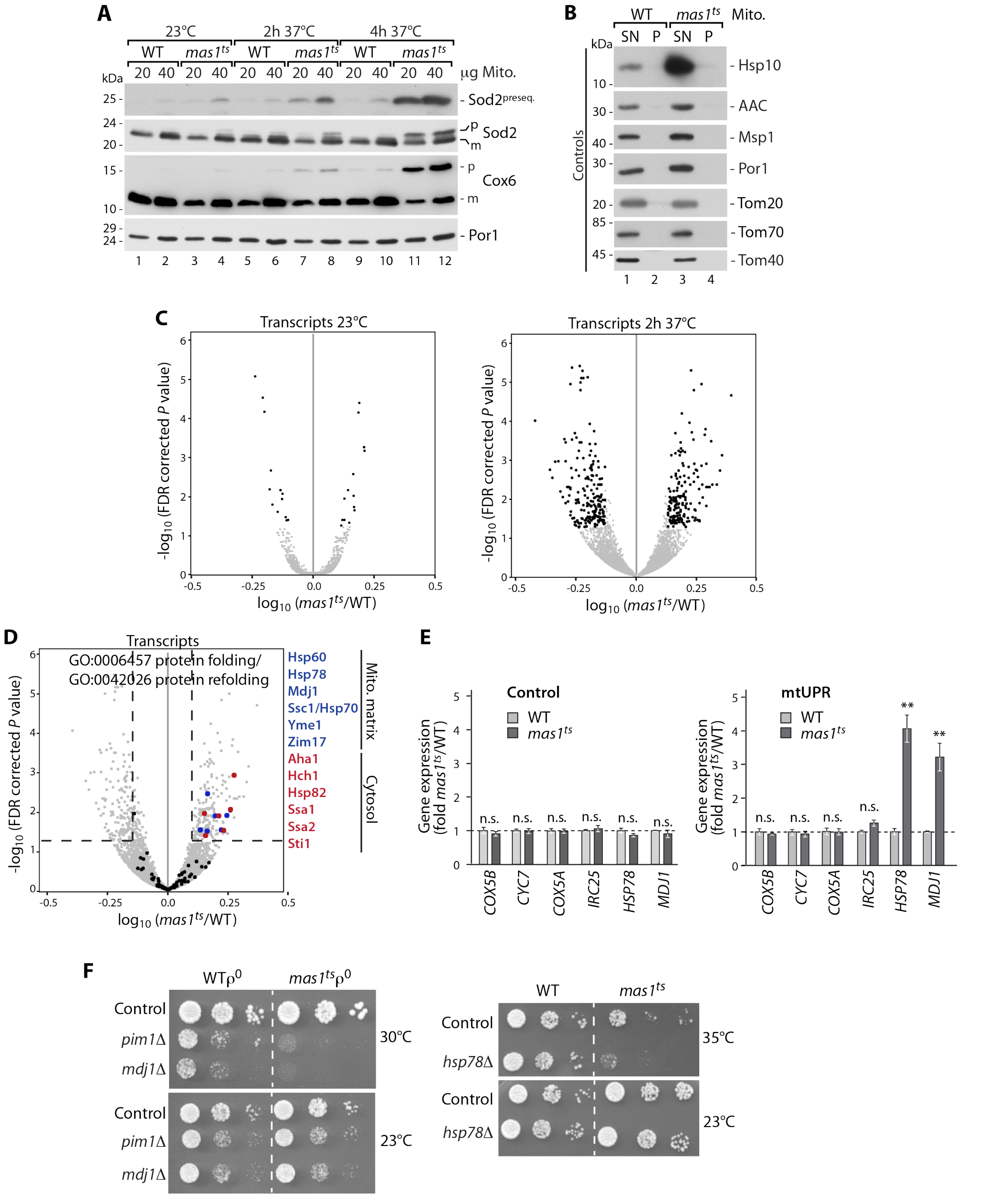

Compendium of Figure 2 and S2 from Poveda-Huertes et al., Mol. Cell 2020 (on next page).

(A) Immunoblot analysis of WT and *mas1*^{ts} mitochondria isolated from strains shifted for indicated times to non-permissive temperature. Sod2$^{preseq.}$, antibody generated against presequence peptide of Sod2. Accumulation of precursor proteins can be detected already after two hours of MPP inactivation.

(B) Mitochondria isolated from wild-type (WT) and *mas1*^{ts} cells grown for 10 hours at 37°C were separated into soluble (SN, supernatant) and aggregated (P, pellet) protein fractions and analyzed by SDS-PAGE and immunodecoration. The strong increase in the chaperone Hsp10 in the *mas1*^{ts} samples is an indication of a mitochondrial unfolded protein response.

(C and D) Distribution of transcripts quantified by RNA-seq in WT and *mas1*^{ts} cells. Displayed are Benjamini-Hochberg adjusted P-values. GO terms provided by Saccharomyces genome database. FDR, false discovery rate. *mas1*^{ts} cells trigger a rapid transcriptional response after MPP inactivation and up-regulate mitochondrial chaperones.

(E) Gene expression analysis of indicated genes by RTqPCR after growth at 23°C (control) or after 2 hours at 37°C (mtUPR). *HSP78* and *MDJ1* are encoding mitochondrial chaperones. Quantification for n=3, data represent means +/- SEM.

(F) Growth tests to assess synthetic effects of indicated mutant strains on *mas1*^{ts} and wild-type cells. In case of *pim1Δ* and *mdj1Δ* rho^{0} background was used due to the loss of mtDNA by the deletion.

2.The authors observed import reduction after shifting the cells for 20 hrs at the non-permissive temperature. What is the general status of cells after such long time at 37{degree sign}C? Is their mitochondrial morphology normal? How about the membrane potential of such isolated organelles? Do they duplicate in the time between 10 to 20 hrs? Without this information it is not clear if mas1ts cells after 20 hrs represent a mtUPR "late stage" or simply cells in bad shape.

> We have assessed key parameters of cellular and mitochondrial fitness after growth of *mas1*^{ts} cells at non-permissive temperature for 20 hours as requested by the reviewer. Growth curves of wild-type and *mas1*^{ts} cells at 37°C show that the *mas1*^{ts} mutant still divides under our stress conditions (**novel Supporting Figure S1B**, left panel below). We have compared our mtUPR model with some of the frequently used triggers of mtUPR (**Rebuttal Figure R4**, right panel below), in which induction of mtUPR immediately eliminates all cellular growth.

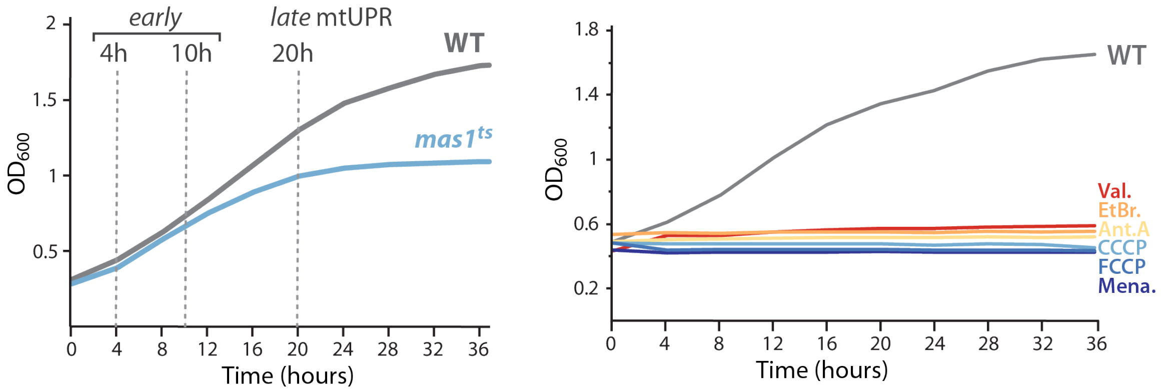

Novel Supporting Figure S1B and Rebuttal Figure R4. Growth curve of wild-type (WT) and *mas1*^{ts} cells on non-fermentable carbon source at non-permissive temperature (37°C) revealing that the *mas1*^{ts} mutant is dividing after 20 hours of induction (left panel). Growth curves in the right panel display previously used triggers for induction of mtUPR in the literature that all immediately inhibit cell division (Val., valinomycine 2 µM; EtBr., Ethidium bromide treatment 60 µg/mL; Ant. A, Antimycin A 1 µM; Mena, menadione 0.3 mM; CCCP, Carbonyl cyanide m-chlorophenyl hydrazone 10 µM; FCCP, Carbonyl Cyanide. P-(Trifluoromethoxy) Phenylhydrazone 10 µM).

We also assessed cell death in *mas1*^{ts} cells over 24 hours and could not detect increased cell death upon mtUPR compared to wildtype (**Rebuttal Figure R5**).

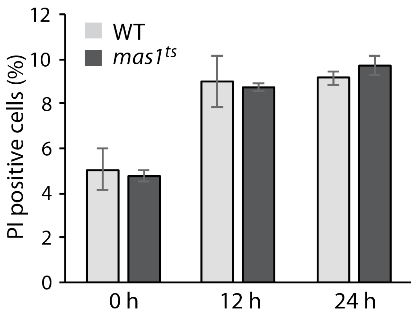

Rebuttal Figure R5. Analysis for PI positive cells (indicative of cell death) in wild-type and *mas1*^{ts} cells demonstrates that the *mas1*^{ts} cells do not show increased cell death after 24 hours of growth at non-permissive temperature.

Similarly, the membrane potential is largely unaffected after 20 hours, while addition of menadione to induce oxidative stress (a classic mtUPR trigger often used in other studies, reviewed in Vögtle, FEBS J 2020) completely depletes this key parameter of mitochondrial fitness (**novel Supporting Figure S4E** and **Rebuttal Figure R6**).

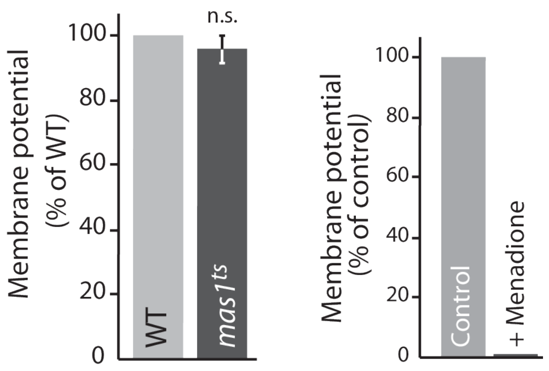

Novel Supporting Figure S4E and Rebuttal Figure R6. Membrane potential measurement using mitochondria isolated after growth at 37°C for 20 hours (left panel). The membrane potential in the *mas1*^{ts} mutant is comparable to wild-type. In contrast, mitochondria isolated after treatment with 0.3 mM menadione do not have a measurable membrane potential anymore.

Taken together, these data clearly show that the *mas1*^{ts} cells are not "in bad shape" and that the induction of mtUPR using our reversible *mas1*^{ts} model represents an excellent system to analyze different stages of mtUPR.

3.The changes in the phospholipids were determined after 2 or 4 hrs at 37{degree sign}C. Hence, it is hard to correlate these findings with the results regarding protein import which were determined after 10 or 20 hrs. Currently, a causative link between phospholipids change (measured after 4 hrs) and enhanced import (monitored at 10 hrs) is not possible. To establish a connection between altered lipid composition and changes in import efficiencies, the authors should analyze both aspects (lipidomics and protein import) after the same time periods at the non-permissive temperature.

> We show in Figure 4 that deletion of the cardiolipin synthase Crd1 impacts strongly on import competence in the *mas1*^{ts} cells thereby linking the enhanced protein import upon mtUPR to CL modulation. In addition, we show now that also deletion of *TAZ1* impairs growth of *mas1*^{ts} cells fully supporting our hypothesis that CL remodelling plays a role upon mtUPR (novel **Figures 3B and 3D**). CL is long-lived and we have now also additional experiments showing that CL changes persist after 10 hours (see novel **Supporting Figures S7B-D**, reviewer 2). Moreover, also the inner membrane translocases TIM22 and TIM23 are showing different lipid extraction profiles upon mtUPR compared to control at this time point (**novel Supporting Figure S8B**, reviewer 1). We have analyzed this difference in the lipid extraction profile also for mitochondria isolated after 4 hours of mtUPR induction and also here we find changes in the extractability of the inner membrane translocase TIM22 (**novel Figure 3E**). This indicates that the lipid environment around the inner membrane translocase is already remodelled after 4 hours.

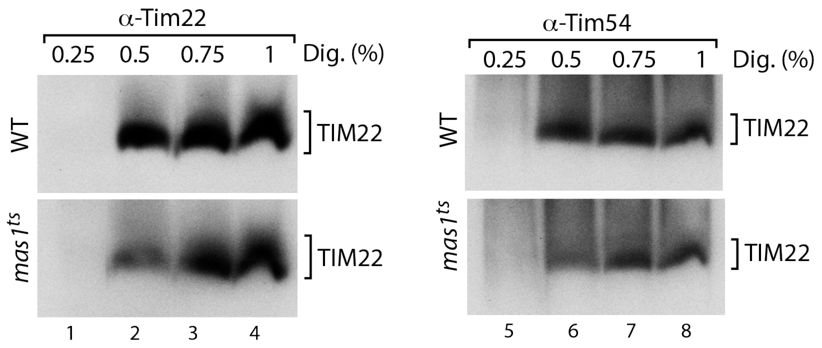

Novel Figure 3E. Assessment of translocase extraction from the mitochondrial lipid bilayer. Isolated mitochondria from wild-type (WT) and *mas1*^{ts} cells grown for 4 hours at 37°C were solubilized with indicated digitonin concentrations. The inner membrane translocase TIM22 requires higher amounts of detergent to be solubilized from the lipid bilayer upon mtUPR compared to control (WT).

Furthermore, we have analyzed the protein import pathways upon 4 hours of mtUPR induction as requested by the reviewer and detect an increase in import into the matrix and inner membrane already at this early time point (**novel Supporting Figure S2**). The import pathways into the matrix and inner membrane are most sensitive towards changes of cardiolipin and are therefore likely affected earlier than import into the IMS and OM, which are not changed after 4 hours (notably, they are not decreased), but strongly increase at later time points. Notably, the outer mitochondrial membrane contains much less CL than the inner membrane.

Therefore, we have demonstrated for both time points (4 and 10 hours of induction) that changes in CL species and changes in import are accompanied by each other.

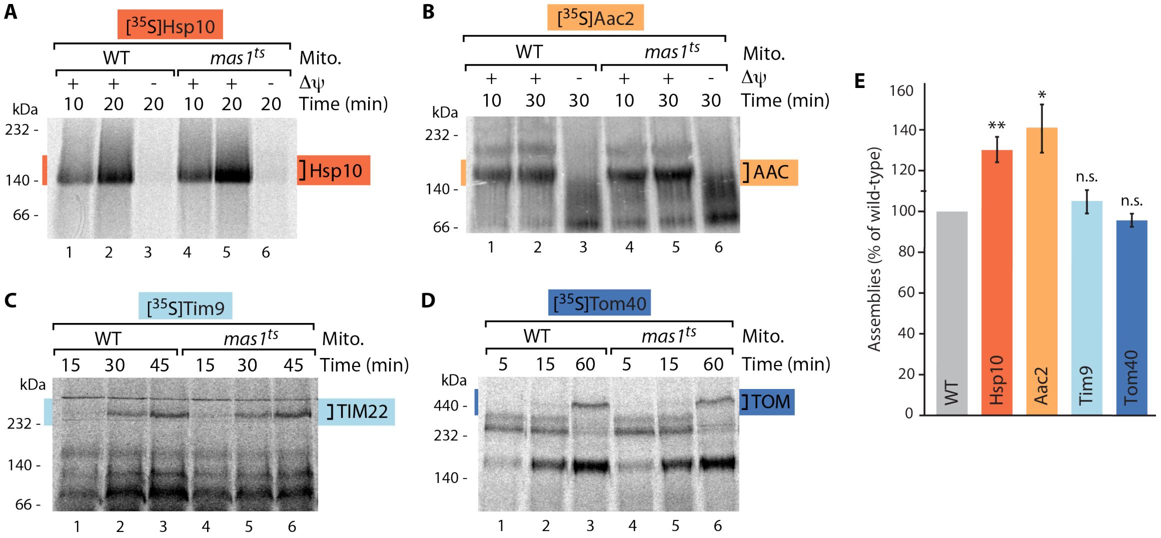

Novel Supporting Figure S2. (A-D) Import assays of indicated precursor proteins into mitochondria isolated from wild-type and *mas1*^{ts} cells grown for 4 hours at 37°C. Already upon this very short induction of mtUPR import into the matrix (Hsp10) and inner membrane (Aac2) is increased. Import into these two compartments is specifically sensitive to changes in cardiolipin. (E) Quantification of last import time point from (A-D). Quantification for n=3, data represent means +/- SEM.

4.Fig. 2F & G: The mas1ts cells are sick to start with. Thus, it is not surprising that the deletion of CRD1 on this background resulted in even further phenotypes. However, how specific are these observations? The authors should delete other genes that are relevant to lipid metabolism, for example PSD1 and UPS1/2, and test whether they observe similar or other results. Along the same line, Taz1 is important for the modelling of CL species. To study the relevance of CL modelling, the authors should create a mas1ts strain deleted for TAZ1.

> The *mas1*^{ts} model is a **conditional mutant**, which grows indistinguishable from WT at permissive temperatures (Vögtle et al. 2018; Poveda-Huertes et al., 2020). Only upon shifting the temperature the Mas1 function is impaired followed by the accumulation of non-processed precursors in the matrix, which then aggregate and eventually cause the protective mtUPR. To explain this to the reviewer, we performed growth curves of wild-type and *mas1*^{ts} cells at 25°C that reveal no difference in growth between the two strains (**novel Supporting Figure S1A**). Thus, at permissive temperature the *mas1*^{ts} cells are as healthy as wild-type cells and only display a growth defect upon induction of MPP dysfunction by an increased growth temperature.

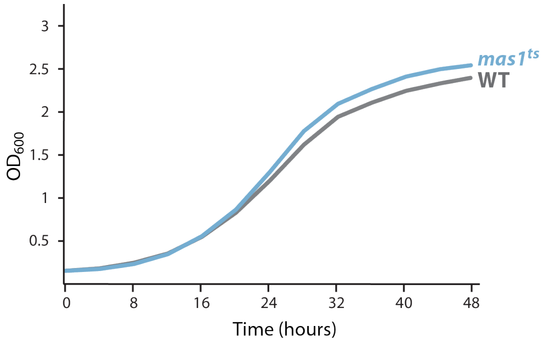

Novel Supporting Figure S1A. Growth curve of wild-type (WT) and *mas1*^{ts} cells on non-fermentable carbon source at permissive temperature (25°C) revealing that the *mas1*^{ts} mutant is growing comparable to wild-type cells.

Furthermore, we have now deleted several other genes coding for enzymes involved in lipid biosynthesis as requested by the reviewer, but did not detect any growth defects in the *mas1*^{ts} mutant (**Novel Supporting Figure S8A**). Thus, the growth defect of *mas1*^{ts} seems to be specific to changes in cardiolipin.

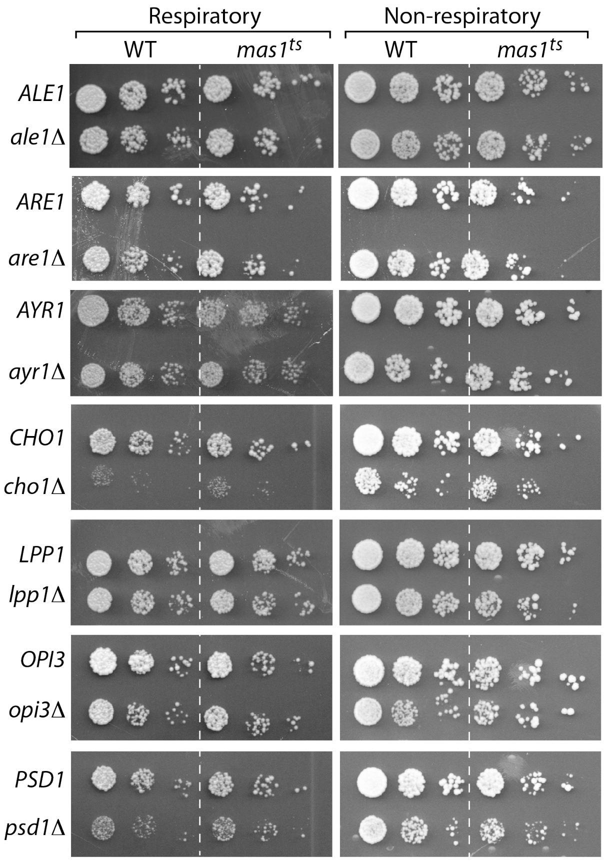

Novel Supporting Figure S8A. Analysis of growth behavior of indicated yeast strains. Deletion of genes coding for enzymes of several lipid synthesis pathways do not impact on growth of *mas1*^{ts} cells.

We have also generated a *taz1Δ mas1*^{ts} strain as requested by the reviewer. Deletion of *TAZ1* leads to a prominent growth defect in the *mas1*^{ts} background compared to WT (analyzed both on solid medium and in growth curves in liquid medium; **novel Figures 3B and 3D**). Thus, only mutations in CL biosynthesis (*CRD1*, *TAZ1*) result in a stronger growth defect of the *mas1*^{ts} mutant, clearly revealing that the effect is specific to CL.

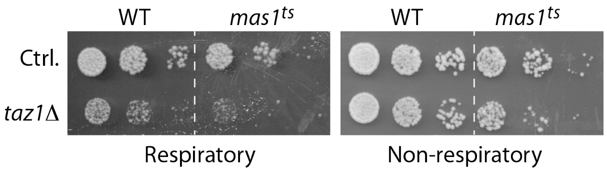

Novel Figure 3B. Growth test of wild-type and *mas1*^{ts} cells with additional deletion of *TAZ1* at 35°C. Cardiolipin remodelling is required for growth of *mas1*^{ts} cells upon induction of mtUPR.

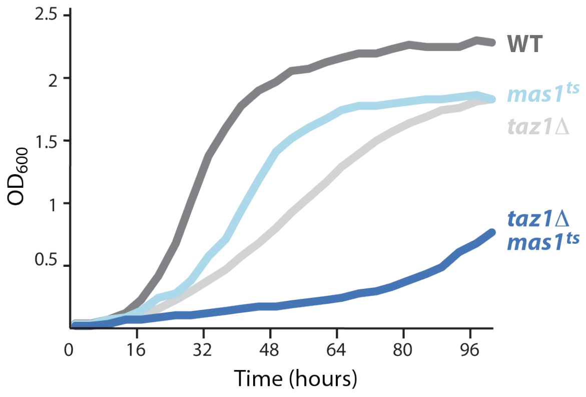

Novel Figure 3D. Growth curves of indicated strains at non-permissive temperature (35°C) confirming that cardiolipin remodelling by Taz1 is required for growth of *mas1*^{ts} cells.

5.An important part of the mtUPR are cytosolic events. In their in organello import set-up, the authors use rabbit retic. lysate as a cell-free synthesis system. This situation eliminates any effect of yeast cytosolic factors, which might play an important role in authentic protein import. Thus, I wonder whether a homogenous system (isolated organelles and cytosolic fraction from the same organism) would result in similar results.

> We have set-up and established a protocol for radiolabelling of precursors using yeast cytosolic ribosomes as requested by the reviewer. We have assessed import competence of Por1 precursor synthesized in yeast cytosolic extract, which shows the same result as before when using the rabbit reticulocyte lysate, fully supporting our findings (**Rebuttal Figure R7**).

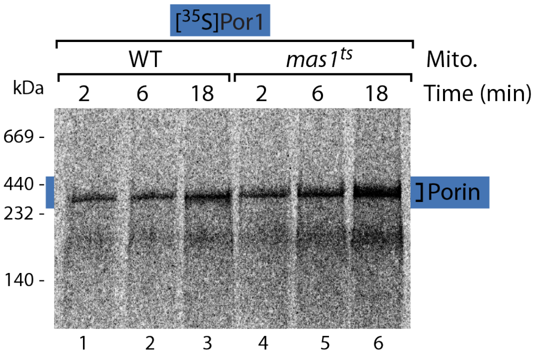

Rebuttal Figure R7. Import of radiolabeled Por1 precursors synthesized in yeast cytosolic extract. Import into *mas1*^{ts} mitochondria is enhanced upon mtUPR (induction for 10 hours).

Reviewer #3 (Significance (Required)):

This contribution suggest an alternative model for the sequence of events in progress of mtUPR. Such a new model might be of general interest to many readers. However, the current data does not support this claim.

> We are happy that the reviewer is of the opinion that our new model on the progression of mtUPR is of general interest to many readers. We strongly disagree that our data do not support our claims. We have addressed all points raised by the reviewer with novel experiments and all are fully supporting our novel mtUPR model.

References

Gebert N, Joshi AS, Kutik S, et al. Mitochondrial cardiolipin involved in outer-membrane protein biogenesis: implications for Barth syndrome. *Curr Biol.* 2009;19(24):2133-2139.

Harbauer AB, Opalińska M, Gerbeth C, et al. Mitochondria. Cell cycle-dependent regulation of mitochondrial preprotein translocase. *Science*. 2014;346(6213):1109-1113.

Li G, Chen S, Thompson MN, Greenberg ML. New insights into the regulation of cardiolipin biosynthesis in yeast: implications for Barth syndrome. *Biochim Biophys Acta*. 2007;1771(3):432-441.

Osman C, Voelker DR, Langer T. Making heads or tails of phospholipids in mitochondria. *J Cell Biol*. 2011;192(1):7-16.

Poveda-Huertes D, Matic S, Marada A, Habernig L, Licheva M, Myketin L, Gilsbach R, Tosal-Castano S, Papinski D, Mulica P, Kretz O, Kücükköse C, Taskin AA, Hein L, Kraft C, Büttner S, Meisinger C, Vögtle FN. An Early mtUPR: Redistribution of the Nuclear Transcription Factor Rox1 to Mitochondria Protects against Intramitochondrial Proteotoxic Aggregates. Mol Cell. 2020 Jan 2;77(1):180-188.e9. doi: 10.1016/j.molcel.2019.09.026.

Schlame M. Cardiolipin remodeling and the function of tafazzin. *Biochim Biophys Acta*. 2013;1831(3):582-588.

Suhm T, Kaimal JM, Dawitz H, et al. Mitochondrial Translation Efficiency Controls Cytoplasmic Protein Homeostasis. *Cell Metab*. 2018;27(6):1309-1322.e6.

Vögtle FN, Brändl B, Larson A, et al. Mutations in PMPCB Encoding the Catalytic Subunit of the Mitochondrial Presequence Protease Cause Neurodegeneration in Early Childhood. *Am J Hum Genet*. 2018;102(4):557-573.
Vögtle FN. Open questions on the mitochondrial unfolded protein response [published online ahead of print, 2020 Sep 22]. *FEBS J*. 2020;10.1111/febs.15569.

Vögtle FN, Keller M, Taskin AA, et al. The fusogenic lipid phosphatidic acid promotes the biogenesis of mitochondrial outer membrane protein Ugo1. *J Cell Biol*. 2015;210(6):951-960.

Xu Y, Schlame M. The turnover of glycerol and acyl moieties of cardiolipin. *Chem Phys Lipids.* 2014;179:17-24.
