# Supplementary material for: Increased mitochondrial protein import and cardiolipin remodelling upon early mtUPR
Source: PLoS Genet. 2021 Jul 2;17(7):e1009664. doi: 10.1371/journal.pgen.1009664 (PMC8282050; doi:10.1371/journal.pgen.1009664)
Supplement: Attachment — Submitted filename: Response to reviewers_2.pdf [file pgen.1009664.s014.pdf]

Reviewer #1: This is a revised version of the manuscript previously submitted to PROS Genetics. The authors added substantial amounts of new results, which could strengthen and improve the manuscript. Their observation on the effects of defective MPP processing in mas-ts mutant mitochondria is very interesting and would offer an important basis for future studies on cellular responses to the defects in the mitochondrial protein import and related processes. Therefore, I have no objection against the importance of the present work at the level of descriptive reports. My still-remaining concern is the authors' interpretation that the defective MPP processing causes the increase in the CL level, and that the increased CL level enhances the mitochondrial protein import efficiencies. I do not see that the logic behind this interpretation was experimentally and convincingly demonstrated here. Therefore, I suggest the authors distinguish what was experimentally demonstrated clearly and what is still at the level of speculation throughout the manuscript. Here are the points I suggest the authors consider.

> We thank the reviewer for this positive assessment of our revised manuscript. We have carefully read the manuscript and clearly indicated what was experimentally demonstrated. We want to emphasize that this is the first study showing that mitochondrial protein import is stimulated upon mtUPR. In addition we present the discovery that also CL is dynamically remodelled upon mtUPR. We suggest that this concomitant CL remodelling might play a role in the observed enhanced protein import. However, we do not claim that the increased import is exclusively caused by CL remodelling.

(1) It is understandable that the deletion of the CRD1 gene or decrease in the CL level caused import defects because the decreased CL level should affect structures and functions of inner membrane protein complexes in general. However, this does not mean that the reverse relationship is expected, that is, it was not demonstrated that the increased CL level directly enhanced the protein import efficiencies. Negative synergetic effects of the deletion of TAZ1 and mas1-ts mutation may support the idea that accumulated presequence-containing precursor proteins could cause structural perturbation of the inner membrane, but does not again support the reverse effects that the increased CL level would enhance the protein import efficiencies. In this sense, the authors' new findings that the increase in the levels of Ups1 and Pgs1 could explain the increased CL level are important. I suggest that the authors should increase the levels of Ups1 and Pgs1 in wild-type cells, which would increase the CL level, and then test the possible enhancement of the protein import.

> The suggestion by the reviewer to analyze protein import in mitochondria with artificially increased CL synthesis is interesting. However, increased expression of Ups1 does not result in an increase in CL levels (see Osman et al., J. Cell Biol. 2009, Figure 6D and E). Furthermore, CL changes in the *mas1*^{ts} mutant go beyond an increased abundance and also include dynamic remodelling of the acyl chains, which cannot be mimicked by overexpression of a synthesis enzyme. Therefore, increased CL levels in the WT even if they could be achieved, will not resemble the in vivo situation under mtUPR conditions and will not be applicable to study the correlation between CL and import machineries.

(2) The authors found that the steady-state level of mtHsp10 increased upon defective MPP processing, which may complement the results of the in vitro import. However, the level of mtHsp10 has a problem that mtHsp10 is a heat-shock protein. Therefore, the level of

mtHsp10 should be tested after changing its promoter with an unrelated one to minimize the effects of the heat-shock response but to reflect import efficiency in vivo. An arising related question is what about the protein levels of mitochondrial proteins for the other import pathways are. It looks that Por1 did not change its levels between WT and mas1-ts cells. How about Tim9 and AAC?

> The heat-shock was applied to both the wild-type and *mas1*^{ts} cells, so that differences in Hsp10 protein levels cannot be explained by the heat-shock, but are specific to the *mas1*^{ts} mutant. Changing the promoter region of Hsp10 would affect regulation of its endogenous expression by transcription factors that induce the mtUPR response and would therefore abrogate this response. Increase of Hsp10 in vivo is caused by i) its up-regulation during mtUPR and ii) enhanced import. Protein levels of other mitochondrial proteins e.g. Tim9 and AAC, do not change as they are not regulated in the transcriptional mtUPR response. Therefore, their steady state levels under mtUPR conditions will not increase because even if mitochondrial protein import is enhanced, there will not be more precursor proteins available for import.

(3) It is still not clear how the increased CL level further increases the import ability of mitochondrial import machineries, although this may be beyond the scope of this study. The new results on the digitonin extractability of the TIM22 complex are interesting, but should not be used for over-interpretation. For example, what about the TOM complex and SAM complex?

> We see different extractability of the TIM22 and TIM23 complexes in the inner membrane. CL levels are much higher in the inner membrane compared to the outer membrane, which makes the analysis of the effect on the OM translocases challenging. We phrase our conclusion of this part with care as we are aware that we are just at the beginning of the detailed analysis to decipher our new and highly interesting observation of increased protein import upon mtUPR. We agree with the reviewer that further analysis is beyond the scope of this first report.

Reviewer #2: The authors improved their text, clarified unclear points, and added some more experiments. However, as indicated in detail below, the relevance of the apparent changes in the cardiolipin (CL) levels and composition to difference in protein import efficiency is still not convincing. Some of the experiments lack crucial controls and/or does not support the causative effect of altered CL levels on protein import.

> We are surprised that the reviewer is of the opinion that our revised version contains "some more experiments". The first reviewer here comments: "The authors added substantial amounts of new results, which could strengthen and improve the manuscript." The comment of the third reviewer is: "I am very happy to see that they addressed all the issues raised and provided a wealth of additional experiments which make the manuscript much stronger and clarified further several points in their work."
This is a discrepancy we do not understand.

Main comments:

1. Fig. 2B: The levels of CL in the mas1-ts strain after 4 hrs at 37°C are similar to those of WT without shift to 37°C. Thus, if the shift to 37°C under these conditions should represent "stress induction", I cannot detect any compensation changes in the CL levels as compared to normal non-stressed conditions that should support enhanced import efficiency.

> We compare stress induction by temperature shift of wild-type and *mas1^ts^* cells. When we compare the kinetics, a clear dynamic change in CL levels is observed: While in WT CL levels decrease after 4 hours, CL levels increase in the *mas1^ts^* mitochondria. It is important to point out that we have to compare *mas1^ts^* with WT that both have been exposed to 4 hours heat-shock and not with WT mitochondria isolated at permissive temperature. Such a comparison would of course be inappropriate. In addition, not only CL levels, but importantly also the acyl chains change upon mtUPR. This CL remodelling is clearly showing differences in WT and the *mas1^ts^* mutant after 4 hours. All of these data are shown in Figure 2 of our revised manuscript.

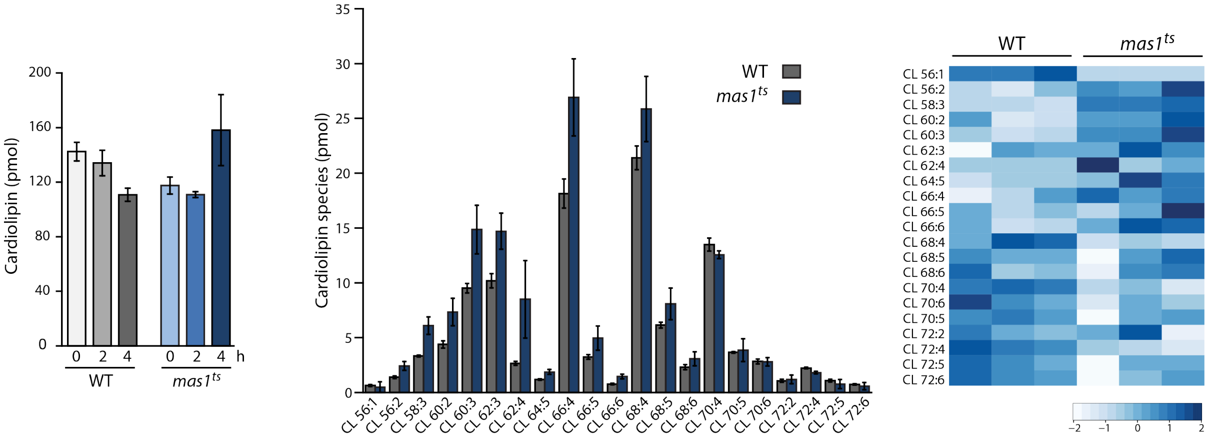

Figure 2B, D and E. B (left panel), Cardiolipin (CL) is dynamically changing upon heat-shock with a decrease in wild-type (WT) and an increase in *mas1^ts^*. D (middle panel), Quantification of cardiolipin in WT and *mas1^ts^* mitochondria isolated after growth under non-stress conditions, or after mtUPR induction for 2 or 4 hours. n=3, data represent means ± SEM. E (right panel), Heatmap of distribution of different CL subspecies standardized to total CL content per sample in WT and *mas1^ts^* mitochondria isolated from cells shifted for 4 hours to non-permissive growth temperature. Shown are three biological replicates for each strain.

2. Fig. 2C and lines 214-219: The increase in the levels of CDP-DAG is observed also for WT cells after 2 hrs at 37°C. In addition, the change in the CDP-DAG for the mas1ts strain within the first two hrs at 37°C is only 0.35 pmol whereas the increase in the levels of CL between 2 to 4 hrs is about 30 pmol (100 fold). The authors describe these changes as "This suggests that cells increase the biosynthesis of CL upon mtUPR and rapidly convert its precursor into CL". However, if this is the case, why there is no change in CL amount also after 2 hrs at 37°C?

> This question was already raised in the first round of reviewer comments by reviewer 2 (now reviewer 3) and we addressed this in our previous response to reviewers letter. For the reviewer's convenience we add it here again:
CDP-DAG is a precursor for several other phospholipids besides cardiolipin including phosphatidyl choline (PC) and phosphatidyl inositol (PI). The increase in CDP-DAG in the wild-type could therefore be due to general lipid modulation upon the applied heat shock. Furthermore, CDP-DAG is a lipid precursor that is only transiently present and rapidly further modified to generate the indicated phospholipid species. It is short lived and of low abundance, and does not accumulate in mitochondria under normal conditions (Osman et al., JCB 2011), which is why it is only detected in low amounts in our lipidomics analyses.

In addition, according to the authors claims, the CL levels should be even higher after 10 hrs and then decline again towards the 20 hrs stage ("late stages"). However, such data is not provided as Fig. 2B shows data only for 2 and 4 hrs.

> Again this question was raised by reviewer 2 (now reviewer 3) in the first reviewer comments and we have provided lipidomics analyses of mitochondria isolated after 10 hours of mtUPR induction in the revised manuscript demonstrating alterations in CL subspecies also at this later time-point (Figure S7).
Again please find below our previous answer in the first round of revision:
The lipidomics analyses were performed after 2 and 4 hours to be able to detect the initial start and the dynamics of the changes in CL. CL is turned over much slower than other phospholipids so that once CL is assembled it remains inert to degradation (Xu and Schlame, 2014). Due to its long half-life changes in CL should indeed persist over a longer period of time as proposed by the reviewer. We have now addressed this by detailed lipidomic analysis of CL species after prolonged heat-shock of 10 hours, e.g. the conditions upon which import is strongly increased. Cardiolipin species clearly show a different remodelling of their CL acyl chains including differences in length and unsaturation, compared to WT also after 10 hours of heat shock (novel Figures S7B-D). This demonstrates that changes in CL are present under the conditions in which increased protein import is observed.

3. Fig. 2E and S7B-D: It is not clear what this panel represents. Are those absolute numbers or differences to cells that were not shifted to 37°C? What is the scale of -2 to +2? In addition, it seems that the trend in the relatively short species of CL is different between 4 to 10 hrs (compare Figs. 2E to S7B) although both conditions should support more import.

> Both figure legends from Fig.2E and S7B-D describe that we compare wild-type and *mas1^ts^* cells grown for 4 hours at 37°C (Fig. 2E) or 10 h at 37°C (S7B-D). We always compare wild-type and *mas1^ts^* mitochondria that were isolated from cells grown at the same temperature. Comparing a shifted *mas1^ts^* strain with WT grown at permissive temperature would not be accurate.
In respect to the question whether those are absolute values: we indeed show absolute values (in pmol) for the total CL levels (in Fig. 2B) as well as for all CL species (Fig. 2D) to provide information of absolute CL content. In addition to this, we chose to show heat-maps to visualize the relative abundance (mol%) of the different CL subspecies within these samples (Fig. 2E and Fig. S7B-D). To generate these heat-maps, only CL species in mol%

where taken into account, meaning that the values have been standardized to each sample and the total CL content within each sample was set to 100%. This way, absolute differences between the CL content of different samples will not be apparent, but instead the relative percentage of each CL subspecies within the CL pool will be visible. In respect to the scale: values reflect relative changes (log2(fold)), scaled and centered for each lipid species. To clarify where absolute values versus relative changes are depicted, we have added the following information to the respective Figure Legends for Figure 2:
(B) …"Non-standardized, absolute values in pmol measured in total lipid extracts from isolated, highly purified mitochondria are shown."
(C) …"Non-standardized, absolute values in pmol measured in total lipid extracts from isolated, highly purified mitochondria are shown."
(D) …"Non-standardized, absolute values in pmol for all CL species detected in total lipid extracts from isolated, highly purified mitochondria are shown."
(E)…"Heatmaps were generated using standardized values in mol% and thus total CL content in each individual sample was set to 100% to represent relative CL species distribution within each sample. Relative changes, scaled and centered for each CL species, are depicted." (This information for Figure legend 2E was also added to S7B-D.)

4. Fig. 3E: The figure shows apparent higher levels of the TIM22 complex in the control organelles upon solubilization with 0.5% Digitonin. However, without appropriate control (like SDS-PAGE analysis followed by Western blotting) of the very same samples side-by-side we do not know if this is a difference in solubilization or the outcome of loading variable amounts. Generally, I am not sure that detecting less material under these conditions can indicate higher stability of the monitored complex or represents an overall different behavior of the solubilized membrane.

> We have performed analysis of TIM22 complex subunits on SDS-PAGE followed by western blotting as requested by the reviewer. The protein steady state levels do not change after shift to non-permissive growth temperature for 4 hours fully supporting our conclusions. Furthermore, none of the components of the import machineries are changed even 10 hours after mtUPR induction (see Figure S6B). Furthermore, we do not conclude that the different extraction profile of the TIM22 complex in WT and *mas1*^{ts} mitochondria indicate higher stability as stated by the reviewer, but we conclude from this experiment that the lipid composition around the translocase can be modulated upon mtUPR.

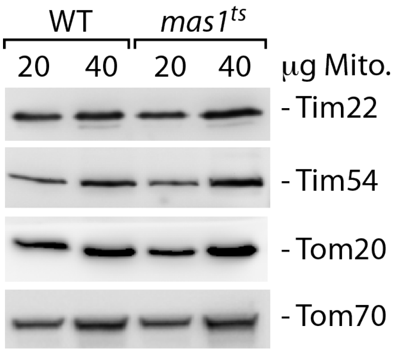

Novel Figure S8C. Steady state protein levels from mitochondria isolated after growth for 4 h at 37°C. Subunits of the TIM22 complex (Tim22 and Tim54) do not change, also subunits of the TOM complex (Tom20 and Tom70) are not altered.

5. The authors write in the M&M section that import of Porin and Tom20 was analyzed after solubilization with 0.3% [w/v] digitonin. On the other hand, the authors claimed that lower conc. of digitonin (like 0.5%) can extract less complexes from the tas1ts strain (Fig. 3E). Hence, the authors should comment whether differences that are observed under these conditions indeed reflect the variation in import efficiency or in solubilization efficiency.

> Analysis of Por1 and Tom20 on BN-PAGE is standardly performed with lower digitonin conditions. For example the Tom20 receptor is only peripherally associated to the TOM core complex (which again is stable also in 1% digitonin) and falls of upon solubilization with higher detergent amounts (Meisinger et al., MCB 2001). The finding that the solubilization efficiency requires higher amounts of digitonin in *mas1*^{ts}, implies that the observed import effects could even be stronger.

Minor issues:

a. Fig. 1C: what are the two bands at about 100 kDA? These complexes, which might represent the Tim9/10 and/or Tim9/10/12 complexes, are formed faster in WT rather than slower. The authors should comment on this in the text.

> The 100 kDa bands are representing radiolabelled Tim9 precursor proteins assembled in the soluble Tim9/Tim10 complexes in the intermembrane space. From there the precursor assembles with the membrane embedded TIM22 translocase, which seems to have a higher capacity to incorporate the Tim9 precursor in the *mas1*^{ts} mutant. The transfer of the soluble TIm9/Tim10 complex to the TIM22 complex seems to be decelerated in the WT and therefore we see accumulation of the soluble complex. This observation fully supports our model. We have added additional information about the soluble complex now in the revised manuscript.

b. Line 171: the authors write “our conditional mas1ts mutant allows mild and reversible stress induction”. However, they did not show that cells that were initially transferred to the non-permissive temperature and then back to permissive temperature have wild type-like import behavior. Hence, I suggest that they will rephrase their claim.

> The sentence referred to by the reviewer is a description of our *mas1*^{ts} model and referenced correctly (Poveda-Huertes et al., Mol. Cell 2020).

c. Fig. 2G: I do not see a difference in the levels of Pgs1 (40 µg) between WT and mas1ts strains. The authors should change their text or show more convincing data.

> We have repeated the steady state analysis several times and always detected increased Pgs1 protein levels. We have provided a new blot in the revised version of the manuscript that shows the increased Pgs1 level in mas1ts in better quality.

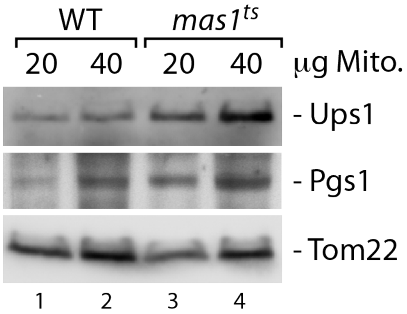

Novel Figure 2G. Immunoblot analysis of wild-type (WT) and *mas1^{ts}* mitochondria isolated from cells shifted to non-permissive temperature for 10 hours. Tom22 serves as loading control.

d. Figs. 3C-D and S1A&B: Do these curves represent continuous monitoring of the OD? If yes, this should be indicated in the M&M section or in the legends. If not, the values of the distinct time points should be indicated.

> In this growth analysis the OD is measured every 5 min during the indicated time period. We have added this information in the materials and methods chapter as requested by the reviewer.

e. Line 784: should be "in (B)-(E)".

> We thank the reviewer for careful reading of the figure legends. We have changed the description of the figure.

f. Legend to Fig. S7: The description of panels C and D are referred to panels B and C.

> We have changed the description of the figure.

g. Fig. S8B: I do not see a difference in the behavior of the TIM23 complex between the two strains. The authors should change their text or show more convincing data.

> We provide a novel BN-PAGE analysis in the revised version using a shorter exposure time and with improved quality of the TIM23 complex extraction.

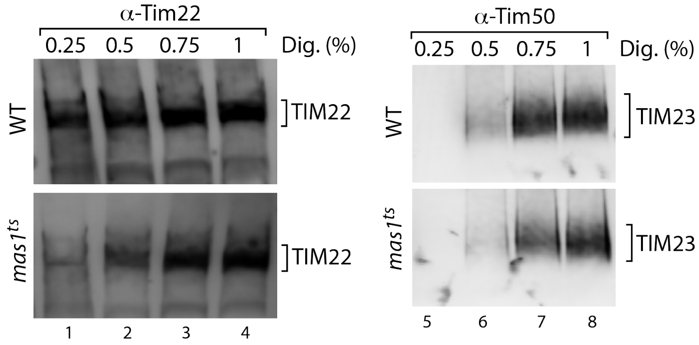

Novel Figure S8B. Analysis of translocases in wild-type (WT) and *mas1^{ts}* mitochondria isolated from cells grown for 10 hours at non-permissive temperature. Samples were solubilized with indicated concentrations of digitonin and analyzed by Blue-native PAGE and immunodecoration.

Reviewer #3: The authors have revised their work in light of the previous comments. I am very happy to see that they addressed all the issues raised and provided a wealth of

additional experiments which make the manuscript much stronger and clarified further several points in their work. One of the points I raised (major point 3 on levels of CDP-DAG) is still in my opinion not very convincingly addressed. However, as the authors really have substantiated all other supporting evidence with additional work I am happy to recommend acceptance and publication of this very interesting work.

> We are very happy about this positive evaluation of our work and thank the reviewer for his/her comment.
