# Supplementary material for: Increased mitochondrial protein import and cardiolipin remodelling upon early mtUPR
Source: PLoS Genet. 2021 Jul 2;17(7):e1009664. doi: 10.1371/journal.pgen.1009664 (PMC8282050; doi:10.1371/journal.pgen.1009664)
Supplement: Attachment — Submitted filename: Response to reviewers_3.pdf [file pgen.1009664.s015.pdf]

Reviewer #1: This is the second revised version of the manuscript submitted to PROS Genetics. I am a bit disappointed to see that the authors have not made any further efforts to clear my remaining concerns this time.

(1) My major concern in the last round of revision was …"my still-remaining concern is the authors' interpretation that the defective MPP processing causes the increase in the CL level, and that the increased CL level enhances the mitochondrial protein import efficiencies. I do not see that the logic behind this interpretation was experimentally and convincingly demonstrated here." The authors' answer to this concern was.. "we suggest that this concomitant CL remodelling might play a role in the observed enhanced protein import. However, we do not claim that the increased import is exclusively caused by CL remodelling." However, I do not see the authors' claim of "this concomitant CL remodelling might play a role in the observed enhanced protein import", even if this is just a suggestion, is based on the experimental results, and thus inappropriate here.
For example, the authors stated "if modulation of cardiolipin upon mtUPR and the concomitant changes in the lipid environment around the translocases are a requirement for the observed stimulation of protein import upon mtUPR" (page 12), but the performed experiment was designed to see the effects of crd1Δ mutation in wild type and mas1-ts strains, confirming that the CL level decrease would abrogate the import efficiencies, but not demonstrating that the CL increase promotes the import efficiencies. The mechanism of even the increase in the levels of Ups1 and Pgs1 is still not clear. Therefore, the authors' conclusion of "this modulation in the lipid environment likely stabilizes the mitochondrial import complexes thereby increasing the protein import capacity into the organelle under stress conditions" (page 12) was not experimentally supported. Therefore, the authors had better withdraw this "suggestion" from the manuscript if the manuscript stays as it stands.

> We have removed the sentence (results, page 12) from the manuscript as requested by the reviewer.

(2) I still do not understand that why mas1-ts mutant is more suitable for analyzing the physiologically relevant stress for mitochondria. Accumulation of non-cleaved precursor proteins in the matrix could be harsher to mitochondria than mitochondria with slightly reduced membrane potential, and the inhibition of presequence-cleavage would not occur under physiological conditions. In addition, even with a control of wild-type cells at elevated temperature, the effects of the shift to non-permissive temperature could give profoundly complicated synthetic effects with mas1-ts mutation to Hsp10 as well as to the cell. Therefore, the authors had better soften the description on the disadvantage of the previous studies using the conditions of the reduced membrane potential. The authors can simply expect different effects arising from the different stress conditions for mitochondria.

> Previous studies did not induce mtUPR by a slight reduction of the membrane potential as stated by the reviewer, but used chemical inhibitors that result in a complete dissipation. We have provided comparison of stress induction using our *mas1^{ts}* mutant with "classic" mtUPR inducers in our first Response to Reviewer letter and we have added the data here again (see below). It is clear that our *mas1^{ts}* system induces a mild stress as the cells are still able to grow and divide. In contrast, the classical mtUPR inducers immediately completely impair cell growth. We also compare the membrane potential in our *mas1^{ts}* mutant with one of the classic inducers (menadione). While the membrane potential in our *mas1^{ts}* mutant is not affected, it

is not detectable anymore upon classical mtUPR induction. Therefore, the *mas1^ts^* mutant is a milder system and very suitable for investigation of mtUPR.

Furthermore, the reviewer claims that "the inhibition of presequence-cleavage would not occur under physiological conditions". However, analysis of impaired presequence-cleavage is highly relevant given that many studies identified this impairment in human diseases:

a. Mutations in PMPCB Encoding the Catalytic Subunit of the Mitochondrial Presequence Protease Cause Neurodegeneration in Early Childhood (Vögtle at al., Am. J. Hum. Genet. 2018)
b. Amyloid-β peptide induces mitochondrial dysfunction by inhibition of preprotein maturation. (Mossmann et al. Cell Metab., 2014; Kücükköse et al., FEBS J. 2021)
c. Mutations in the substrate binding glycine-rich loop of the mitochondrial processing peptidase-a protein (PMPCA) cause severe mitochondrial disease (Jobling et al., Brain 2015; Joshi et al., Cold Spring Harbor Mol Case Stud. 2016)

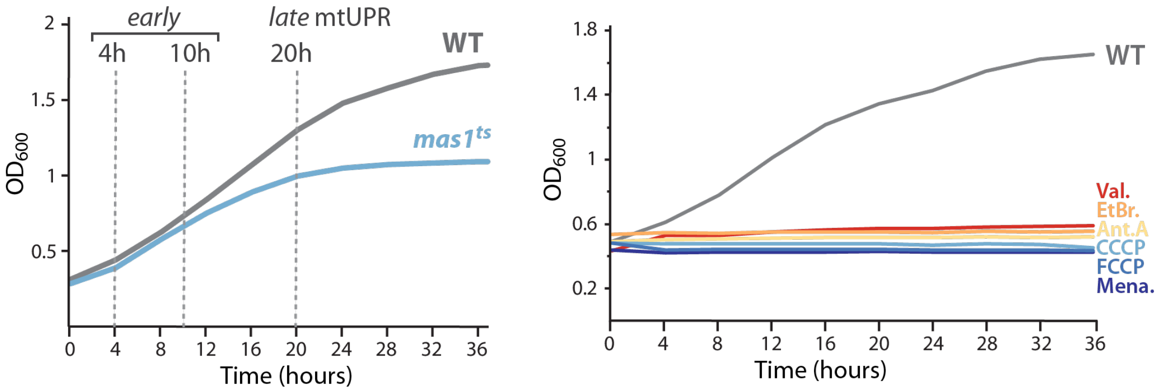

Novel Supporting Figure S1B and Rebuttal Figure R1. Growth curve of wild-type (WT) and *mas1^ts^* cells on non-fermentable carbon source at non-permissive temperature (37°C) revealing that the *mas1^ts^* mutant is dividing after 20 hours of induction (left panel). Growth curves in the right panel display previously used triggers for induction of mtUPR in the literature that all immediately inhibit cell division (Val., valinomycine 2 μM; EtBr., Ethidium bromide treatment 60 μg/mL; Ant. A, Antimycin A 1 μM; Mena, menadione 0.3 mM; CCCP, Carbonyl cyanide m-chlorophenyl hydrazone 10 μM; FCCP, Carbonyl Cyanide. P-(Trifluoromethoxy) Phenylhydrazone 10 μM).

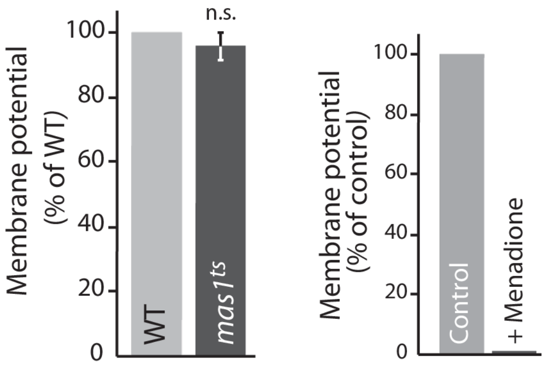

Novel Supporting Figure S4E and Rebuttal Figure R2. Membrane potential measurement using mitochondria isolated after growth at 37°C for 20 hours (left panel). The membrane potential in the *mas1^ts^* mutant is comparable to wild-type. In contrast, mitochondria isolated after treatment with 0.3 mM menadione do not have a measurable membrane potential anymore.

In summary, simultaneous observation of the effects on the import efficiencies and lipid compositions under the conditions of the mas1-ts mutation are interesting, but mechanistic reasoning of this observation has not been experimentally or properly tested. Therefore, this manuscript remains at a descriptive level, and interpretation of the observation that the defective MPP processing causes the increase in the CL level, and that the increased CL level enhances the mitochondrial protein import efficiencies is not logically appropriate. The authors had better emphasize the significance of the observation of the two phenomena, the change in lipid composition and enhancement of protein import efficiencies, that were previously thought to be unrelated. I think this is the point that is novel enough to be published.

> We have addressed all additional points raised by the reviewer and we thank the reviewer for his/her statement that our study is novel enough for publication.

Reviewer #2: The authors addressed my concerns and the manuscript can be published.
